# RNF128 promotes gastric cancer progression by inhibiting autophagy-dependent ferroptosis through Beclin1 ubiquitination

**DOI:** 10.1038/s41420-025-02488-8

**Published:** 2025-04-19

**Authors:** Zhenguo Zhu, Qishuai Chen, Siyi Song, Shi Peng, Huanxin Ding, Bingjun Li, Chuxuan Liu, Xin Jin, Linchuan Li, Jiankang Zhu, Guangyong Zhang

**Affiliations:** 1 Department of General Surgery, The First Affiliated Hospital of Shandong First Medical University & Shandong Provincial Qianfoshan Hospital, Jinan, China; 2 Laboratory of Metabolism and Gastrointestinal Tumor, The First Affiliated Hospital of Shandong First Medical University & Shandong Provincial Qianfoshan Hospital, Jinan, China; 3 Shandong Provincial Engineering Research Center of Minimally Invasive Diagnosis and Treatment for Digestive Diseases, Jinan, China; 4 Medical Center for Digestive Diseases, The First Affiliated Hospital of Shandong First Medical University & Shandong Provincial Qianfoshan Hospital, Jinan, China; 5 Department of General Surgery, Zibo Central Hospital, Zibo, China; 6 Department of Anesthesiology and Perioperative Medicine, The First Affiliated Hospital of Shandong First Medical University & Shandong Provincial Qianfoshan Hospital, Jinan, China; 7 Department of General Surgery, the First Affiliated Hospital of Shandong First Medical University & Shandong Provincial Qianfoshan Hospital, Cheeloo College of Medicine, Shan-dong University, Jinan, China

**Keywords:** Gastric cancer, Pexophagy, Ubiquitylation

## Abstract

As an important protein post-translational modification process, ubiquitination plays an indispensable role in the regulation of gastric cancer (GC) occurrence and development. And recent studies have demonstrated that this modification is closely related to regulated cell death. This suggests that our therapeutic approach to inhibit the malignant progression of GC by regulating the intracellular death mode through ubiquitination modification becomes possible. Although ubiquitination modification has been well described in some tumorigenesis, its potential role and specific mechanisms are still unknown. In the present study, we identified RNF128, an E3 ubiquitin ligase with a RING structural domain, whose expression was significantly increased in GC. In-depth studies showed that knockdown of RNF128 significantly inhibited GC cell proliferation and increased intracellular autophagic flux and lipid peroxidation production, and we hypothesized that autophagy-dependent ferroptosis might be the main mode of death mediated by RNF128. Mechanistically, RNF128 directly binds and ubiquitinates degradation of Beclin1 through its PA structural domain and significantly inhibits the Beclin1/solute transport family 7 member 11(SLC7A11)/glutathione peroxidase 4(GPX4) axis. Taken together, our study reports for the first time that RNF128 acts as a tumor promoter to inhibit autophagy-dependent ferroptosis in GCs by targeting Beclin1. These data provide new insights into the activation of intracellular ferroptosis to inhibit malignant tumor progression and are expected to provide a new strategy for molecular therapy in clinical GC patients.

## Introduction

GC is a widespread malignant gastrointestinal tumor that is prevalent in East Asia, particularly in China [1, 2]. With improvements in medical treatment, the prognosis of patients with GC has improved [3–5]. However, its recurrence and metastasis rates remain high. Furthermore, the underlying causes of GC development is still not fully understood. Therefore, to identify new target molecules for therapeutic strategies, it is necessary to elucidate the molecular mechanisms underlying GC progression.

Ubiquitination is a highly efficient protein degradation pathway that functions as a special type of post-translational modification [6–10]. Moreover, ubiquitination modifications can directly affect protein activity and localization and regulate several cellular activities, including the cell cycle, apoptosis, transcriptional regulation, DNA damage repair, and immune response, which are crucial in the progression of many tumors [11–14]. Ring Finger Protein 128 (RNF128) is a member of the E3 ubiquitin protease family and contains an N-terminal RA, a RING finger, and a C-terminal Disordered domain. RNF128 was initially referred to as a gene associated with lymphocytes, given its role in inhibiting T cell function and transcription factor production [15, 16]. Recent studies have shown that low expression of RNF128 in some tumors promotes tumor progression through the Wnt/β-catenin pathway [17, 18]. However, RNF128 is upregulated and promotes malignant tumor progression through the Hippo and epidermal growth factor receptor/MEK/extracellular signal-regulated kinase pathways [19, 20]. Notably, RNF128 plays different or even diametrically opposite roles in various tumors. However, the role and mechanism of action remain largely unknown. By combining data from previous studies in our laboratory and The Cancer Genome Atlas (TCGA) database, we found that the expression of RNF128 was elevated in GC tissues. Therefore, we aimed to investigate the role of RNF128 in GC tissues.

Aberrant post-translational modifications can promote anticancer drug resistance, cancer progression, and metastasis [21–27]. Furthermore, more studies have shown that ubiquitination-based post-translational modifications are crucial in regulating ferroptosis sensitivity in several solid tumors [28–31]. Autophagy-dependent ferroptosis is a form of regulated cell death that relies on autophagy to induce ferroptosis and ultimately inhibit tumor progression [32, 33]. In particular, Beclin1 is vital in the induction of autophagy-dependent ferroptosis [34]. Beclin1 can block the production of glutathione (GSH) by binding directly to and inhibiting the activity of solute carrier family 7 member 11 (SLC7A11), thereby causing excessive accumulation of lipid peroxides, ultimately leading to ferroptosis [35]. In this study, we explored the effect of RNF128 on the biological behavior of GC cells using clinical specimens and in vitro cellular and in vivo animal assays. To our knowledge, this is the first study to demonstrate that RNF128 promotes the malignant progression of GC by inhibiting the Beclin1/SLC7A11/ GPX4 axis.

## Results

### RNF128 was significantly up-regulated in GC cells

**Fig. 1:**
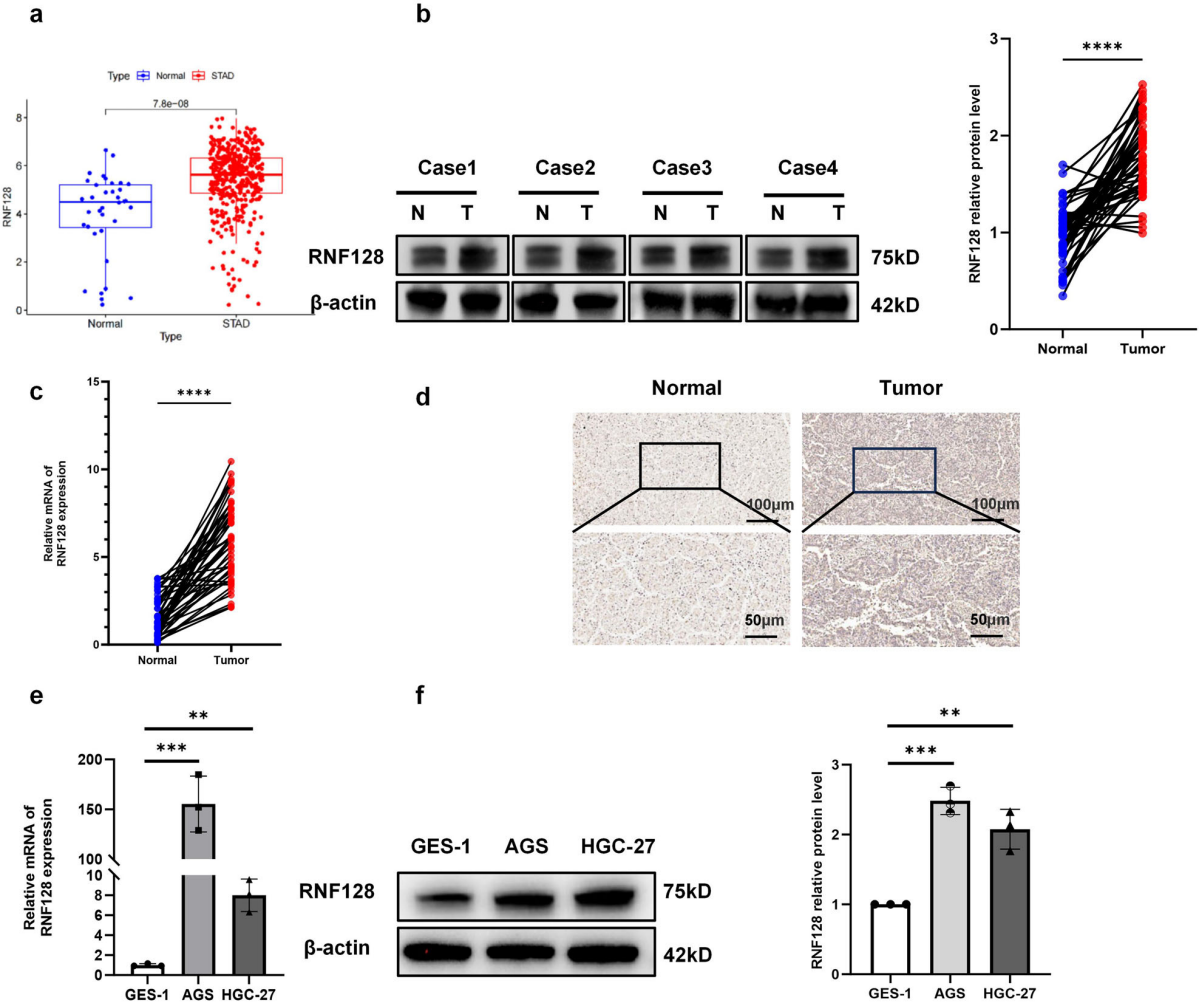
RNF128 is upregulated in GC. **a** Expression of RNF128 in TCGA database. **b** Expression of RNF128 protein in 50 GC tissues and matched adjacent tissues (*n* = 50, independent experiments). **c** Expression of RNF128 mRNA in 50 cases of GC tissues and matched adjacent tissues (*n* = 50, independent experiments). **d** IHC detection of RNF128 expression in GC tissues and matched adjacent tissues (*n* = 50, independent experiments). **e**, **f** RNF128 mRNA and protein expression in GES-1 and commonly used GC cell lines (*n* = 3, independent experiments). ****p < 0.0001, ***p < 0.001, **p < 0.01.

First, we analyzed the expression of RNF128 in GC cells using the TCGA public database and found that RNF128 was significantly upregulated in GC cells (Fig. 1a). It has been reported in the literature that different RNF128 isoforms have different effects on p53 stability in Barrett’s oesophagus cells [36]. However, we found no significant difference in RNF128 Iso1 levels in GC and matched adjacent tissues (Fig. S1a). In contrast, the protein level of RNF128 Iso2 was significantly higher in GC tissues than in matched adjacent tissues (Fig. 1b). In the follow-up study, we referred to RNF128 Iso2 as RNF128.

In order to deeply investigate the expression of RNF128 in GC cells, we performed Q-PCR and immunohistochemistry for RNF128 in GC tissue samples. The expression content of RNF128 in GC was found to be significantly higher than that in matched adjacent tissues (Fig. 1c, d). It is well known that adenocarcinoma accounts for the largest proportion of various subtypes of GC, accounting for about 90 percent. Compared with other subtypes, undifferentiated carcinoma is the most poorly differentiated, highly malignant and rapidly progressive. Therefore, in the in vitro experiments, we selected the corresponding adenocarcinoma and undifferentiated carcinoma GC cell lines, i.e., AGS and HGC-27, for subsequent experiments. Compared with the gastric epithelial cell line GES-1, both mRNA and protein levels of RNF128 were significantly higher in GC cells (Fig. 1e, f). Therefore, the expression of RNF128 was significantly elevated in GC.

### RNF128 promotes GC cell proliferation

To further investigate the effect of RNF128 on biological behaviors in GC cells, we established RNF128 knockdown and overexpression cell models by plasmid transfection. We performed Q-PCR and Western blot analyses to verify transfection efficiency (Fig. S2a–e). The knockdown efficiency of shRNF128-3 was the best, so we used it for subsequent experiments and denoted it shRNF128.

**Fig. 2:**
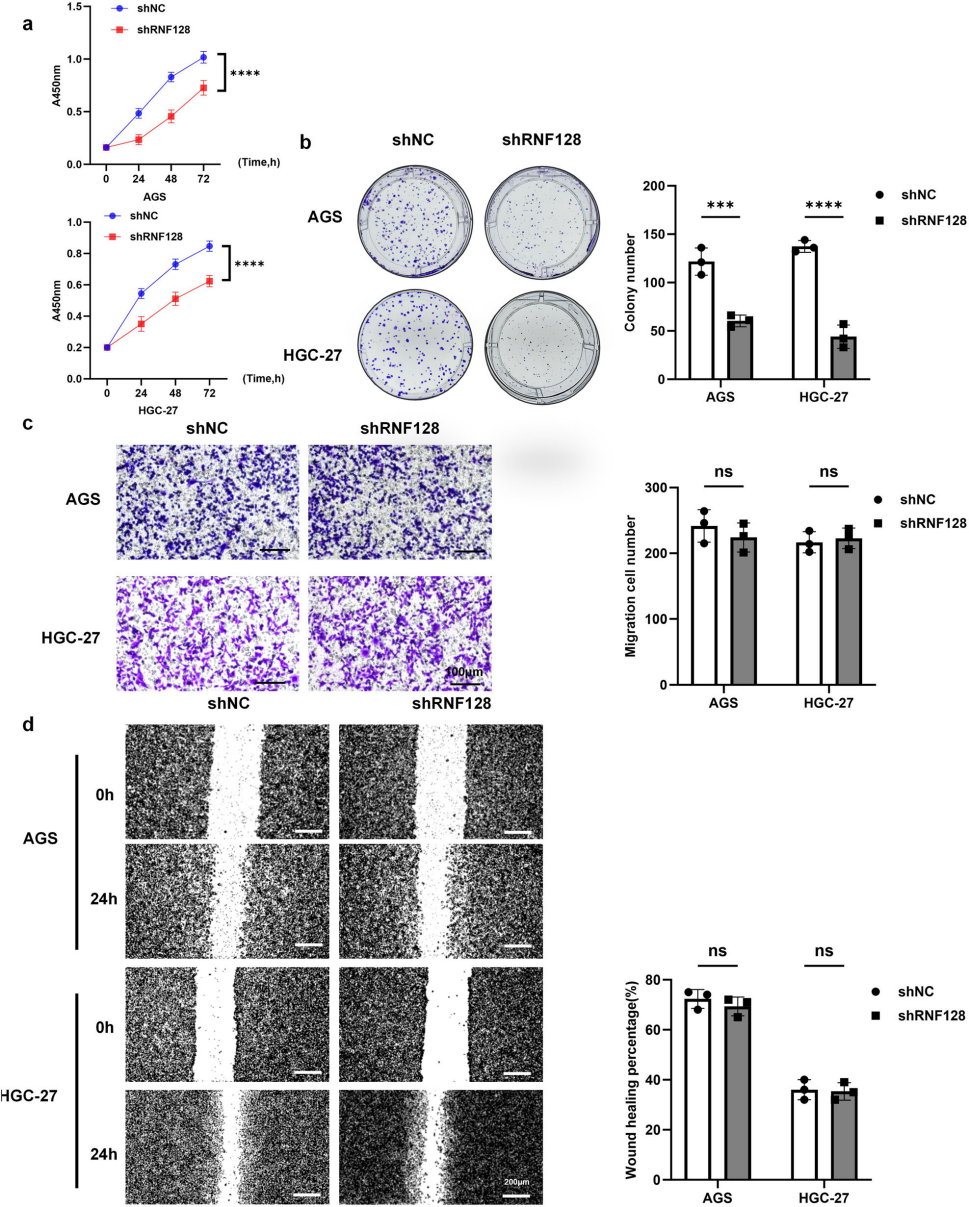
RNF128 knockdown inhibited the proliferation of GC cells. **a** CCK-8 assay to detect the cell viability of GC cells upon RNF128 knockdown. **b** Colony formation assay to detect the proliferation ability of GC cells upon RNF128 knockdown. **c**, **d** Transwell assay and cell scratch assay to detect the migration ability of GC cells at RNF128 knockdown. (*n* = 3, independent experiments), ****p < 0.0001, ***p < 0.001, ns not significant.

We confirmed that RNF128 knockdown significantly inhibited the viability and proliferation of GC cells through Cell Counting Kit-8 (CCK-8) and colony formation assays (Fig. 2a, b). However, transwell assay and cell scratch assay showed that the migration of GC cells was not significantly altered after knockdown of RNF128 (Fig. 2c, d). On the contrary, overexpression of RNF128 significantly promoted the viability and proliferation of GC cells (Fig. S3a, b). And it had no obvious effect on the migration of GC cells (Fig. S3c, d). These results confirmed that RNF128 promoted cell viability and proliferation of GC cells.

### Knockdown of RNF128 promotes autophagy-dependent ferroptosis in GC cells

**Fig. 3:**
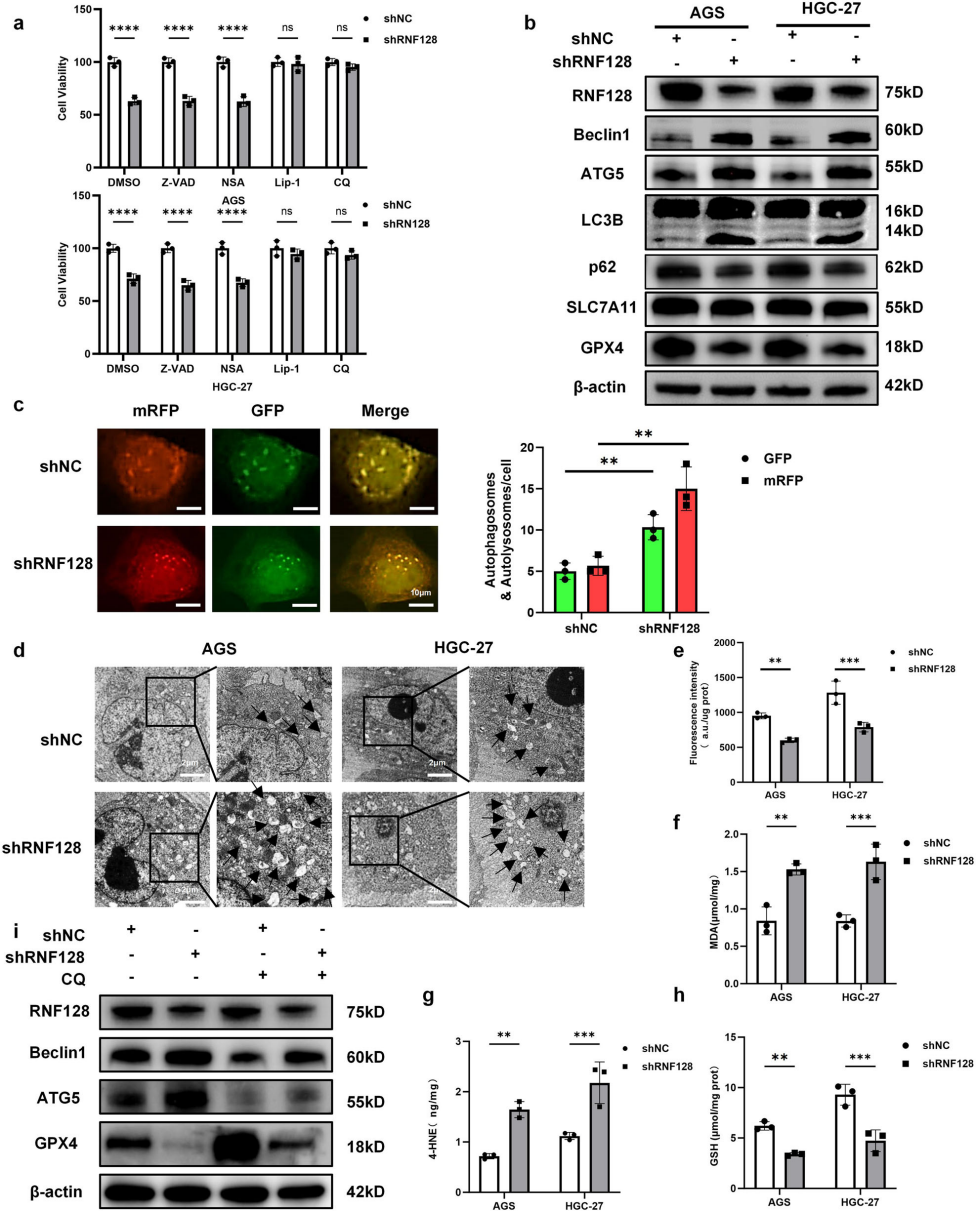
RNF128 knockdown promotes autophagy-dependent iron death in GC cells. **a** 48 h after plasmid transfection, different cell death specific inhibitors 10 μM (Z-VAD-FMK (Z-VAD), 0.5 μM NECROSULFONAMIDE (NSA), 1 μM Liproxstatin-1and 20 μM Chloroquine (CQ)) as well as DMSO were added and treated for 8 h. Cell viability was measured by CCK-8. **b** The expression content of autophagy and iron death-related proteins was detected after knockdown of RNF128. **c** The number of autophagosomes and autophagolysosomes after transfection by mRFP-GFP-LC3 adenovirus was observed by confocal microscopy upon knockdown of RNF128. **d** Number of autophagosomes and autophagolysosomes observed by transmission electron microscopy upon knockdown of RNF128. **e**–**h** Detection of cystine uptake capacity, MDA, 4-HNE and GSH expression in GC cells upon knockdown of RNF128. (i) Autophagy and iron death-related proteins were detected after knockdown of RNF128 and addition of specific autophagy inhibitor CQ. (*n* = 3, independent experiments), ****p < 0.0001, ***p < 0.001, **p < 0.01, ns not significant.

In this section, we explore the mechanistic pathway of RNF128 in detail. Previously we assessed cell death by detecting cell viability by CCK-8 assay (Fig. 2a) and showed that RNF128 knockdown increased cell death in GC cells. We then further explored the exact type of GC cell death (e.g. apoptosis, necrosis, autophagy and ferroptosis) induced by RNF128 knockdown using different specific inhibitors. RNF128-induced cell death was reversed after treatment with the autophagy inhibitor chloroquine (CQ) and the inhibitor of ferroptosis, Lipoxatin-1 (Lip-1), but not the apoptosis inhibitor Z-VAD-FMK (Z-VAD), and the necrosis inhibitor Necrosulfonamide (NSA), as compared to controls (Fig. 3a). These results suggest that the cell death induced by RNF128 knockdown may be autophagy or ferroptosis. Therefore, we examined the indices associated with autophagy and ferroptosis in subsequent experiments. We found that after knocking down RNF128, the proportion of autophagy-related proteins, such as Beclin1, autophagy protein 5 (ATG5), and light chain 3 β (LC3B) II/I, significantly increased, while Sequestosome-1 (p62) significantly decreased (Fig. 3b), and vice versa (Fig. S4a). In addition, when RNF128 knockdown and the simultaneous transfection of LC3 fluorescent double-labeled lentivirus were performed, we observed the tandem fluorescent-tagged LC3 (mRFP-GFP-LC3) fusion protein and found that the green fluorescence in the HGC-27 cells was quenched from the center to the periphery, which made the LC3 fluorescent particles appear red. However, the green fluorescence of the control group did not undergo quenching, and the LC3 fluorescent particles appeared yellow (Fig. 3c). When RNF128 was overexpressed, the LC3 fluorescent particles in the control group remained yellow. However, the number of fluorescent particles in the experimental group was significantly reduced (Fig. S4b).

Transmission electron microscopy experiments also showed that autophagosomes and autophagolysosomes were significantly increased in GC cells after RNF128 knockdown (Fig. 3d), whereas autophagosomes and autophagolysosomes were significantly reduced after RNF128 overexpression (Fig. S4c). These experimental results suggested that RNF128 knockdown significantly promoted autophagy in GC cells, whereas RNF128 overexpression inhibited autophagy. In addition, there was no significant change in SLC7A11 expression after RNF128 knockdown, whereas the level of GPX4 was significantly reduced (Fig. 3b). Simultaneously, the cellular uptake of cystine was significantly reduced, the intracellular production of the lipid peroxides Malondialdehyde (MDA) and 4-hydroxynonenal (4-HNE) was significantly increased, and GSH was significantly reduced (Fig. 3e–h). A contradictory trend was observed following RNF128 overexpression (Fig. S4d–g). It is well known that the main function of SLC7A11 is to mediate the transport of cystine and cysteine and to maintain the production of intracellular antioxidant-glutathione [29, 37, 38]. Although knockdown of RNF128 did not significantly alter the protein expression of CSL7A11, the significant decrease in intracellular cystine uptake and the increase in lipid peroxidation suggest that a significant decrease in the function of our SLC7A11 occurred and ultimately increased the level of iron metabolism in GC cells. To further investigate the relationship between RNF128-induced autophagy and ferroptosis, we examined the levels of autophagy and ferroptosis in HGC-27 cells after adding the autophagy inhibitor chloroquine, which showed that Beclin1 and ATG5 were markedly reduced, GPX4 was markedly elevated (Fig. 3i), MDA and 4-HEN were significantly decreased, and GSH was markedly elevated (Fig. S5a–d). Therefore, autophagy and ferroptosis are inhibited by adding autophagy inhibitors. Ferroptosis induced by RNF128 knockdown is dependent on autophagy. These data suggest that RNF128 knockdown promotes autophagy-dependent ferroptosis in GC cells.

### RNF128 interacts with Beclin1 through the PA domain

**Fig. 4:**
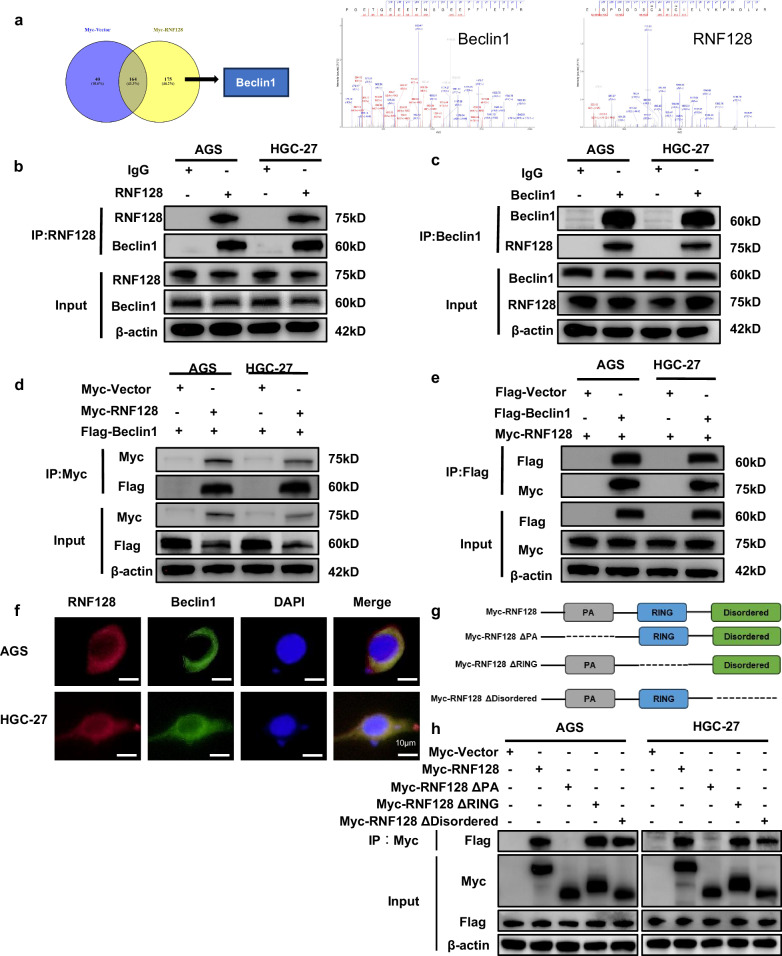
RNF128 and Beclin1 interaction. **a** LC–MS/MS-based mass spectrometry secondary spectra of RNF128 and Beclin1. **b**, **c** Western blot detection of endogenous protein immunoprecipitation between RNF128 and Beclin1 in GC cells. **d**, **e** Western blot detection of exogenous protein immunoprecipitation between RNF128 and Beclin1 in GC cells. **f** Immunofluorescence detection of fluorescence co-localisation of RNF128 and Beclin1 in GC cells. **f** Schematic representation of RNF128 wild type and truncated mutant. **g**, **h** Co-IP detection of RNF128 truncated mutant interaction with Beclin1 48 h after plasmid transfection. (*n* = 3, independent experiments).

In this section, we explored the downstream targets of RNF128 and transfected HEK 293-T cells with a control plasmid and RNF128 overexpression. Liquid chromatography-mass spectrometry (LC-MS)/MS experiments were performed to explore the potential molecular targets of RNF128.And of these 175 proteins, we screened the top 5 of them through a large body of literature and found Beclin1, a classical protein associated with autophagy (Fig. 4a). We performed endogenous and exogenous Co-immunoprecipitation (Co-IP) validation in GC cells to further validate the interaction between RNF128 and Beclin1. Figure 4b–e shows that RNF128 interacted with Beclin1. Immunofluorescence also confirmed that RNF128 and Beclin1 were co-localized in GC cells (Fig. 4f). Furthermore, we explored the molecular structure of RNF128 interacting with Beclin1. As shown in the protein structure analysis (UniProt: https://www.UniProt.org/), RNF128 consists of three structural domains, including PA (amino acid [AA] sequence 75–183), RING (amino acid [AA] sequence 277–318), and Disordered (AA sequence (amino acid [AA] sequence 346–428). We constructed three truncated plasmids, named RNF128ΔPA, RNF128ΔRING, and RNF128ΔDisordered, based on the wild-type (WT) plasmid with the complete RNF128 sequence and the above structural domain sequences (Fig. 4g). The GC cells were then transfected with these plasmids. Co-IP results showed that RNF128ΔRING and RNF12ΔDisordered mutants could still interact with Beclin1, whereas the RNF128ΔPA mutant lost the ability to bind to Beclin1 (Fig. 4h). These findings suggest that RNF128 interacts with Beclin1 through the PA domain.

### RNF128 degrades Beclin1 by ubiquitination

**Fig. 5:**
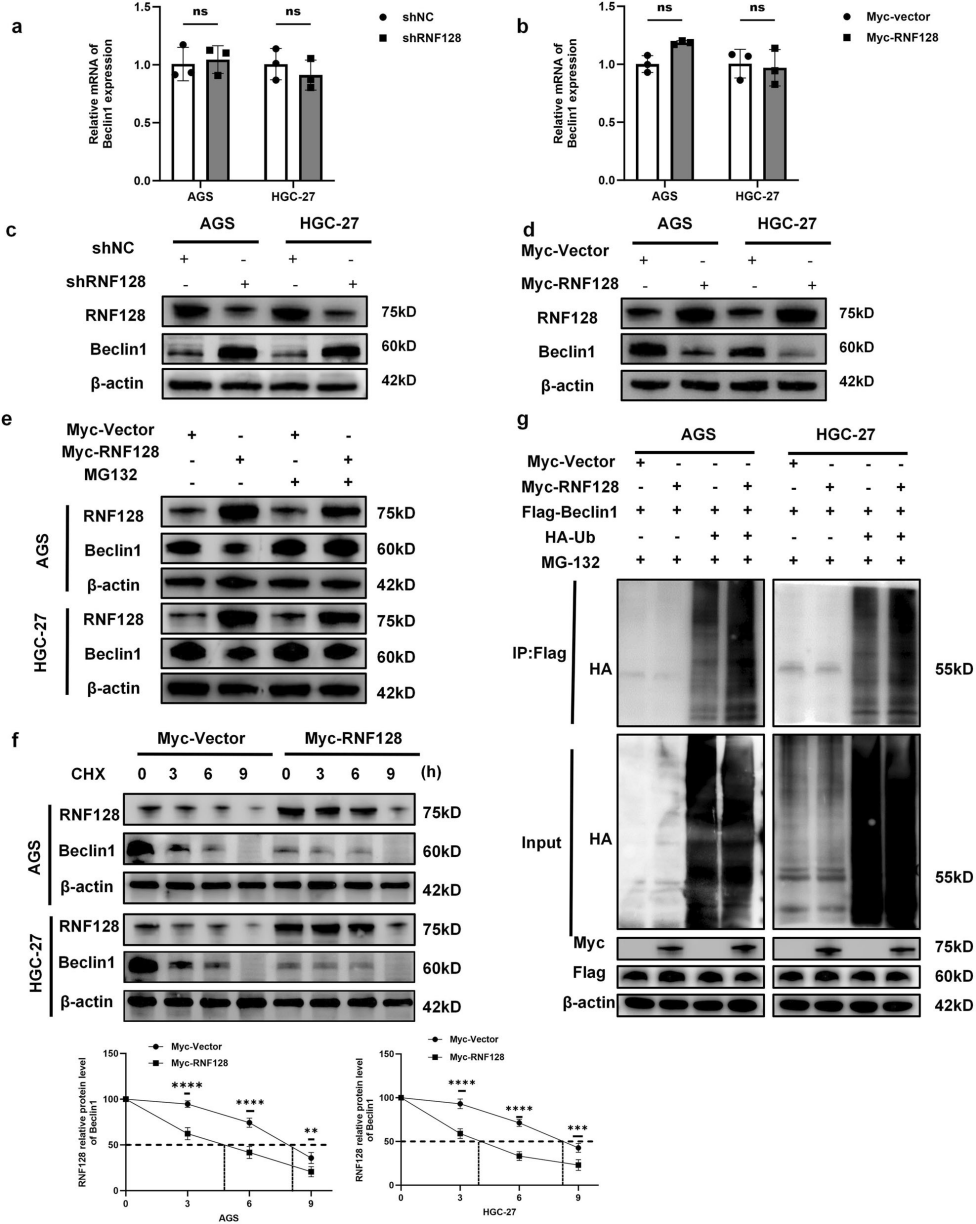
RNF128 ubiquitinates degradation of Beclin1. **a**, **b** Q-PCR to detect the mRNA content of Beclin1 in RNF128 knockdown or overexpression. **c**, **d** Western blot detection of protein content of Beclin1 upon RNF128 knockdown or overexpression. **e** Protein content of Beclin1 upon knockdown of RNF128 with or without 10 μM MG132 detection. **f** Cells were treated with 100 μg/ml CHX when overexpressing RNF128 and the protein content of Beclin1 was detected after 0, 3, 6, and 9 h. **g** The ubiquitination level of Beclin1 was detected in GC cells co-transfected with Myc-RNF128 plasmid, Flag-Beclin1 plasmid and HA-ub plasmid. (*n* = 3, independent experiments), ****p < 0.0001, ***p < 0.001, **p < 0.01, ns not significant.

We further explored how RNF128 regulates Beclin1 expression. We found that RNF128 knockdown and overexpression did not change the mRNA content of Beclin1 (Fig. 5a, b). However, Western blot analysis showed that the protein level of Beclin1 was significantly upregulated after RNF128 knockdown, and the protein level of Beclin1 was significantly downregulated after RNF128 overexpression (Fig. 5c, d). In addition, Beclin1 knockdown or overexpression did not affect the mRNA and protein expression of RNF128 (Fig. S6a–d). Therefore, we hypothesized that RNF128 might regulate Beclin1 through ubiquitination posttranslational modifications. Thus, to further investigate the mechanism of Beclin1 degradation, we treated GC cells with the specific proteasome inhibitor MG132, and the results showed that elevated Beclin1 levels induced by RNF128 knockdown were reversed by MG132 treatment (Fig. 5e). Furthermore, after cycloheximide (CHX) blocked ab initio protein synthesis in RNF128 overexpressing cells, RNF128 significantly promoted the downregulation of Beclin1 (Fig. 5f). Subsequently, we extracted total proteins from GC cells transfected with the ubiquitous plasmid ub and MG132 treatment. In addition, Western blot analysis showed that RNF128 overexpression significantly increased the ubiquitination of Beclin1 (Fig. 5g). These studies showed that RNF128 inhibits the stability of Beclin1 protein through the ubiquitin-proteasome pathway.

### RNF128 knockdown induces autophagy-dependent ferroptosis by enhancing the stability of Beclin1, which inhibits System Xc activity

**Fig. 6:**
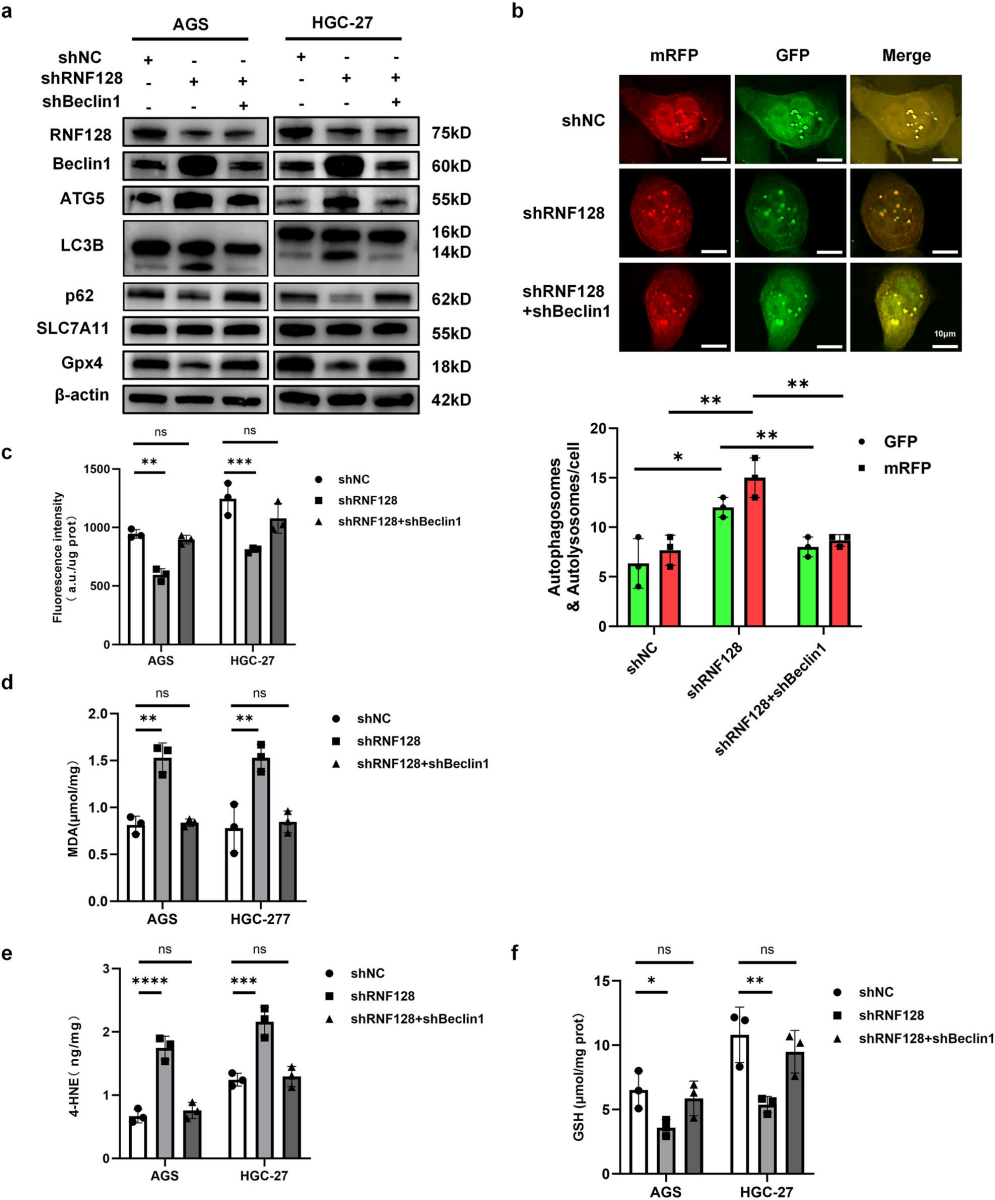
Knockdown of RNF128 induces autophagy-dependent iron death in cells via activation of Beclin1/SLC7A11/GPX4 axis. **a** The expression levels of autophagy and iron death-related proteins in GC cells were detected by Western blot when RNF128 and Beclin1 were knocked down simultaneously. **b** When RNF128 and Beclin1 were knocked down at the same time, the numbers of autophagosomes and autophagolysosomes were observed by confocal microscopy after transfection with mRFP-GFP-LC3 adenovirus. **c**–**f** Cystine uptake capacity, MDA, 4-HNE, and GSH content in GC cells were detected when knockdown of RNF128 and simultaneous knockdown of Beclin1. (*n* = 3, independent experiments), ***p < 0.001, **p < 0.01, *p < 0.05, ns not significant.

Increasing evidence suggests that Beclin1 is crucial in autophagy-dependent ferroptosis [34]. Based on these studies, we hypothesized that RNF128 may affect the biological behavior of GC by regulating the stability of Beclin1 and, thus, autophagy-dependent ferroptosis. We examined autophagy- and ferroptosis-related indices (Beclin1, ATG5, p62, LC3BII, SLC7A11, and GPX4) upon RNF128 knockdown. The proliferation and migration of GC cells were inhibited, and the levels of autophagy and ferroptosis increased after RNF128 knockdown. However, we found that the promotion of autophagy and ferroptosis as well as the inhibition of proliferation brought about by knockdown of RNF128 was reversed in the presence of concomitant knockdown of Beclin1 (Figs. 6a–f and S7a, c) and vice versa (Figs. S6e–j and S7b, d). This suggests that RNF128 knockdown activates autophagy-dependent ferroptosis and ultimately inhibits GC progression by promoting the Beclin1/SLC7A11/GPX4 axis.

### Knockdown of RNF128 significantly inhibited the growth of xenograft tumors in nude mice

**Fig. 7:**
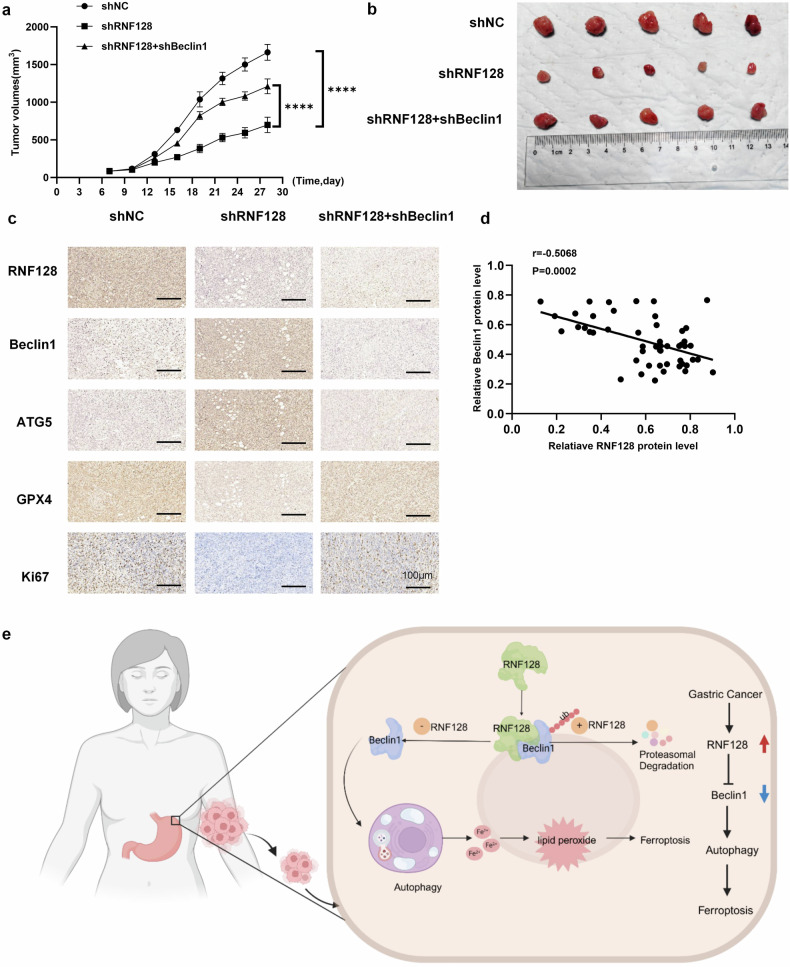
Knockdown of RNF128 significantly inhibited the growth of xenograft tumors in nude mice. **a** Tumor volume of each group from day 7 to day 28 after tumor implantation. **b** Macroscopic images of tumors in each group on day 28 after tumor implantation. **c** Representative photographs of IHC of RNF128, Beclin1, ATG5, GPX4 and Ki67 in each group. **d** Correlation analysis of RNF128 and Beclin1 expression in GC tissues. **e** Diagram of the mechanism by which RNF128 promotes GC proliferation through ubiquitination degradation of Beclin1. (*n* = 3, independent experiments), ****p < 0.0001.

Meanwhile, we further investigated the role of RNF128 in vivo through subcutaneous tumor formation in nude mice. The tumor growth curves showed that the tumor growth rate was significantly reduced from day 7 to day 28 in the shRNF128 group compared with the control group. However, there was no statistically significant difference between the shRNF128+shBeclin1 and control groups (Fig. 7a). In addition, a significant reduction in tumor volume and weight was observed in the shRNF128 group compared with the other two groups (Figs. 7b and S8a, b). IHC staining confirmed that Beclin1 and ATG5 expression levels were significantly elevated in the RNF128 knockdown group. The expression levels of GXP4 and Ki67 were significantly reduced (Fig. 7c). We then examined the level of Beclin1 in 50 clinical GC samples, and correlation analysis showed that RNF128 was negatively correlated with Beclin1 (Fig. 7d). These data are consistent with the in vitro experimental data, further validating that RNF128 promotes the malignant progression of GC by inhibiting Beclin1 stability.

## Discussion

As a RING-type E3 ubiquitin ligase, RNF128 is lowly expressed in some malignant tumors and promotes cancer cell proliferation, such as melanoma and prostate cancer [17, 18]. However, in other gastrointestinal (GI) tumors that are more closely associated with GC, such as hepatocellular carcinoma and colorectal cancer, RNF128 is highly expressed, promoting tumor progression [19, 20]. In this study, we demonstrated for the first time that RNF128 expression in GC tissues and cell lines is higher than that in matched adjacent tissues or GES-1 cells. We also demonstrated that RNF128 knockdown promoted programmed cell death in GC cells by establishing a cellular model of RNF128 knockdown. Therefore, to further investigate the role of RNF128 in GC cells and the possible underlying mechanisms, we performed LC-MS/MS to determine the interactions between RNF128 and Beclin1. This was verified using Co-IP and immunofluorescence co-localization experiments. Beclin1 mRNA and protein levels were examined in the presence of interfering RNF128 expression. Notably, RNF128 downregulation significantly increased the protein level of Beclin1 but did not change its mRNA level. Considering that RNF128 is a member of the E3 ubiquitin ligase family, we suggest that post-translational modifications may be crucial in regulating the protein stability of Beclin1. Finally, we demonstrated that RNF128 directly binds and induces polyubiquitination of Beclin1 through its N-terminal PA domain.

Ferroptosis is a recently discovered regulatory cell death mechanism [39–42]. Notably, several recent studies have demonstrated a close relationship between ferroptosis and autophagy. Autophagy is an upstream pathway that induces ferroptosis by regulating cellular iron homeostasis and lipid peroxide production [43–46]. In this study, we found that RNF128 knockdown increased autophagic flux and autophagosome production, which may be a potential mechanism through which RNF128 inhibits ferroptosis and promotes tumor progression. Beclin1, as a key molecule in autophagy regulation, is critical in several physiological and pathological conditions [47, 48]. Rong et al. demonstrated that Ubiquitin Specific Peptidase 11 was induced by deubiquitinating Beclin1 autophagy-dependent ferroptosis in patients with spinal cord ischemia-reperfusion injury [34]. Xu et al. also reported that RNF216 regulates autophagy by targeting the ubiquitination of Beclin1 and is critical in innate immune responses during bacterial and viral infections [49]. Consistent with previous studies, we found that Beclin1 controls intracellular cystine uptake in GC cells by regulating SLC7A11 activity and ultimately promotes ferroptosis [35]. Subsequently, we found that inhibiting autophagy using chloroquine partially blocked RNF128 knockdown-induced ferroptosis, thus confirming that RNF128 knockdown-induced inhibition of proliferation is associated with autophagy-dependent ferroptosis.

This study has some limitations. We verified that RNF128 induced the degradation of Beclin1 and subsequent alterations in SLC7A11 and GPX4; however, we failed to fully validate the effect of RNF128 on patient survival at the clinical tissue level and the relationship between factors such as age, sex, TNM stage, tumor metastasis, and the amount of RNF128. We aim to discuss these in our subsequent studies. However, this did not hinder the accuracy or rigor of our results.

In conclusion, our findings highlighted the importance of RNF128-mediated post-translational modifications of Beclin1 in GC progression. The interaction between these two proteins is a crucial molecular event that triggers autophagy, promotes intracellular lipid peroxide accumulation, and induces ferroptosis (Fig. 7e). Therefore, an in-depth understanding of the molecular mechanisms and signaling pathways involved in autophagy-dependent ferroptosis may provide new diagnostic and therapeutic approaches for GCs.

## Material and methods

### Clinical samples

This study enrolled 50 treatment-naïve patients with primary GC who underwent radical gastrectomy at the General Surgery Center of The First Affiliated Hospital of Shandong First Medical University (Jinan, China) between October 2022 and October 2024. Inclusion criteria comprised: (1) histopathological confirmation of GC through both preoperative and postoperative examinations, (2) completion of curative-intent radical resection surgery, and (3) availability of complete clinicopathological records. Written informed consent was obtained from all participants prior to their inclusion in the study.

### Cell culture

The GES-1, AGS, HGC-27, and HEK 293T cells were purchased from Procell Life Sciences and Technology (Wuhan, China). The cells were cultured in Roswell Park Memorial Institute medium (RPMI 1640), Ham’s F-12K (Kaighn’s) Medium, Minimum Essential Medium, and Dulbecco’s Modified Eagle’s Medium supplemented with 10% fetal bovine serum (Thermo Fisher, CA, USA) and 1% penicillin/streptomycin (Thermo Fisher). Of note, all cells were cultured in a humidified incubator at 37 °C, 5% CO_2_, identified by STR and were mycoplasma-free.

### Cell Counting Kit-8 and colony formation assays

Following the manufacturer’s instructions, CCK-8 assay was performed on GC cells using the kit (Nihon Kohden, Tokyo, Japan). Finally, cell viability was measured at 450 nm. For the colony formation assay, GC cells were incubated in 6-well plates, cultured for 14 days, and fixed with 4% paraformaldehyde for 30 min. Furthermore, the cells were stained with 1% crystal violet (Solarbio, Beijing, China) for 15 min, and the number of colonies with diameters > 50 μm was recorded.

### Quantitative real-time fluorescence PCR analysis(Q-PCR)

Total mRNA was extracted from GC cells and tissues using an RNeasy Mini Kit (Qiagen, Hilden, Germany). Complementary DNA (cDNA) was synthesized by reverse transcription using a PrimeScript RT kit (Takara, Dalian, China). cDNA was extracted from GC cells and tissues using SYBR Green, RNF128 (5′- GTACGCATCTTAACGTGCAACC-3′;5′-CGGTCTCGCTGCGATTATC-3′) and Beclin1 (5′-CCATGCAGGTGAGCTTCGT-3′;5′-GAATCTGCGAGAGACACCATC-3′) specific primers were used for cDNA amplification. β-actin was used as an internal standard, and the experimental results were analyzed using the 2(- ΔΔ C(T) statistical method.

### Western blot analysis

Total proteins were extracted from GC tissues or cultured GC cells, washed twice with 1 × PBS, and then lysed by RIPA on ice for 30 min. Protein samples (30 μg) were separated electrophoretically on a sodium dodecyl sulfate-polyacrylamide gel electrophoresis gel, and the gel was transferred to a polyvinylidene fluoride (PVDF) membrane. The transferred PVDF membrane was then closed in 5% bismuth sulfite agar (BSA) for 1 h and incubated overnight at 4 °C with the corresponding primary antibodies, including RNF128 (abcam, ab137088 and ab72533, 1:1000, CA, USA), Beclin1 (CST, #3495, 1:1000), ATG5 (CST, #12994, 1:1000), LC3B (CST, #83506, 1:1000), p62 (CST, #5114, 1:1000), SLC7A11 (Proteintech, 26864-1-AP, 1:1000, MA, USA), GPX4 (Proteintech, 67763-1-Ig, 1:1000), Flag (Proteintech, 66008-4- Ig, 1:1000), Myc (Proteintech, 16286-1-AP, 1:1000), HA (Proteintech, 81290-1-RR, 1:1000), β-actin (Proteintech, 51067-2-AP, 1:5000). Finally, the membranes were incubated with the corresponding secondary antibodies for 1 h to obtain images. Additionally, full and uncropped western blots have been uploaded as ‘Supplemental Material’.

### MDA and 4-HNE detection

The MDA concentration in cell lysates was detected using the Lipid Peroxide (MDA) Assay Kit (S0131M, Beyotime, China) described in the manual. According to the manufacturer’s instructions, the 4-HNE concentration was determined using an Enzyme-linked immunosorbent assay kit (MBS161454; MyBioSource, San Diego, CA, USA).

### Double-labeled adenovirus mRFP-GFP-LC3 transfection

GC cells were inoculated into cell crawls, wall-applied, and transfected with mRFP-GFP-LC3 adenovirus (Hanbio, China) following the manufacturer’s instructions. Furthermore, the GC cells were treated with phosphate buffer, fixed in 4% paraformaldehyde, and the nuclei were stained with 4′,6-diamidino-2-phenylindole (DAPI). The cells were observed under a confocal microscope.

### Transmission electron microscopy

GC cells were treated with pre-cooled 2% glutaraldehyde solution for 2 h at 4 °C to fix the cell mass. Subsequently, they were stained with 2% uranyl acetate solution for 2 h and then dehydrated with 50%, 70%, 90 and 100% acetone. The cells were embedded and ultrathin sections were prepared, which could be observed under an electron microscope (FEI Tecnai, Hillsboro, OR, USA).

### Immunofluorescence assay

GC cells were inoculated in cell crawls, and after walling, GC cells were treated with 4% polymethanol and permeabilized with 0.2% Triton X-100 for 10 min. Furthermore, they were closed with 5% BSA solution for 1 h, followed by overnight incubation with primary antibodies [Flag (66008-4-Ig, 1:500), Myc (16286-1-AP, 1:500)] at 4 °C. After washing thrice with phosphate-buffered saline, the cells were incubated with the corresponding secondary antibodies for 1 h, and the nuclei were stained with DAPI. The cells were observed under a confocal microscope.

### In vitro ubiquitination assay

For the in vitro ubiquitination assay, HA-ub plasmids with or without Myc-RNF128 and Flag-Beclin1 were transfected using the Lipofectamine 2000 transfection reagent. After 48 h, the total cellular proteins were extracted, and IP assayed using agarose beads coupled with anti-Myc or anti-FLAG antibodies. Protein blotting analysis was performed after washing thrice with the IP buffer.

### Establishment of subcutaneous graft tumor model

Four-week-old BALB/c nude mice were purchased from Vital River (Beijing, China) for subcutaneous transplantation tumor modeling. After 7 days of feeding in the specific-pathogen-free environment, the mice were randomly divided into three groups: shNC (*n* = 5), shRNF128 (*n* = 5), and shRNF128+ shBeclin1 (*n* = 5). Lentivirally transfected shNC, shRNF128 and shRNF128+shBeclin1 stably expressing HGC-27 cells were screened using puromycin, and 1 × 10^5^ GC cells mixed with matrix gel at a 1:1 ratio were injected subcutaneously into the ipsilateral inguinal region of 15 nude mice. The tumor size was measured every 3 days from day 7, and all mice were sacrificed on day 28; the tumors were then removed and weighed for volume and mass.

### Immunohistochemistry

Firstly, the surgically removed tumor samples were observed visually, and areas with darker colour, harder texture and poorly defined borders were selected for making and sectioning of tissue wax blocks. Paraffin sections were baked in an oven at 65 °C for 1–2 h, then deparaffinised in xylene and hydrated in various concentrations of alcohol. Heat-activated antigen repair was performed using sodium citrate buffer (10 mmol/litre, pH 6.0). A drop of 3% hydrogen peroxide was added to the sections and incubated at 37 °C for 30 min, followed by blocking with 5% BSA for 30 min and overnight incubation with the corresponding antibody. The next day, the sections are incubated with the appropriate secondary antibody for 30 min. After staining with diaminobenzidine and hematoxylin, sections are prepared for microscopic observation.

### Statistical analysis

Experimental data are presented as the mean ± standard deviation (SD) from at least three independent replicates. For continuous endpoints, comparisons between two groups were performed using Student’s t-test, while one-way analysis of variance (ANOVA) was employed for multiple independent groups. Paired t-tests or repeated-measures one-way ANOVA models were applied for comparisons within paired groups. Time-course repeated measurements across multiple groups (e.g., tumor growth) were analyzed using two-way repeated-measures ANOVA. All statistical tests were two-tailed. Multiple comparison adjustments were performed using the Holm–Sidak method. Sample sizes were determined empirically and indicated in individual figure legends, rather than through formal power calculations based on pre-specified effect sizes. All in vitro experiments utilized biologically independent samples with ≥ 3 replicates, while in vivo experiments employed 6–16 mice per group. Data were assumed to follow a normal distribution without formal normality testing. Statistical significance was defined as *p* < 0.05.

## Supplementary information

Supporting-information

Full and uncropped western blots

Dataset 1

## Data Availability

All the data analysed and generated in this study are included in this manuscript. Additional data generated in this study are available upon request from the corresponding author.
