# Supplementary material for: RNF128 promotes gastric cancer progression by inhibiting autophagy-dependent ferroptosis through Beclin1 ubiquitination
Source: Cell Death Discov. 2025 Apr 19;11:187. doi: 10.1038/s41420-025-02488-8 (PMC12009371; doi:10.1038/s41420-025-02488-8)
Supplement: Supplementary file 1 — Supporting-information [file 41420_2025_2488_MOESM1_ESM.pdf]

Supplementary figure 1： Expression of RNF128 Iso1 in gastric cancer (a) Western blot detection of RNF128 Iso1 expression content in gastric cancer tissues and matched adjacent tissues. (a) Western blot assay to detect the expression content of RNF128 Iso1 in gastric epithelial cells GES-1 and GC cells AGS and HGC-27.

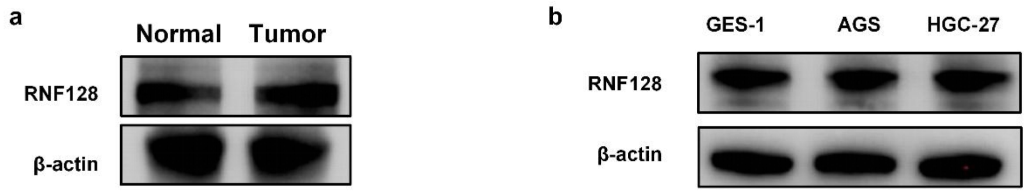

Supplementary figure 2： Validation of plasmid transfection efficiency (a-c) Q-PCR and Western blot experiments to validate the efficiency of RNF128 knockdown. (d-e) Q-PCR and Western blot experiments to verify the efficiency of RNF128 overexpression. ****$p < 0.0001$, ***$p < 0.001$, **$p < 0.01$.

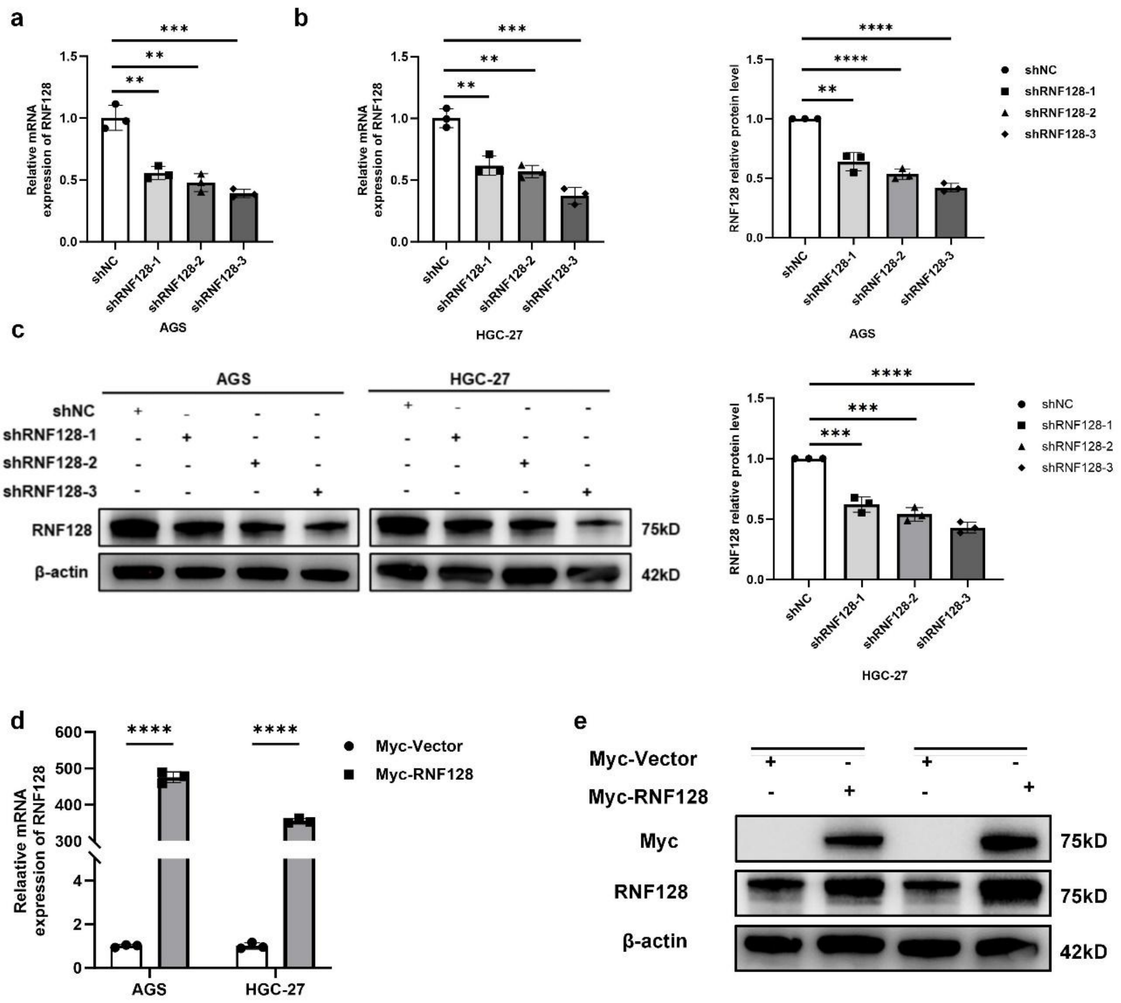

Supplementary figure 2

Supplementary figure 3： RNF128 overexpression promotes the proliferation and migration of GC cells. (a) CCK-8 assay to detect the cell viability of GC cells when RNF128 was overexpressed. (b) Colony formation assay to detect the proliferation ability of GC cells when RNF128 was overexpressed. (c-d) Transwell assay and cell scratch assay to detect the migration ability of GC cells when RNF128 was overexpressed. ****p < 0.0001, ***p < 0.001, *ns*: non-significant.

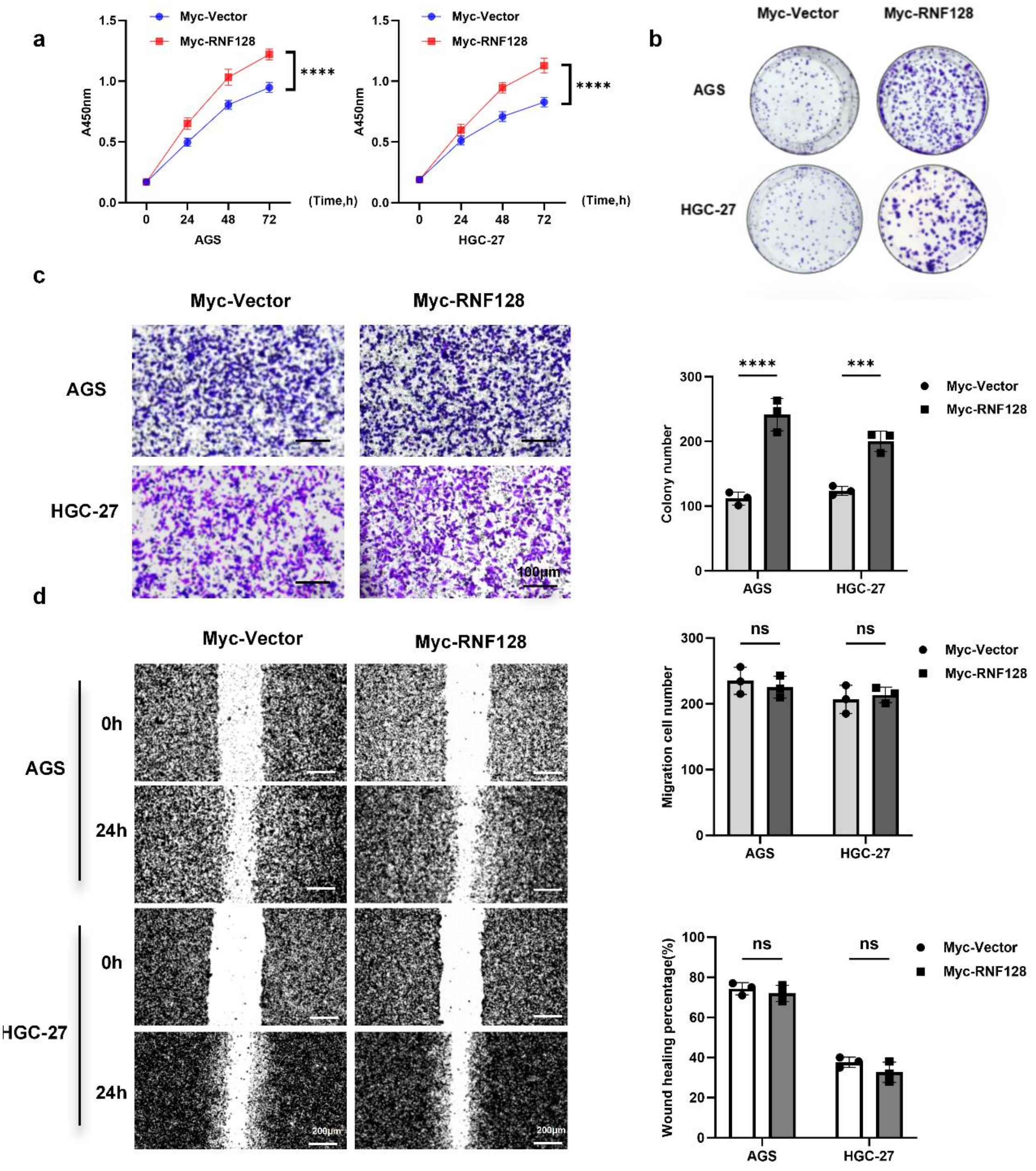

Supplementary figure 4： RNF128 overexpression inhibits autophagy-dependent iron death in GC cells. (a) The expression content of autophagy and iron death-related proteins was detected after overexpression of RNF128. (b) The number of autophagosomes and autophagolysosomes after transfection by mRFP-GFP-LC3 adenovirus was observed by confocal microscopy when overexpressing RNF128. (c) Number of autophagosomes and autophagolysosomes observed by transmission electron microscopy when overexpressing RNF128. (d-g) Cystine uptake capacity, MDA and, 4-HNE and GSH expression were detected in GC cells when overexpressing RNF128. ****p* < 0.001, ***p* < 0.01, **p* < 0.05.

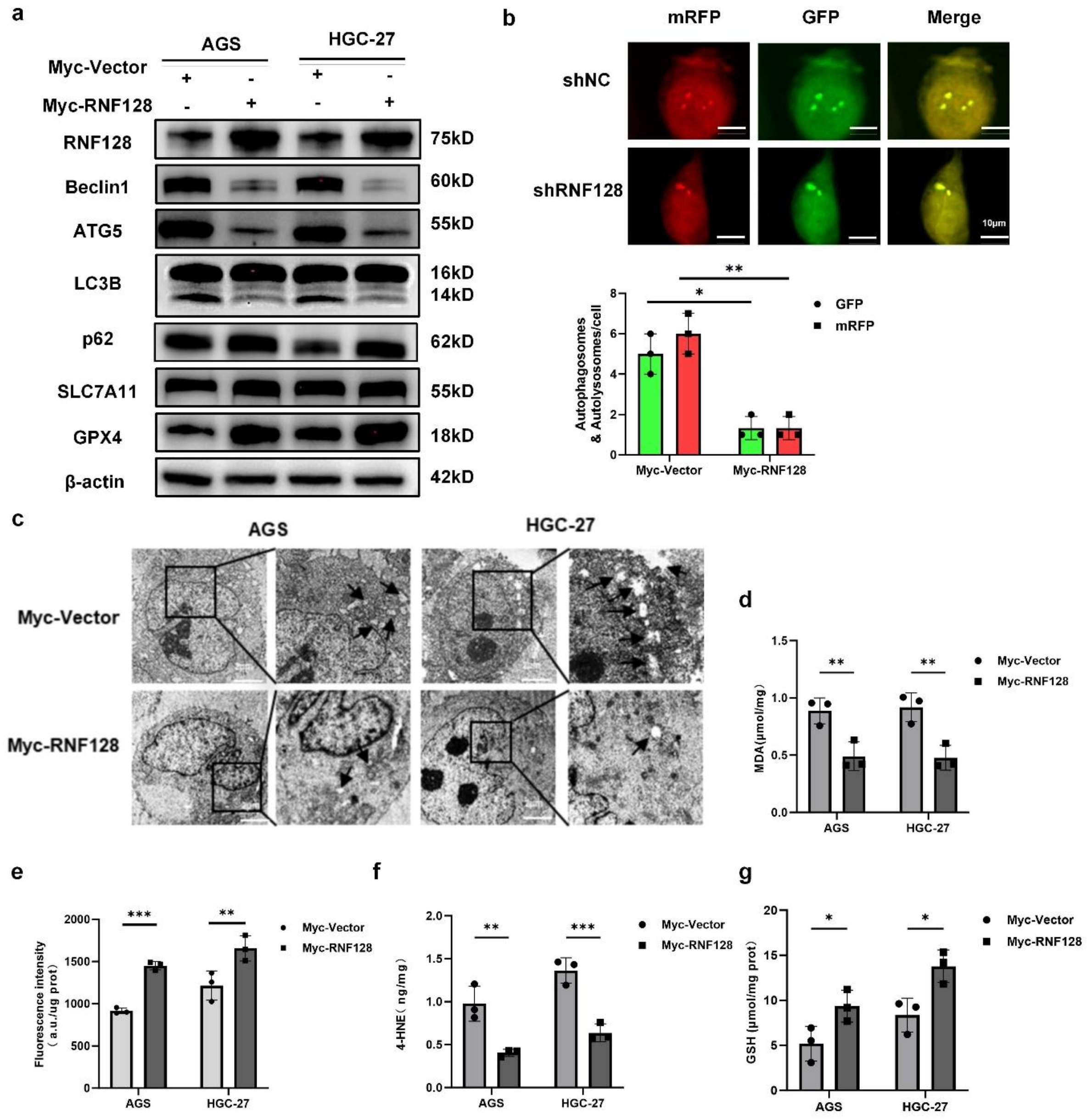

Supplementary figure 4

Supplementary figure 5： Knockdown of RNF128-induced ferroptosis is autophagy-dependent. (a-d) After knockdown of RNF128 and addition of the specific autophagy inhibitor CQ, cystine uptake capacity, MDA, 4HNE and GSH were detected in GC cells. ***$p < 0.001$, **$p < 0.01$.

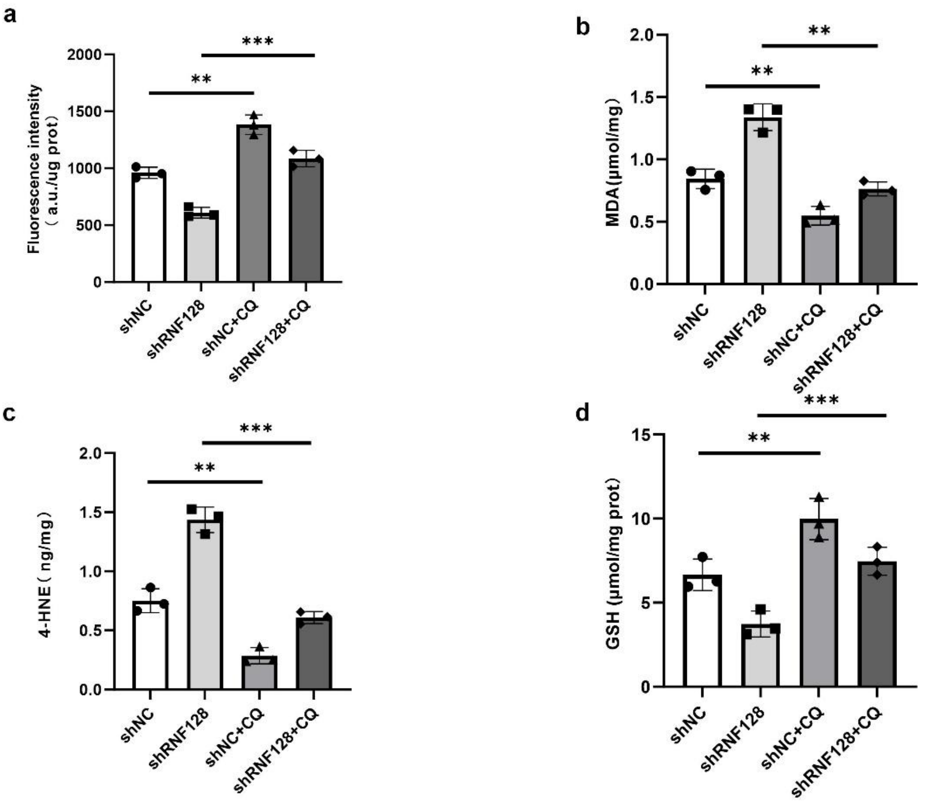

Supplementary figure 5

Supplementary figure 6: Overexpression of RNF128 inhibits autophagy-dependent iron death in cells by suppressing the Beclin1/SLC7A11/GPX4 axis. (a, b) Q-PCR to detect the mRNA content of RNF128 in Beclin1 knockdown or overexpression. (c, d) Western blot detection of protein content of RNF128 in Beclin1 knockdown or overexpression. (e) Western blot detection of autophagy and iron-death related proteins in GC cells when RNF128 and Beclin1 were overexpressed at the same time. (f) The number of autophagosomes and autophagolysosomes in HGC-27 cells transfected with mRFP-GFP-LC3 adenovirus was observed by confocal microscopy when RNF128 and Beclin1 were overexpressed at the same time. (g-j) Cystine uptake capacity, MDA, 4-HNE, and GSH content in GC cells were detected when knockdown of RNF128 and simultaneous knockdown of Beclin1. ****p* < 0.001, ***p* < 0.01, **p* < 0.05, ns: non-significant.

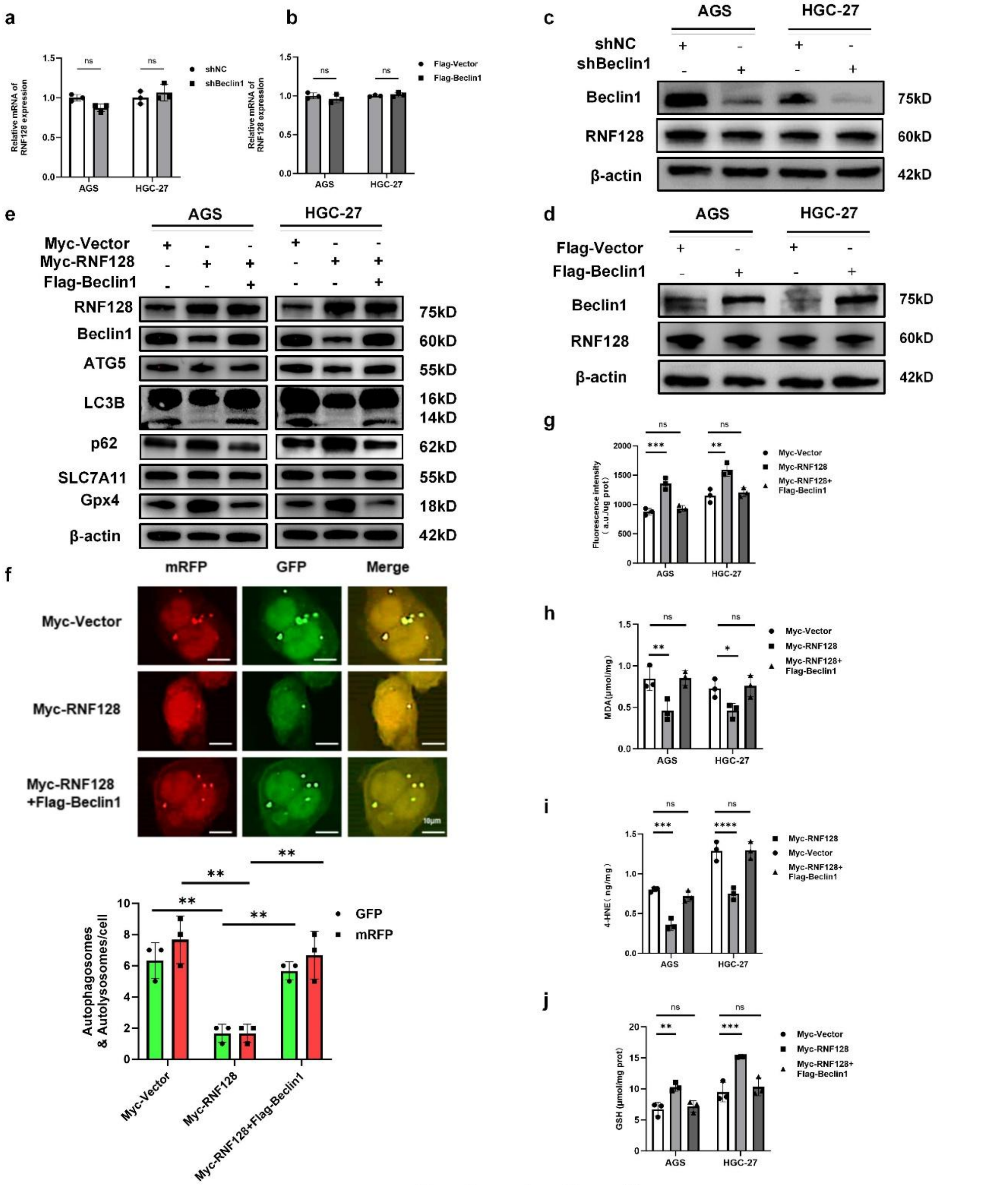

Supplementary figure 6

Supplementary figure 7： RNF128 affects the proliferative capacity of GC cells by acting on Beclin1 (a) Cell viability of GC cells was detected by CCK-8 assay when knockdown of RNF128 and Beclin1 was performed simultaneously. (b) When RNF128 and Beclin1 were overexpressed simultaneously, the viability of GC cells was detected by CCK-8. (c) Proliferation viability of GC cells was detected by colony formation assay when both RNF128 and Beclin1 were knocked down. (d) When RNF128 and Beclin1 were overexpressed simultaneously, the proliferation viability of GC cells was detected by colony formation assay. ****$p < 0.0001$, ns: non-significant.

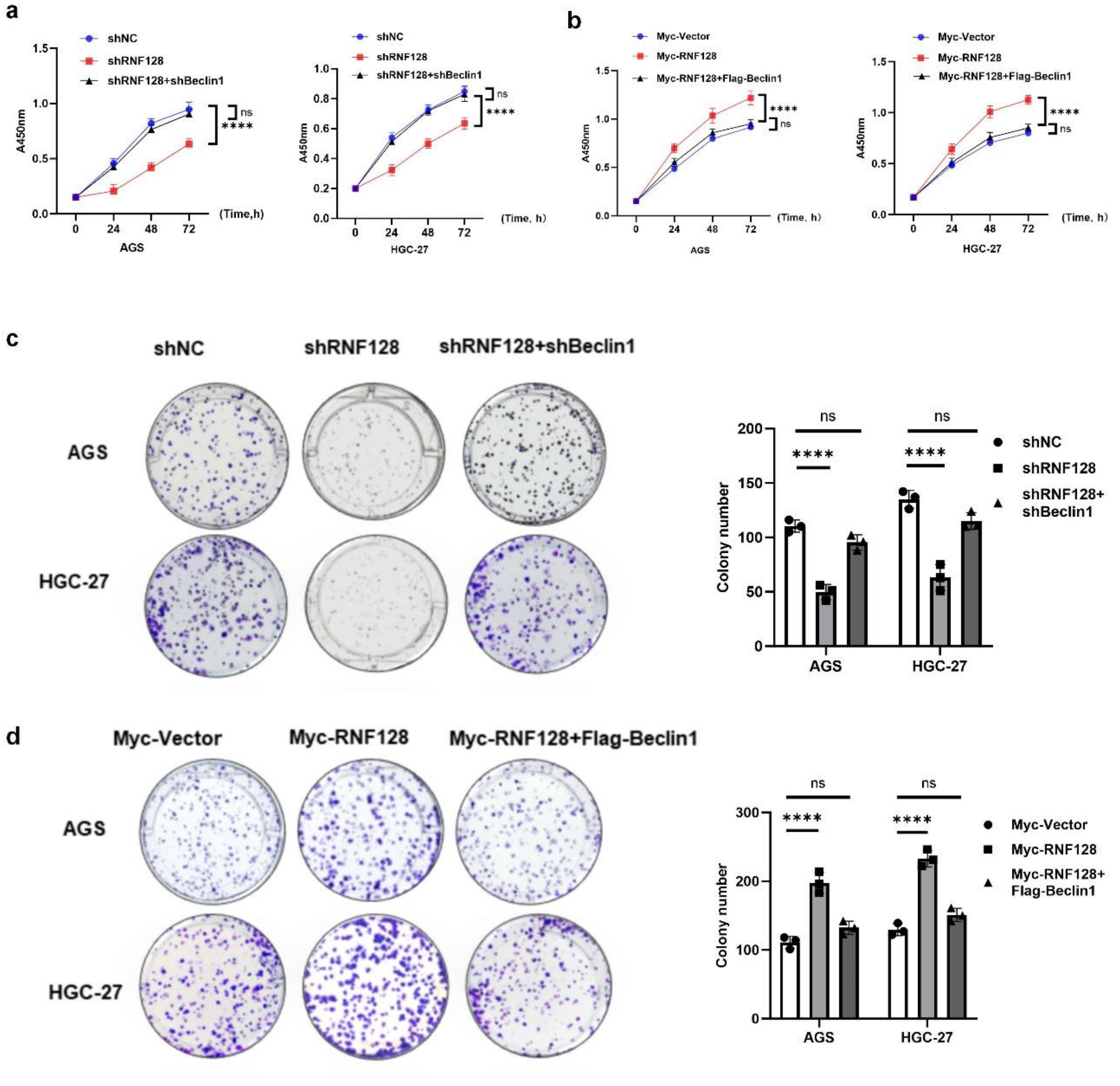

Supplementary figure 7

Supplementary figure 8: Knockdown of RNF128 effectively inhibited the growth of xenograft tumors in nude mice (a) Volume of the seeded tumors in each group on day 28 after seeding. (b) Mass of the seeded tumors in each group on day 28 after seeding. ****p < 0.0001.

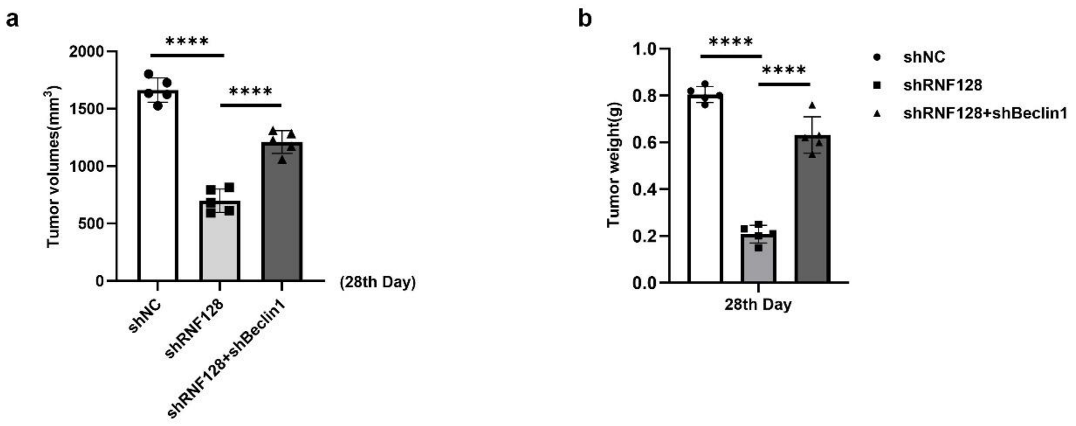

Supplementary figure 8
