# Supplementary material for: RNF128 promotes gastric cancer progression by inhibiting autophagy-dependent ferroptosis through Beclin1 ubiquitination
Source: Cell Death Discov. 2025 Apr 19;11:187. doi: 10.1038/s41420-025-02488-8 (PMC12009371; doi:10.1038/s41420-025-02488-8)
Supplement: Supplementary file 2 — Full and uncropped western blots [file 41420_2025_2488_MOESM2_ESM.pdf]

Full and uncropped western blots

Figure1 b

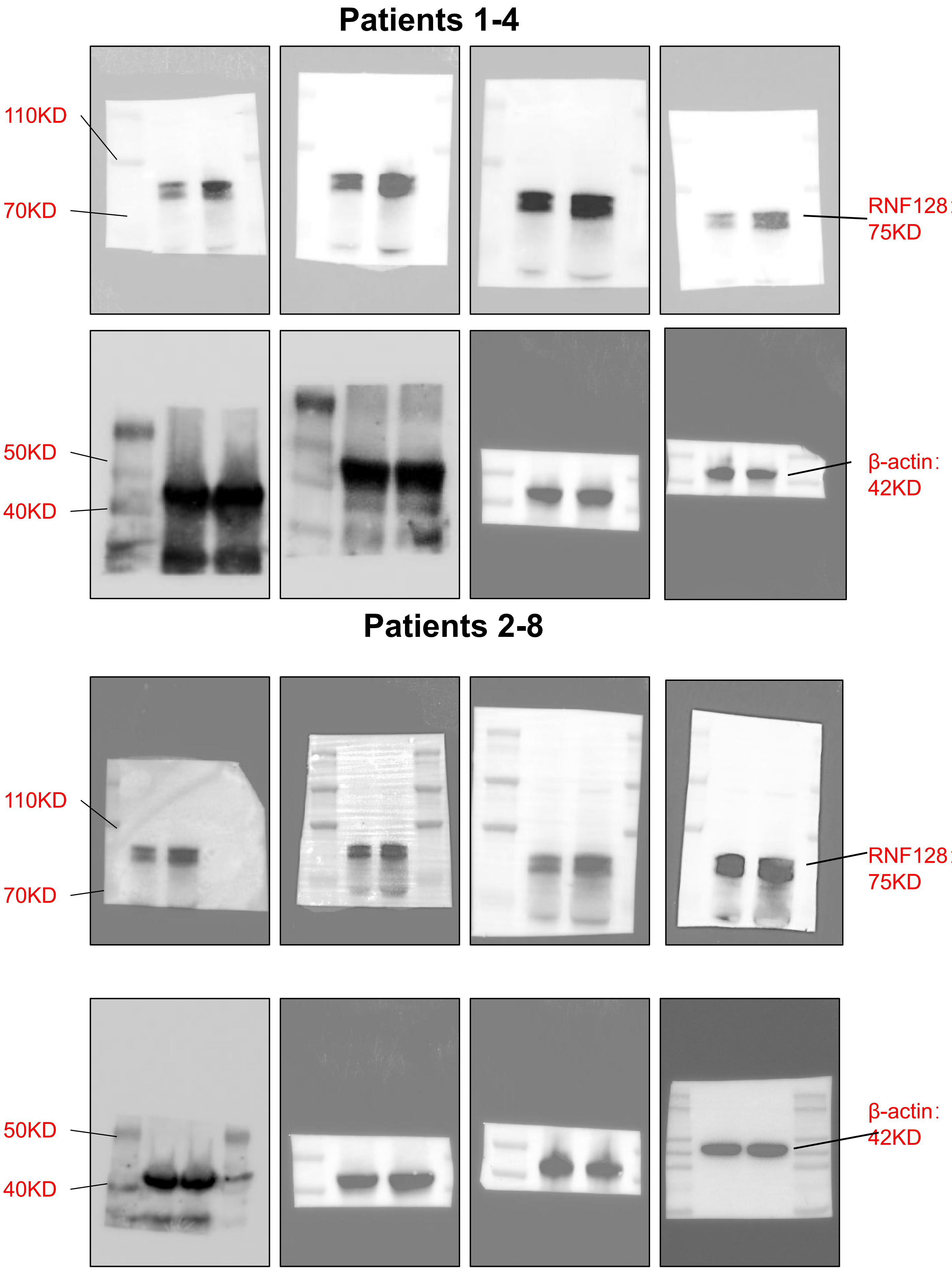
Patients 1-4
110KD
70KD
RNF128
75KD
50KD
40KD
β-actin:
42KD
Patients 2-8
110KD
70KD
RNF128
75KD
50KD
40KD
β-actin:
42KD

Patients 9-12

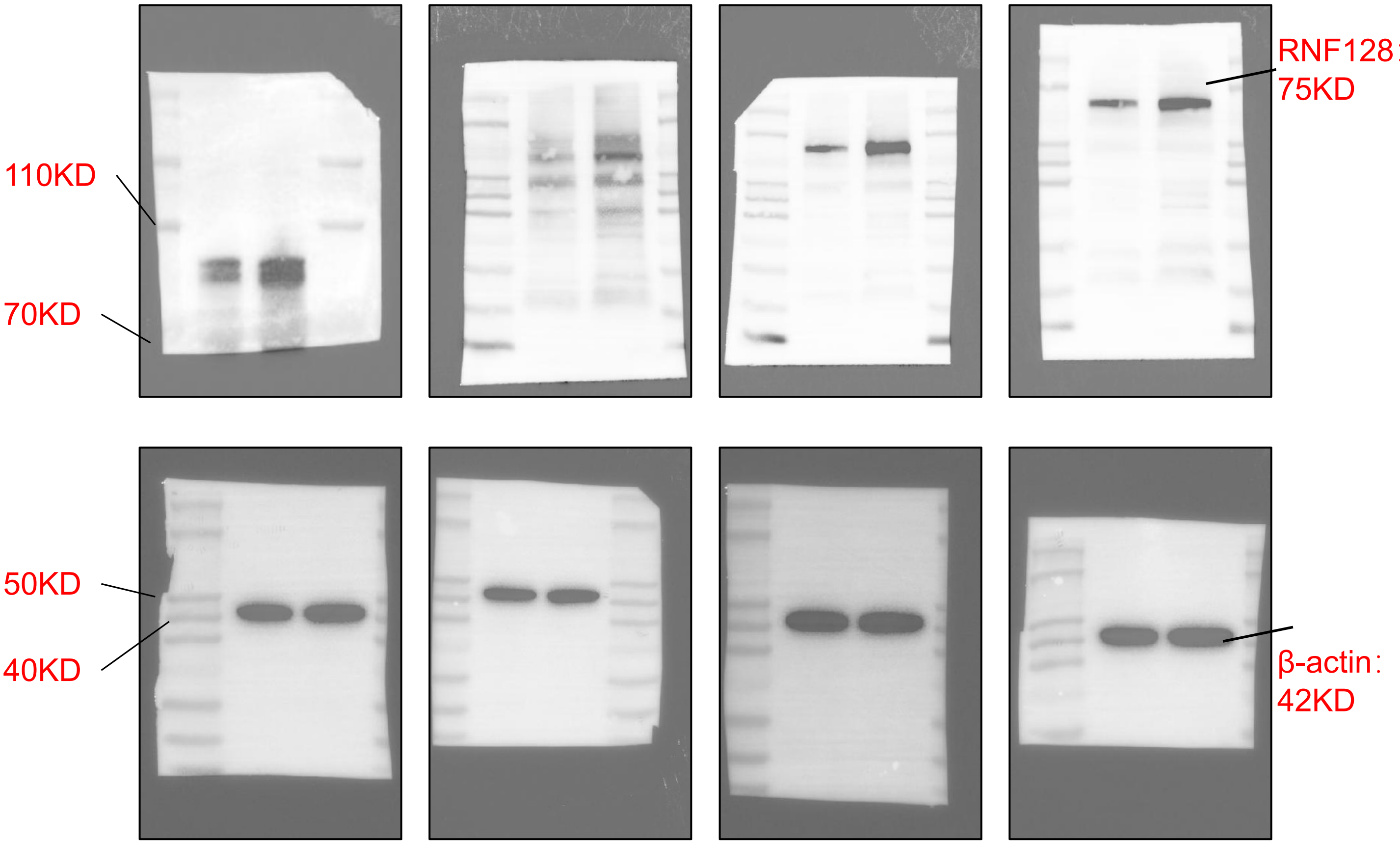

Patients 13-16

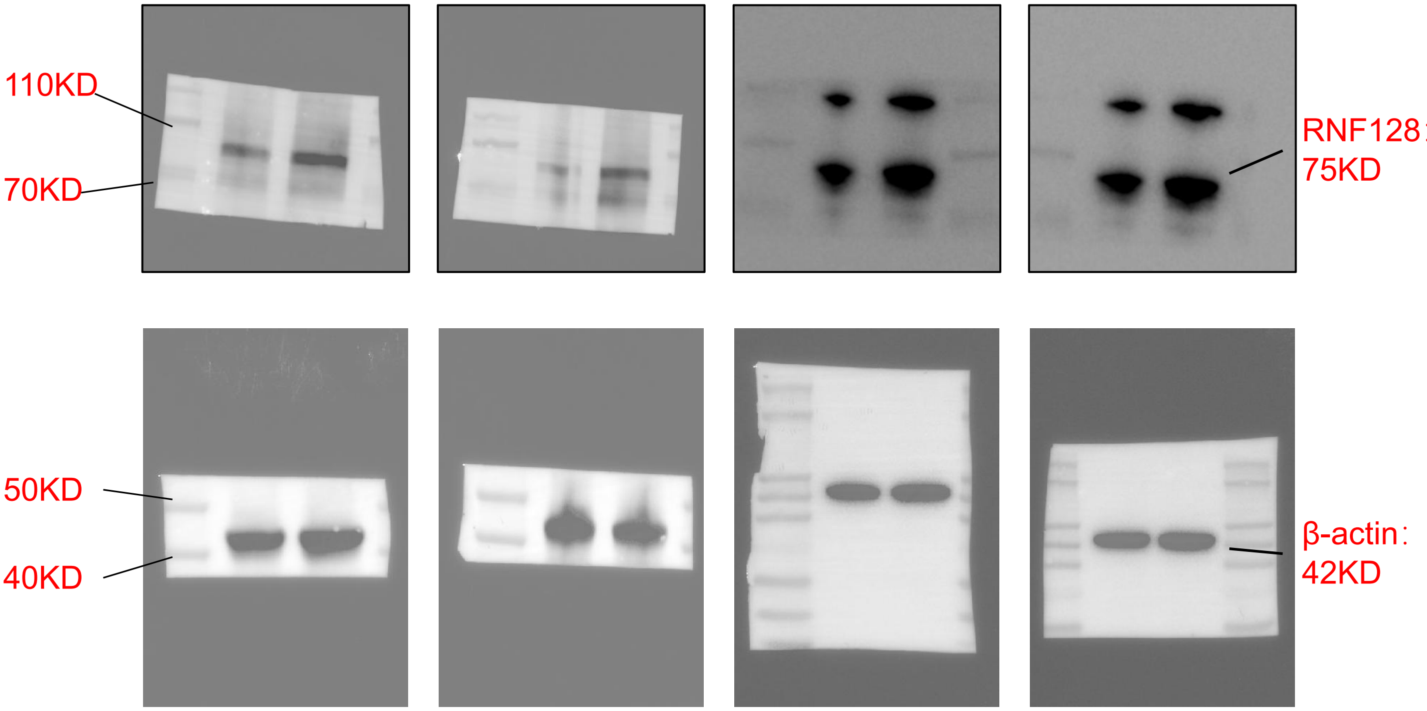

Patients 17-20

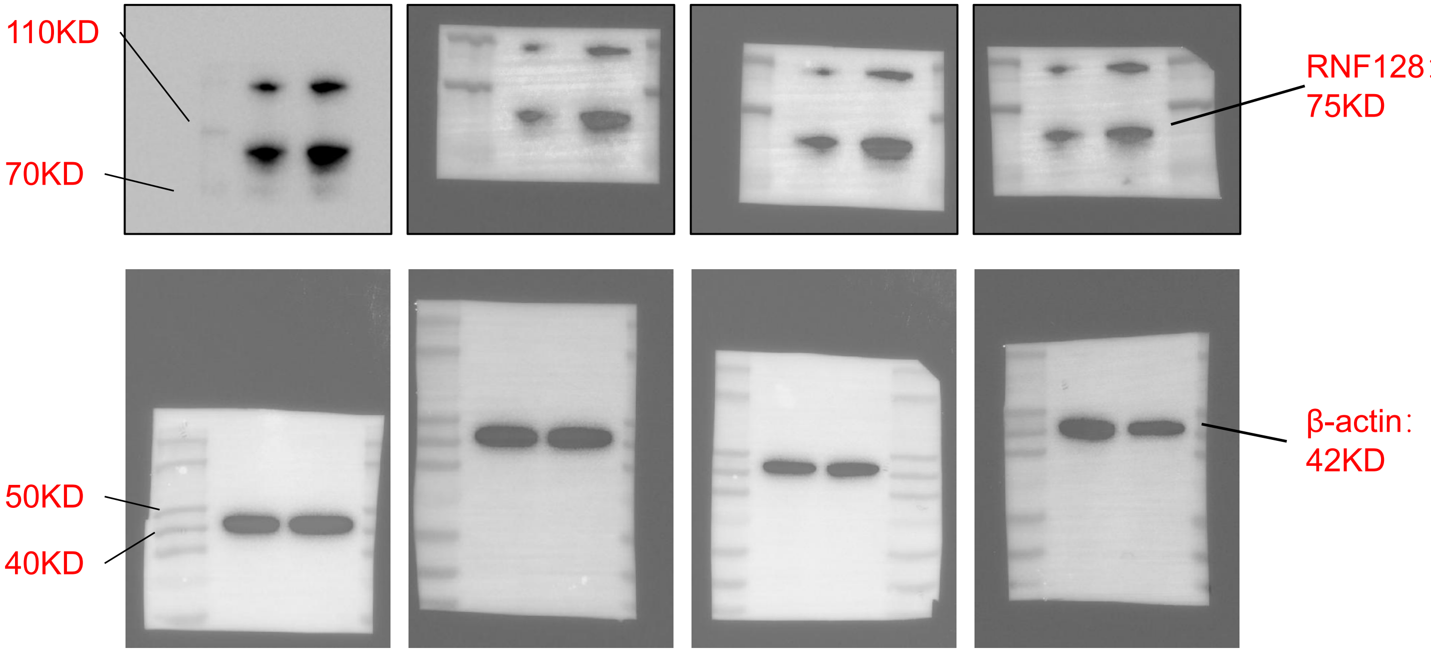

Patients 21-24

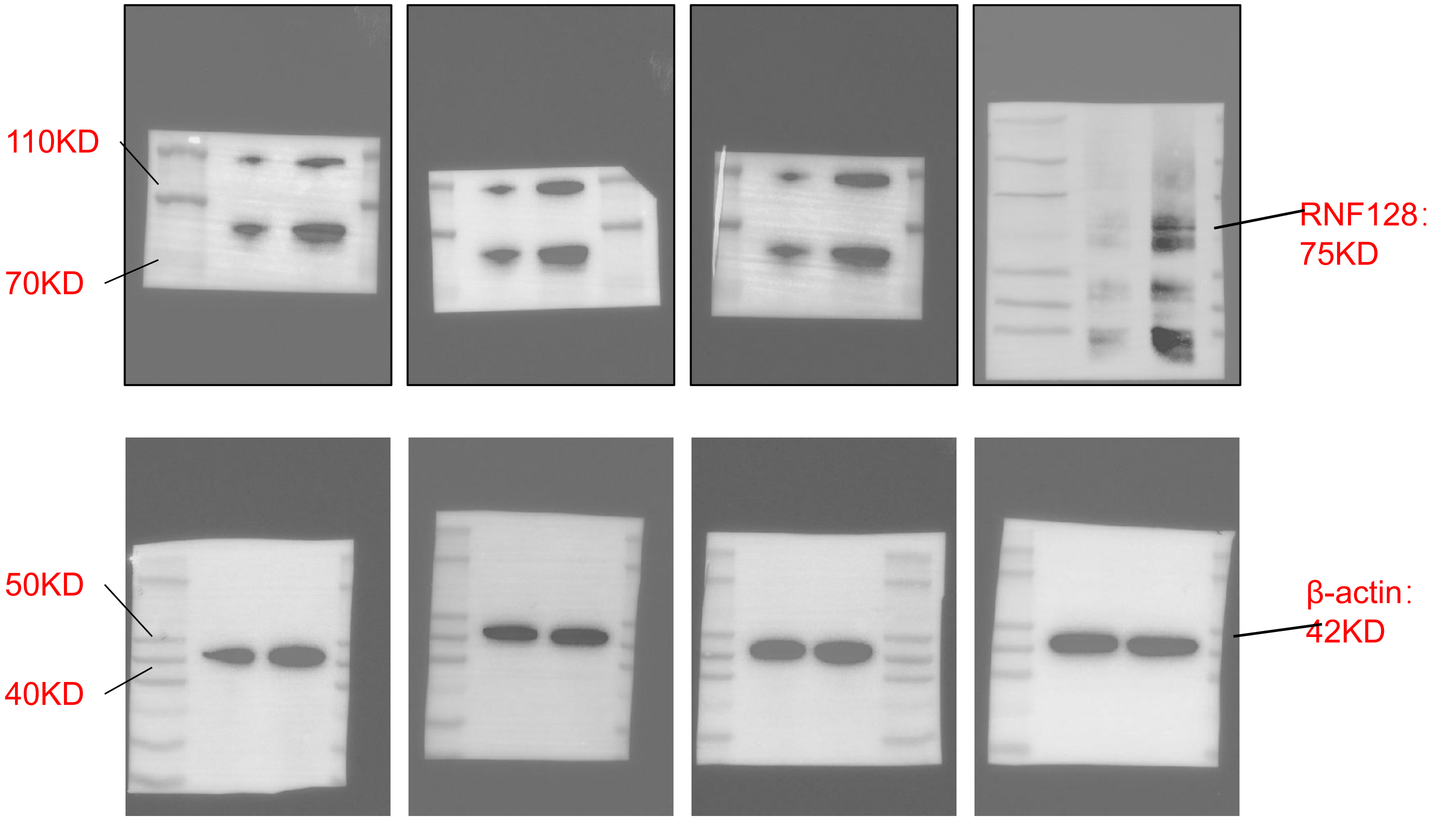

Patients 25-28

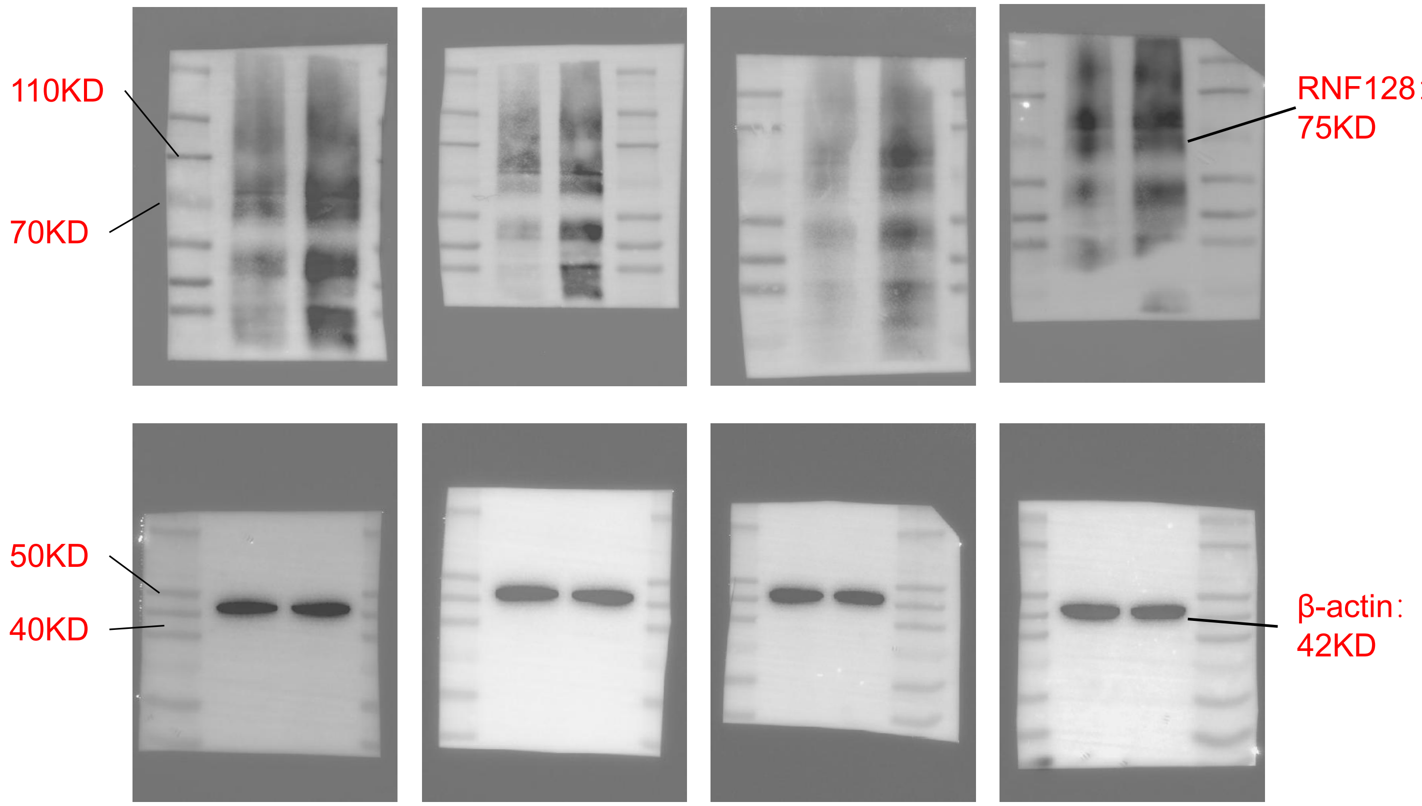

Patients 29-30

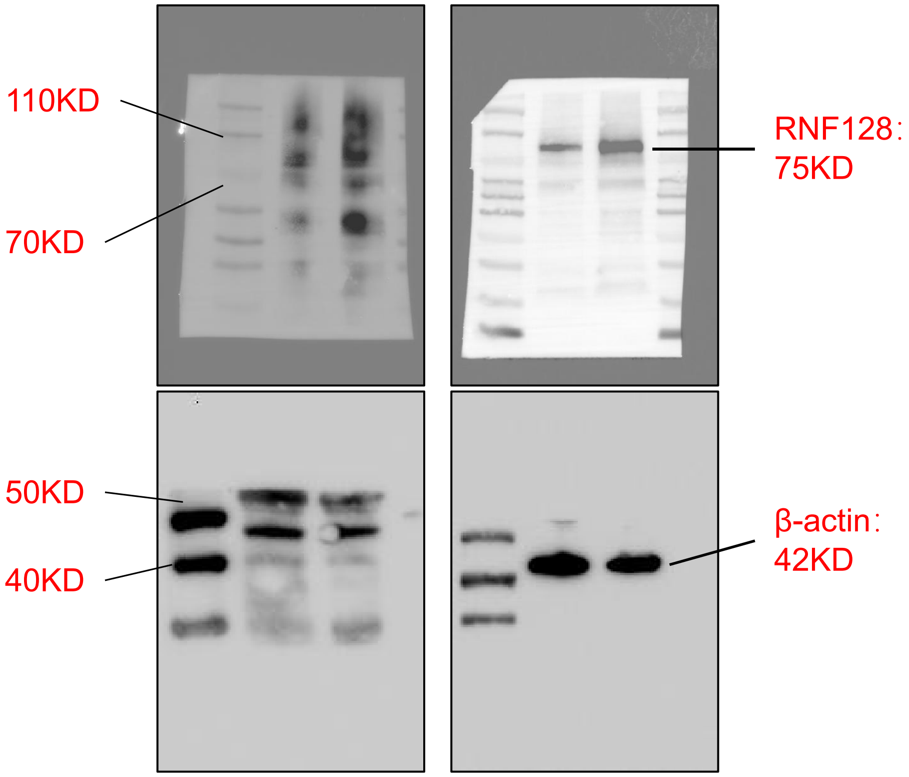

Patients 31-35

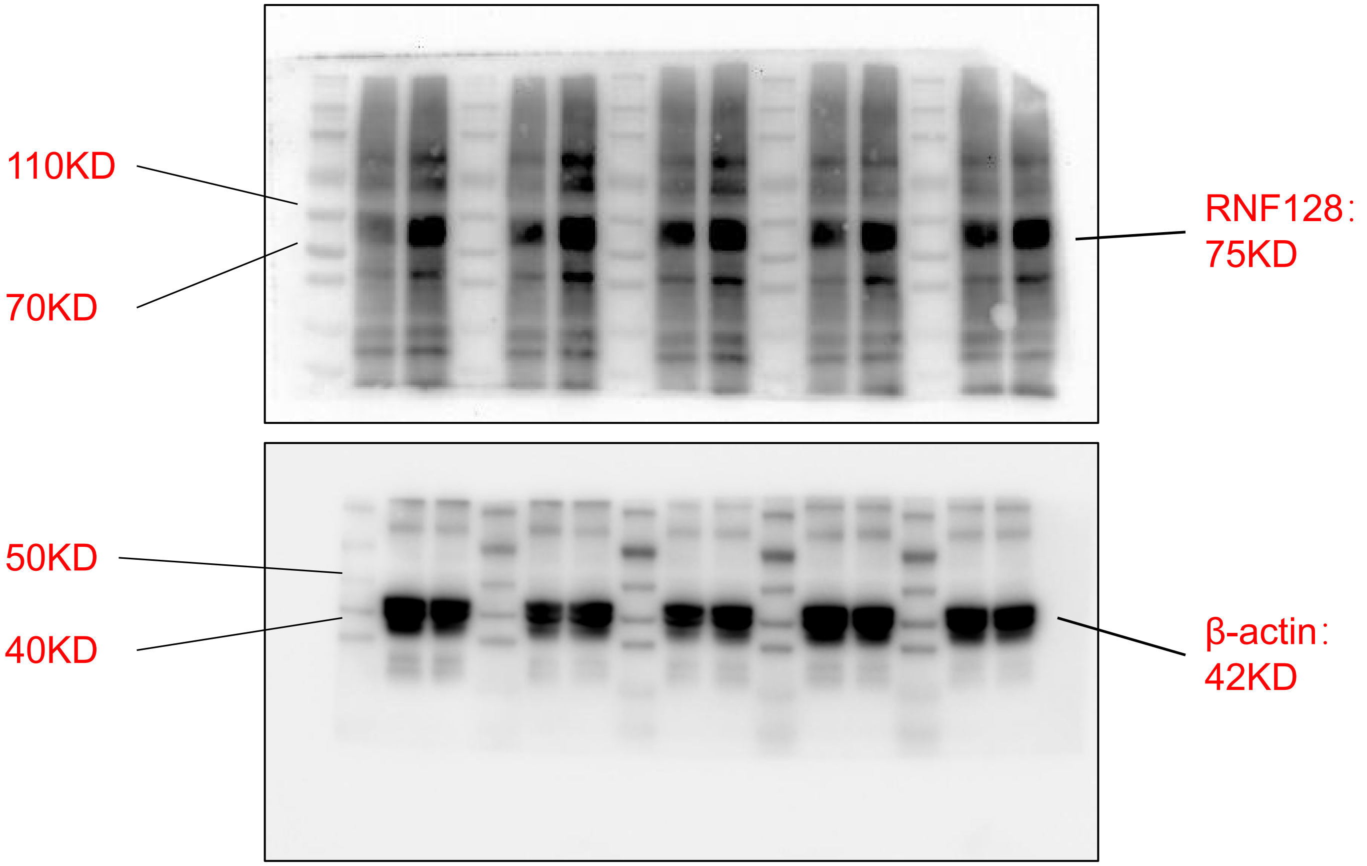

Patients 36-40

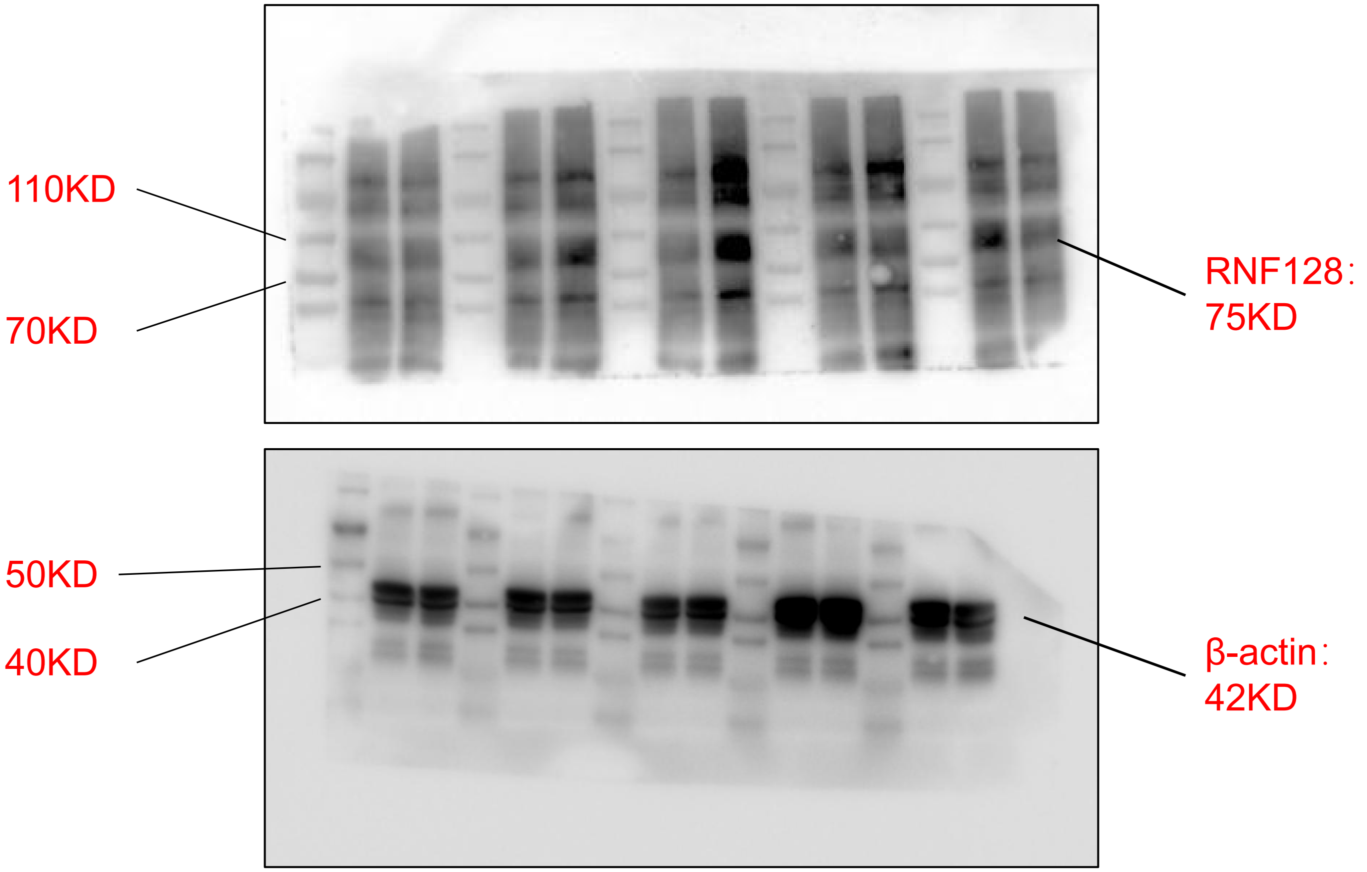

Patients 41-45

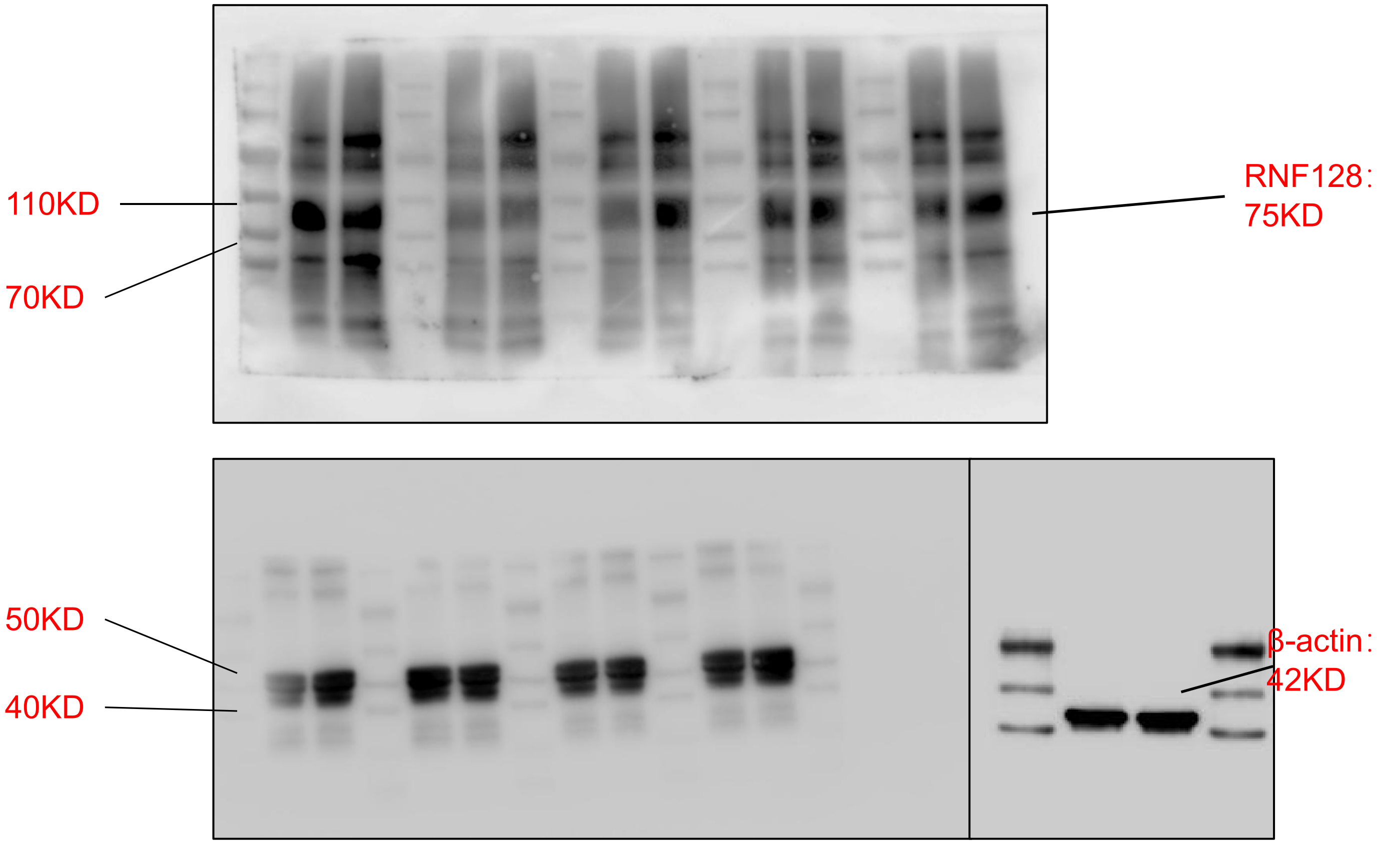

Patients 46-50

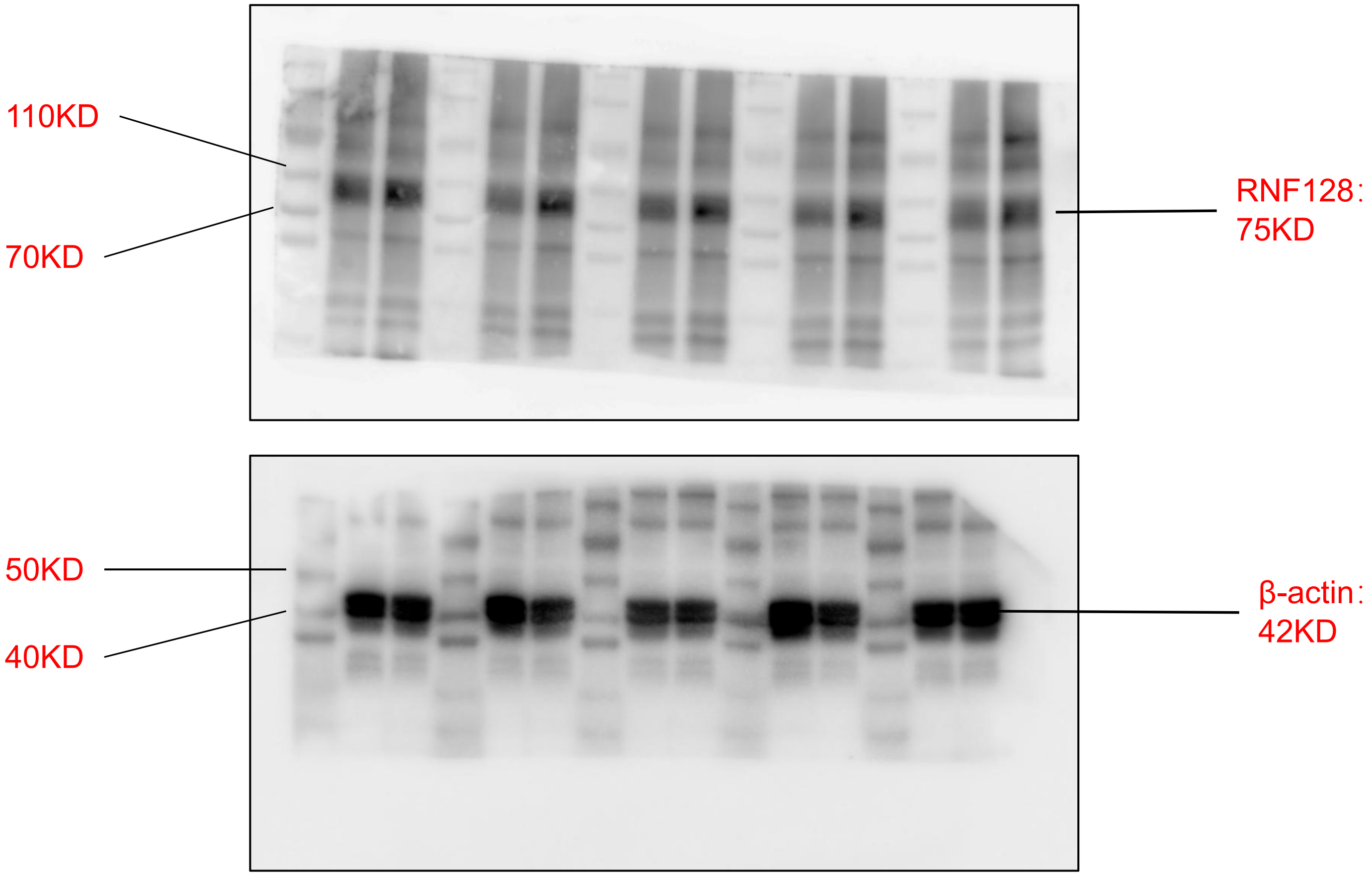

Figure1 f

On fig:

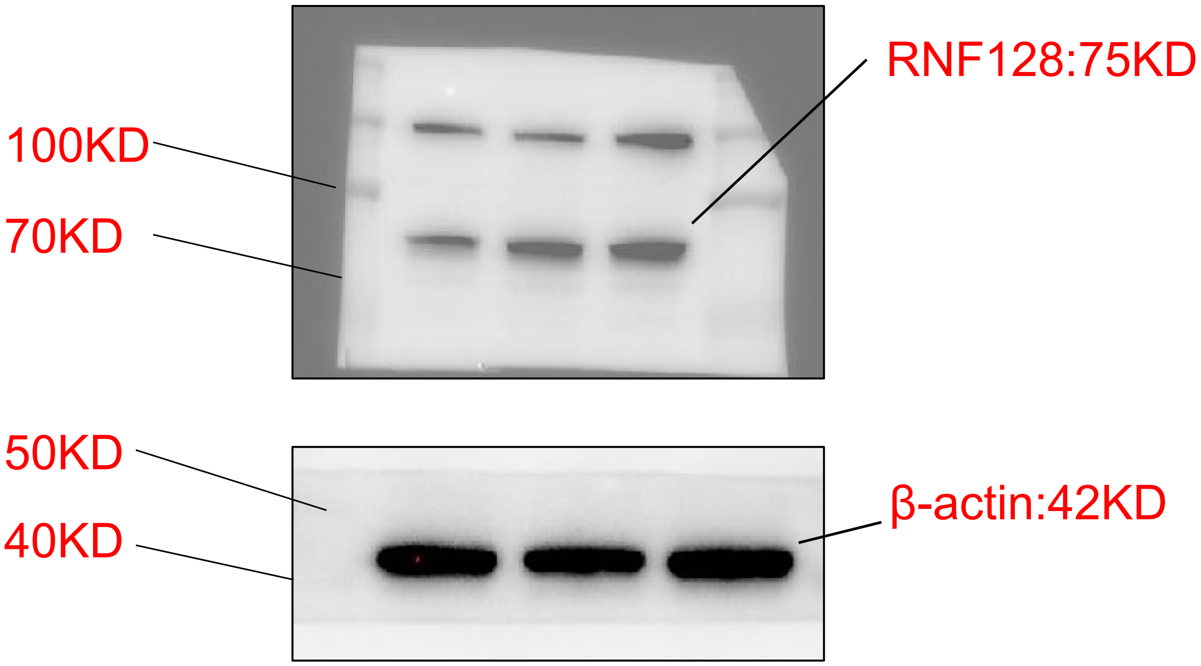

Repeat 1: **Repeat 2:**

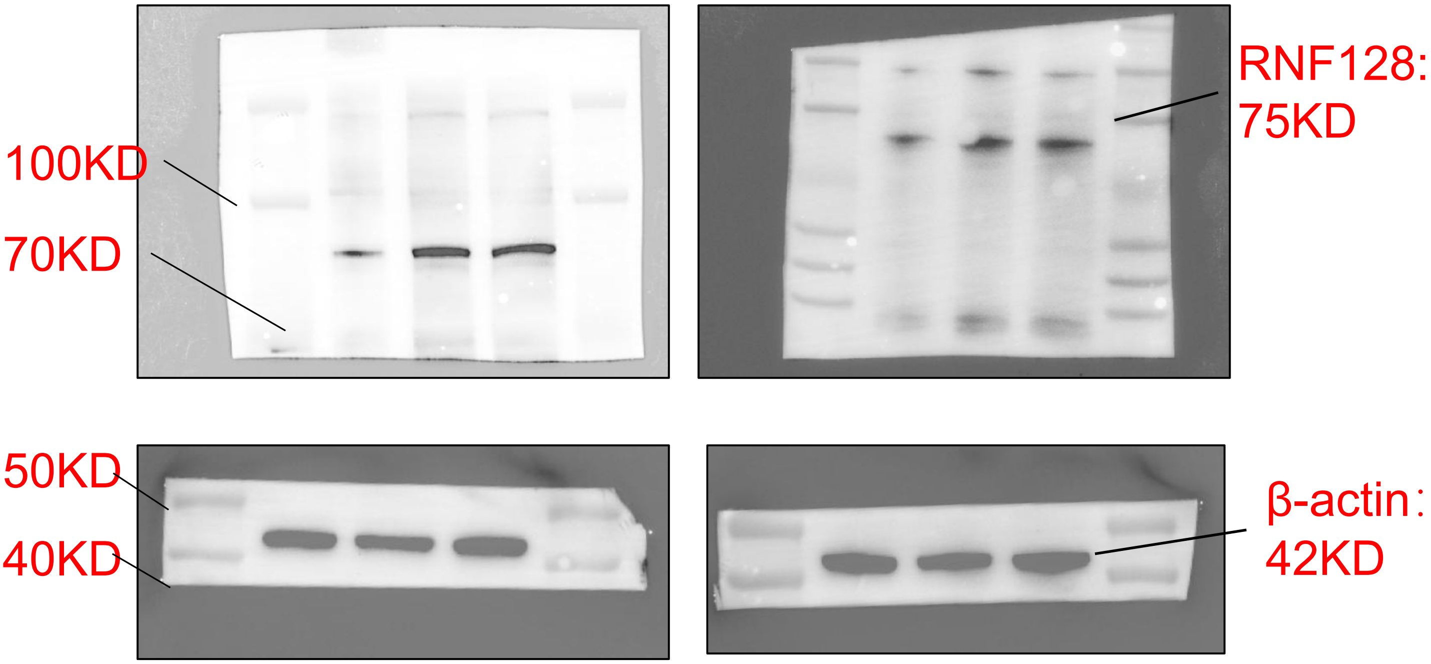

Figure3 b

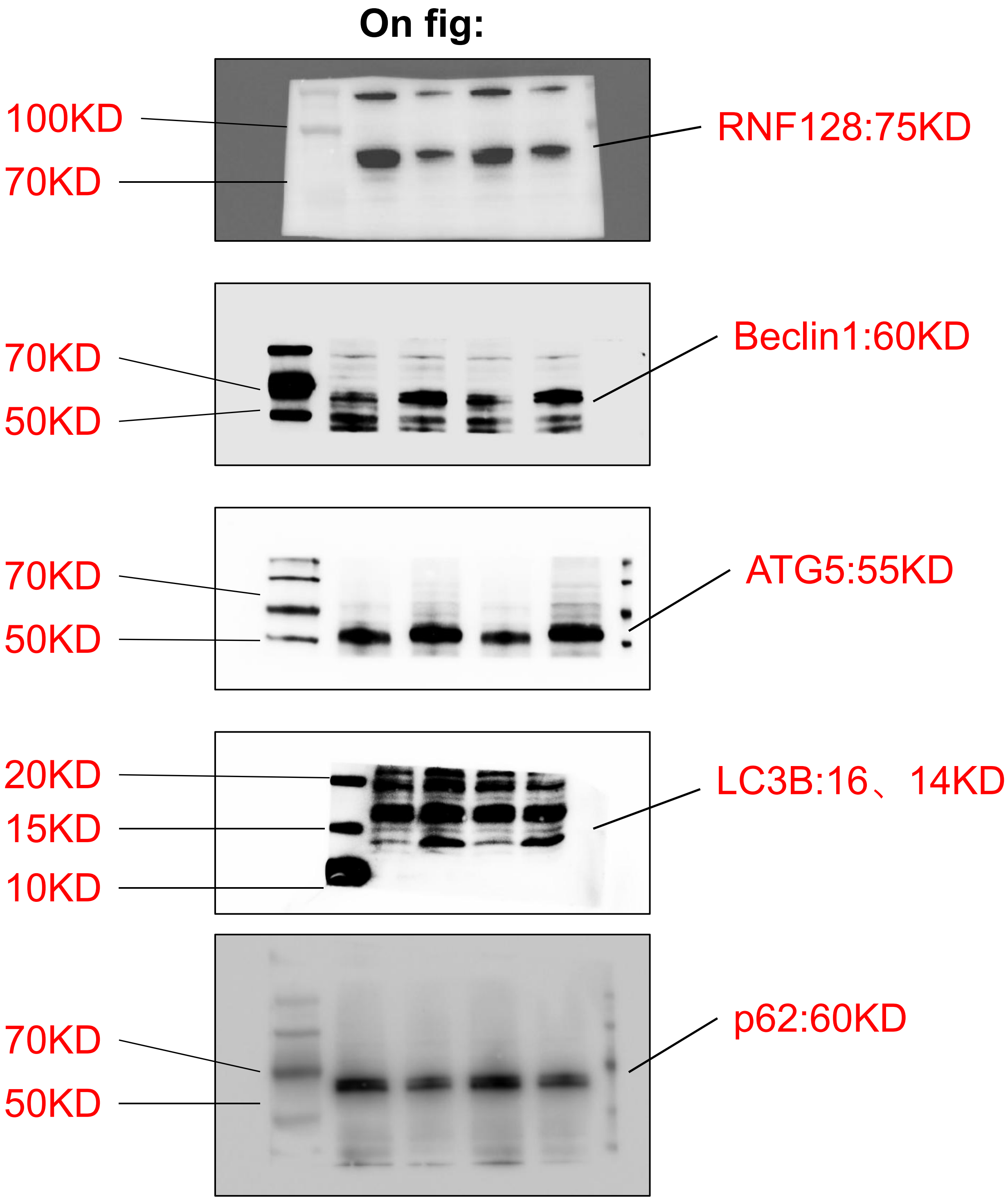
On fig:
100KD
70KD
RNF128:75KD
70KD
50KD
Beclin1:60KD
70KD
50KD
ATG5:55KD
20KD
15KD
10KD
LC3B:16、14KD
70KD
50KD
p62:60KD

Figure3 b

On fig:

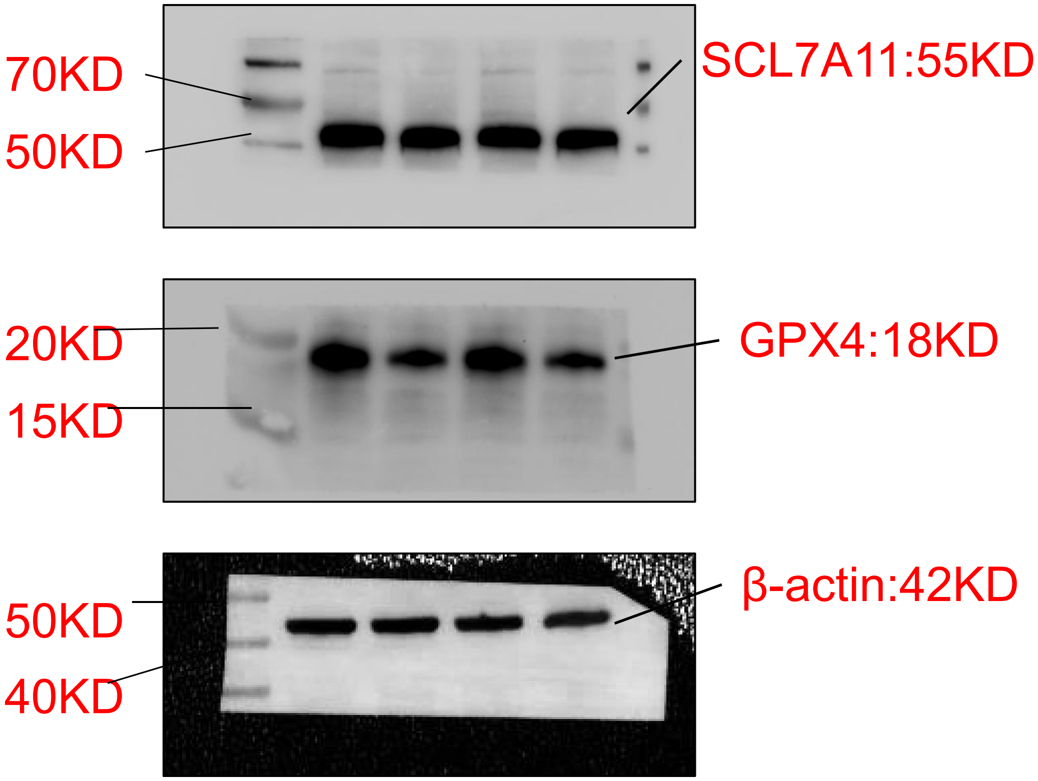

Figure3 b

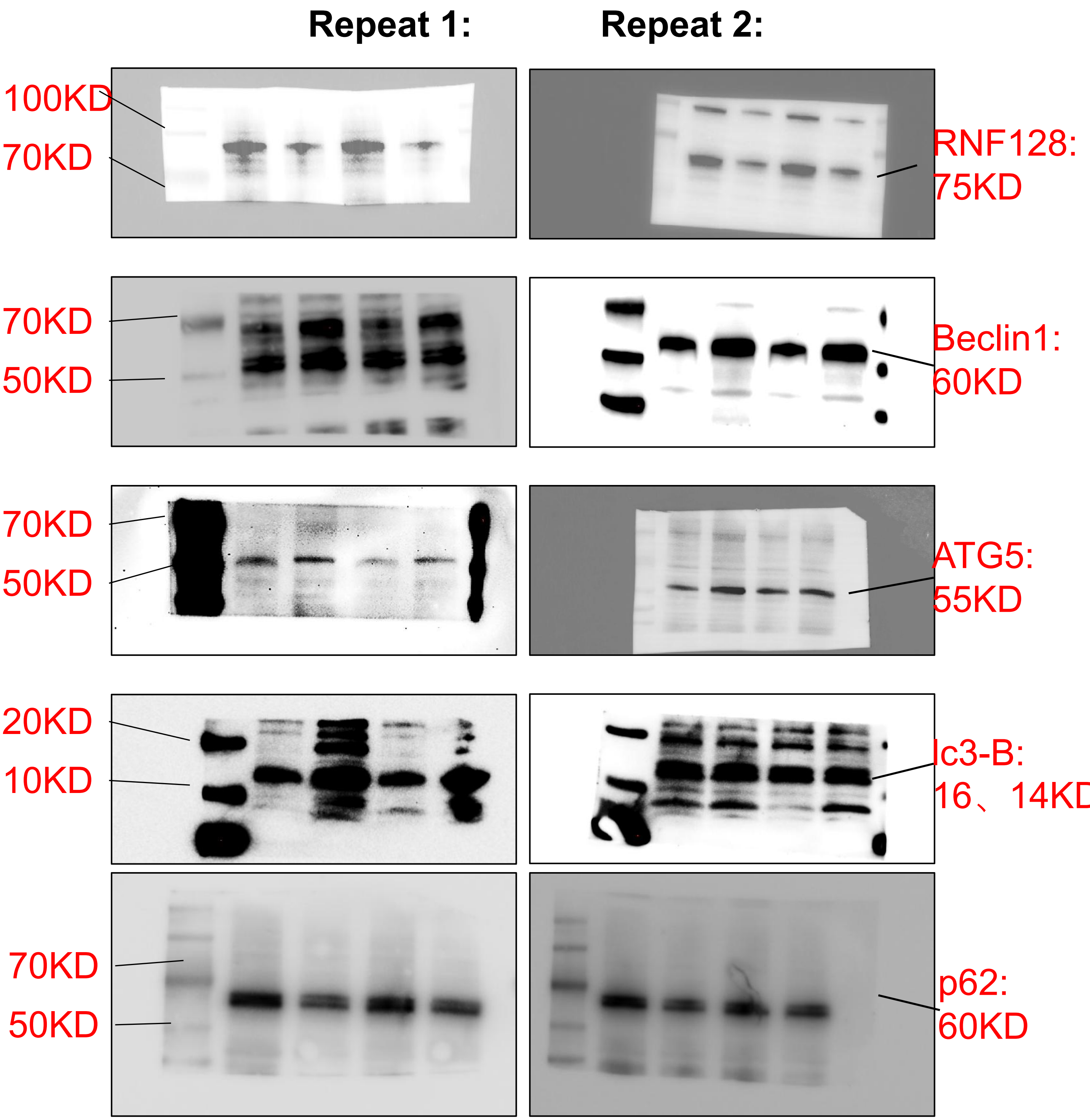
Repeat 1:
Repeat 2:
100KD
70KD
RNF128:
75KD
70KD
50KD
Beclin1:
60KD
70KD
50KD
ATG5:
55KD
20KD
10KD
lc3-B:
16、14KD
70KD
50KD
p62:
60KD

Figure3 b

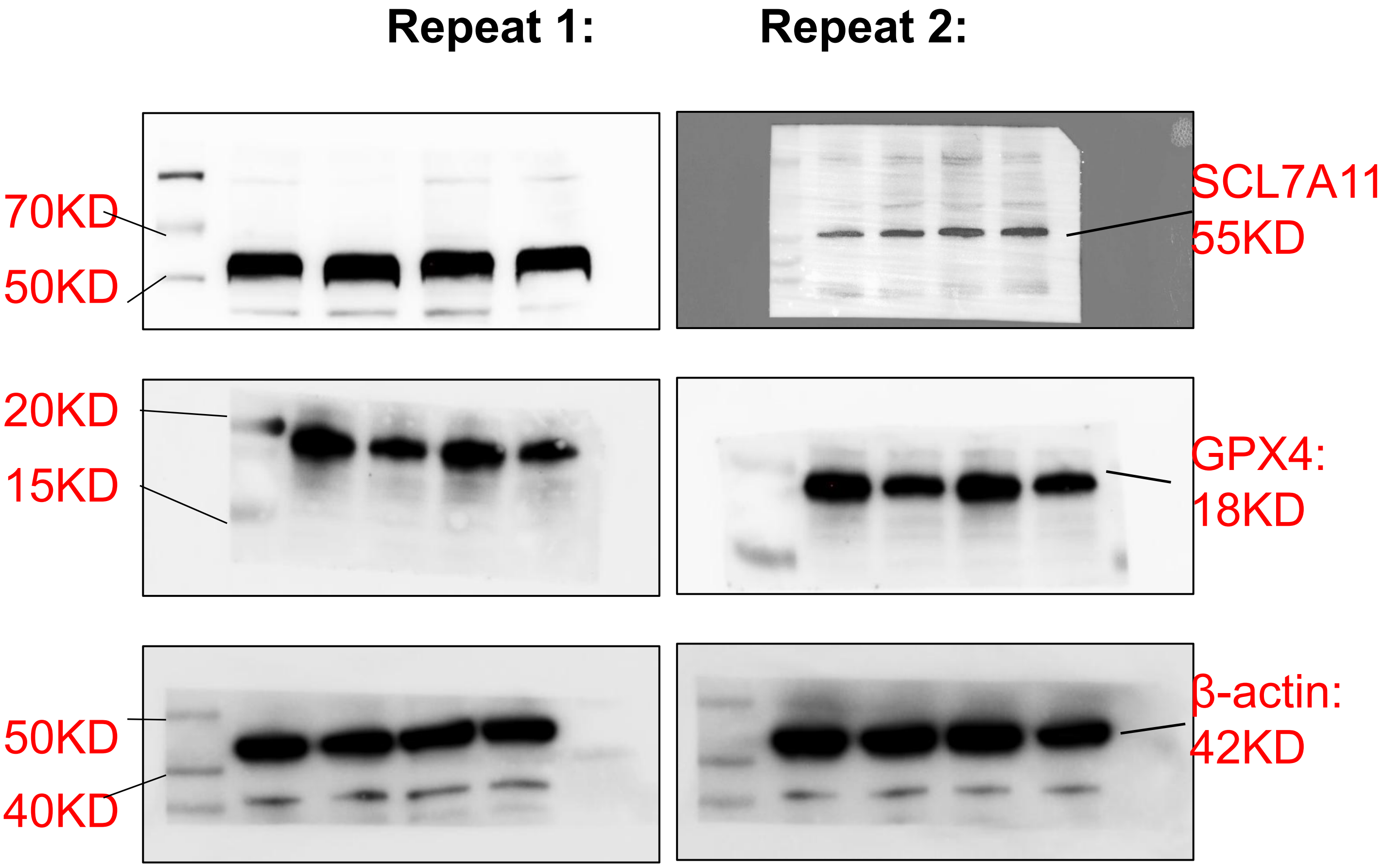
Repeat 1:
Repeat 2:
70KD
50KD
SCL7A11
55KD
20KD
15KD
GPX4:
18KD
50KD
40KD
β-actin:
42KD

Figure3 i

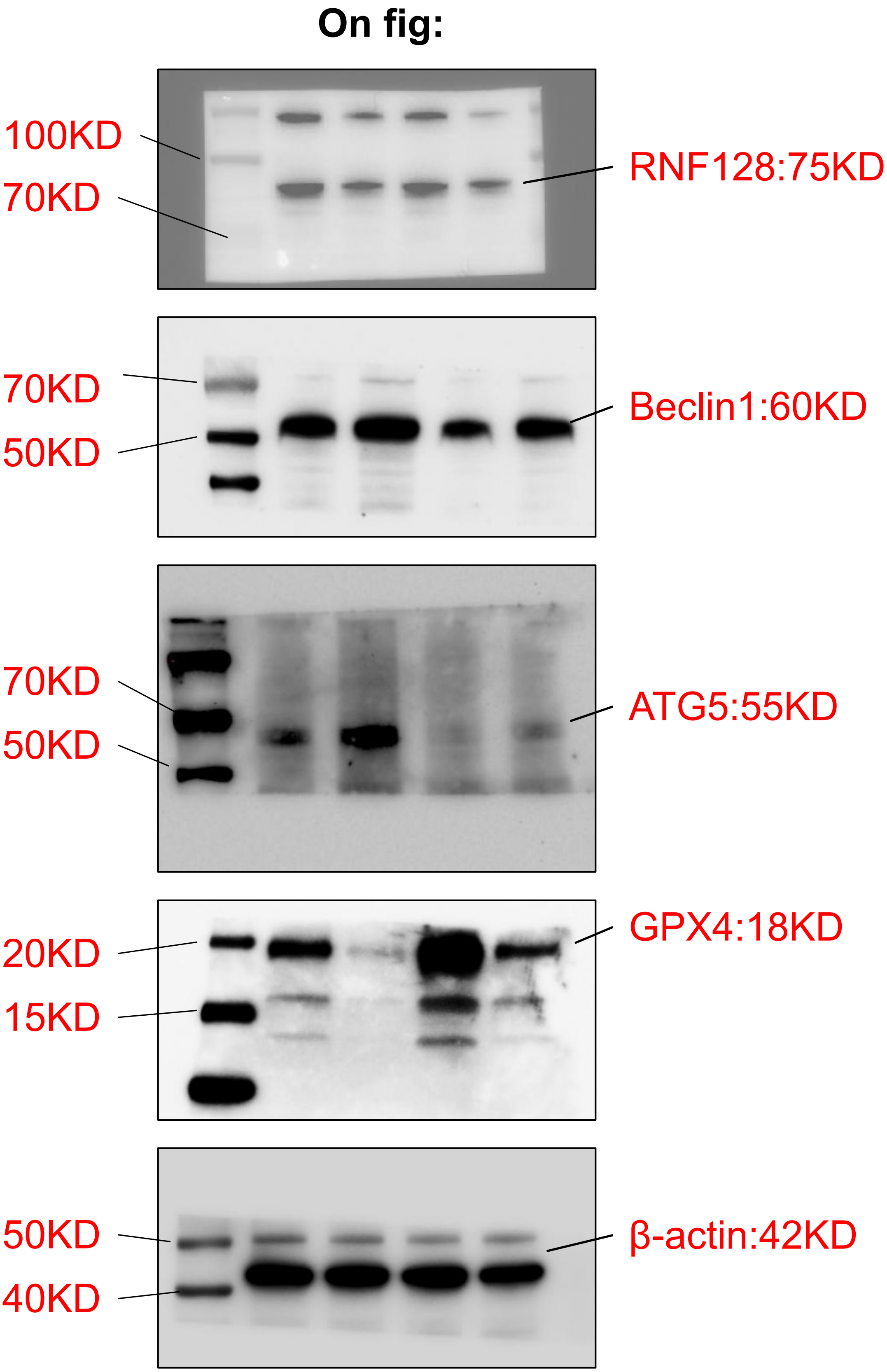
On fig:
100KD
70KD
RNF128:75KD
70KD
50KD
Beclin1:60KD
70KD
50KD
ATG5:55KD
20KD
15KD
GPX4:18KD
50KD
40KD
β-actin:42KD

Figure3 i

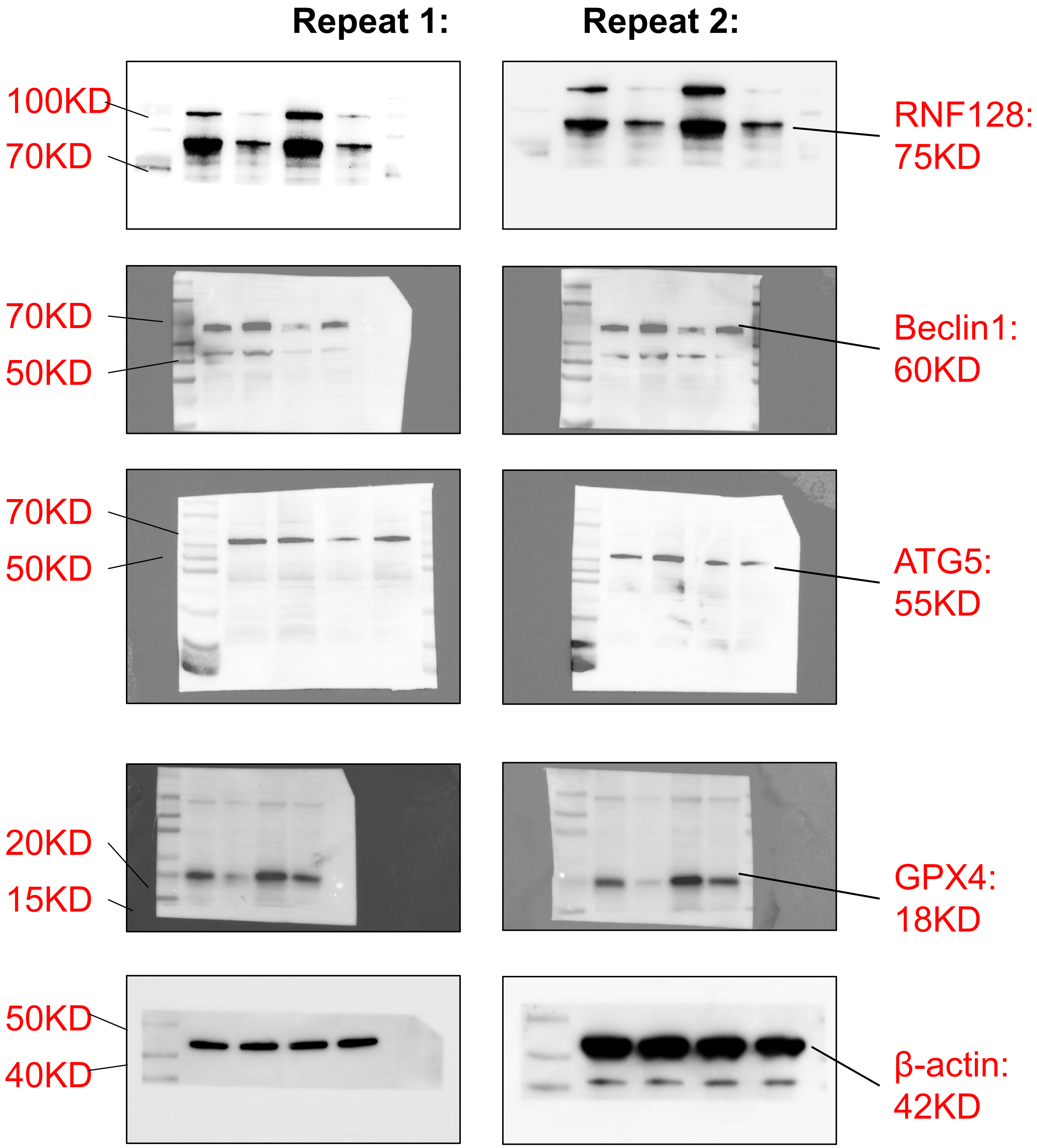
Repeat 1:
Repeat 2:
100KD
70KD
RNF128:
75KD
70KD
50KD
Beclin1:
60KD
70KD
50KD
ATG5:
55KD
20KD
15KD
GPX4:
18KD
50KD
40KD
β-actin:
42KD

Figure4 b

On fig:

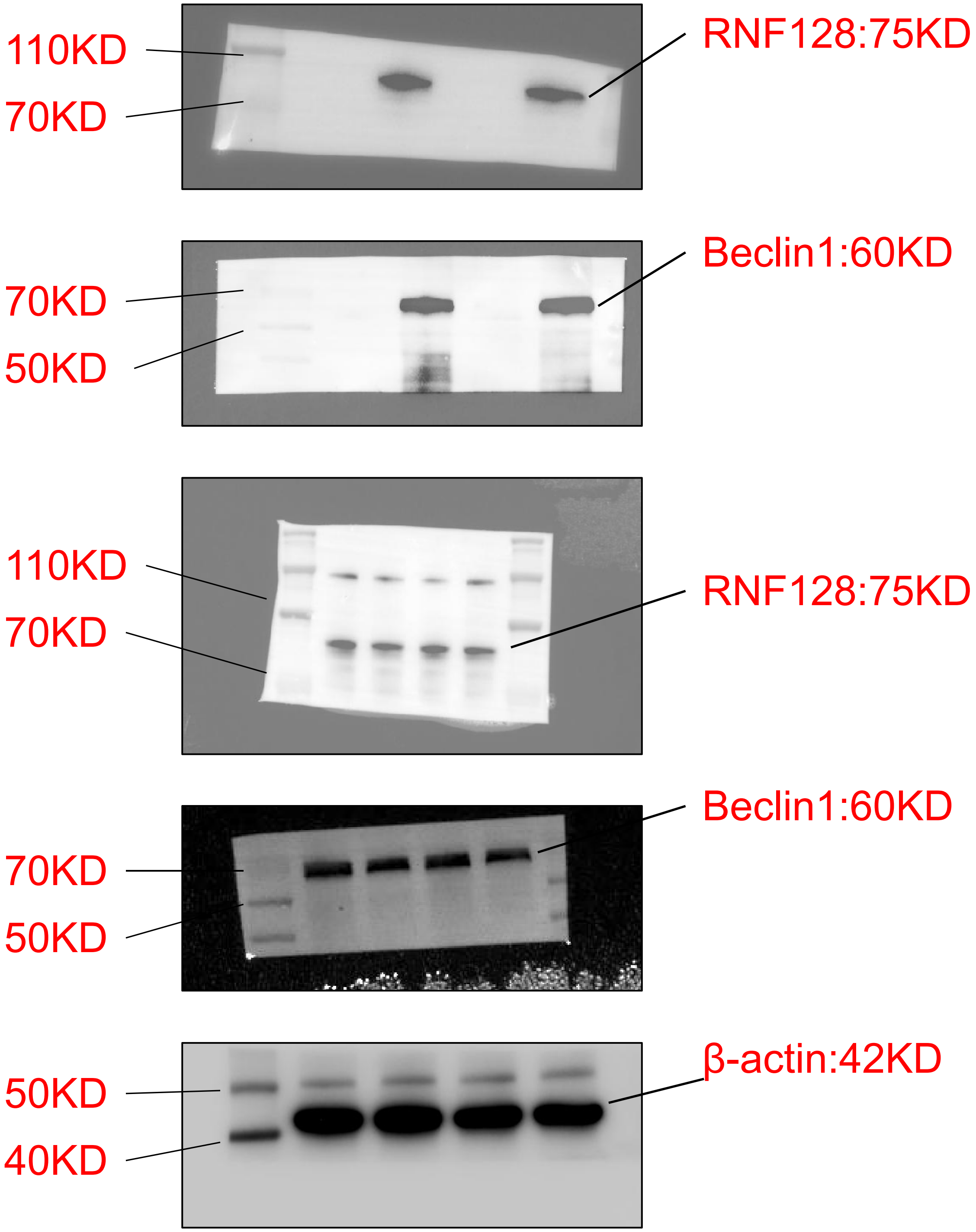

Figure4 b

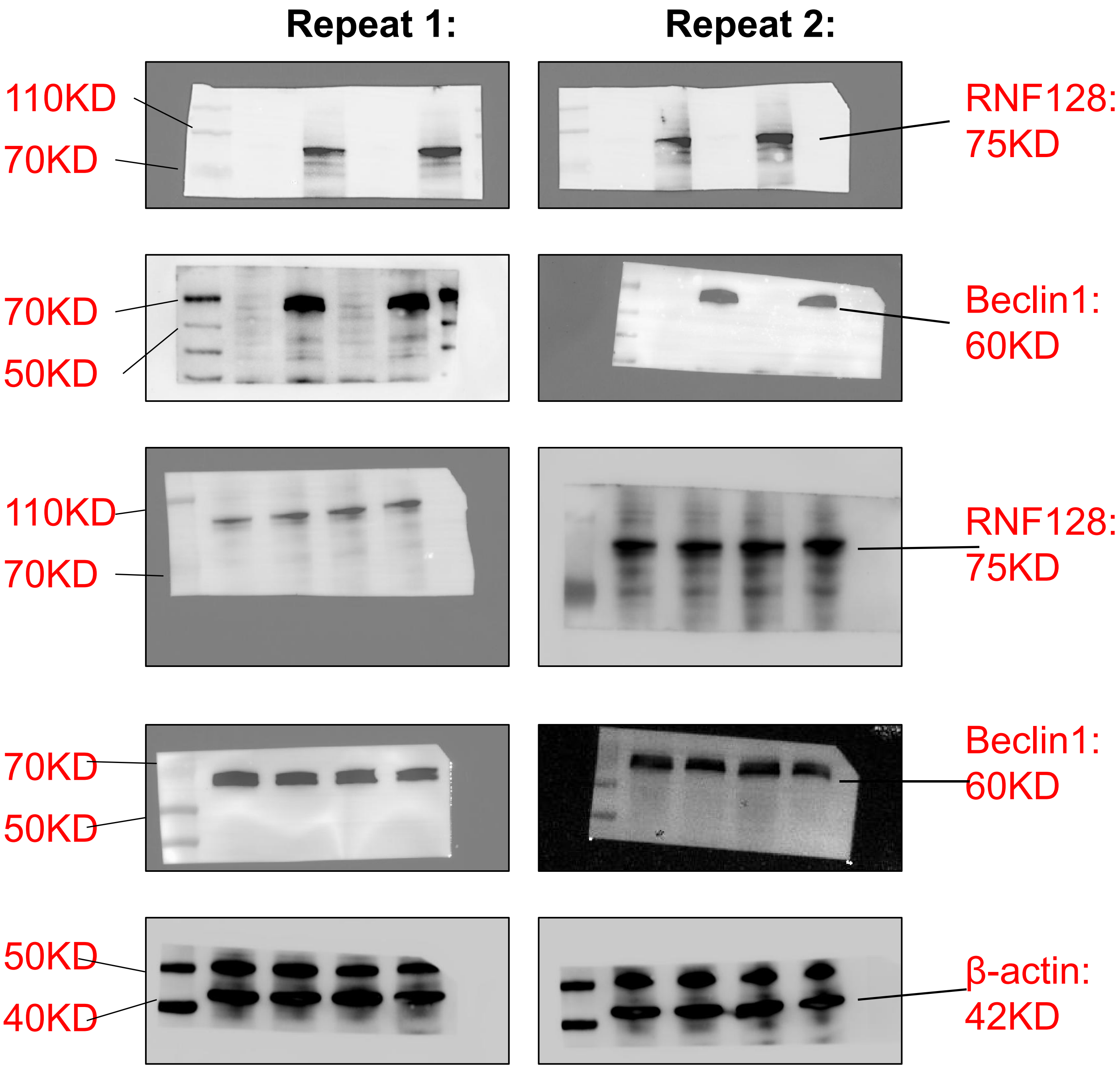

Repeat 1:
Repeat 2:
110KD
70KD
RNF128:
75KD
70KD
50KD
Beclin1:
60KD
110KD
70KD
RNF128:
75KD
70KD
50KD
Beclin1:
60KD
50KD
40KD
β-actin:
42KD

Figure4 c

On fig:

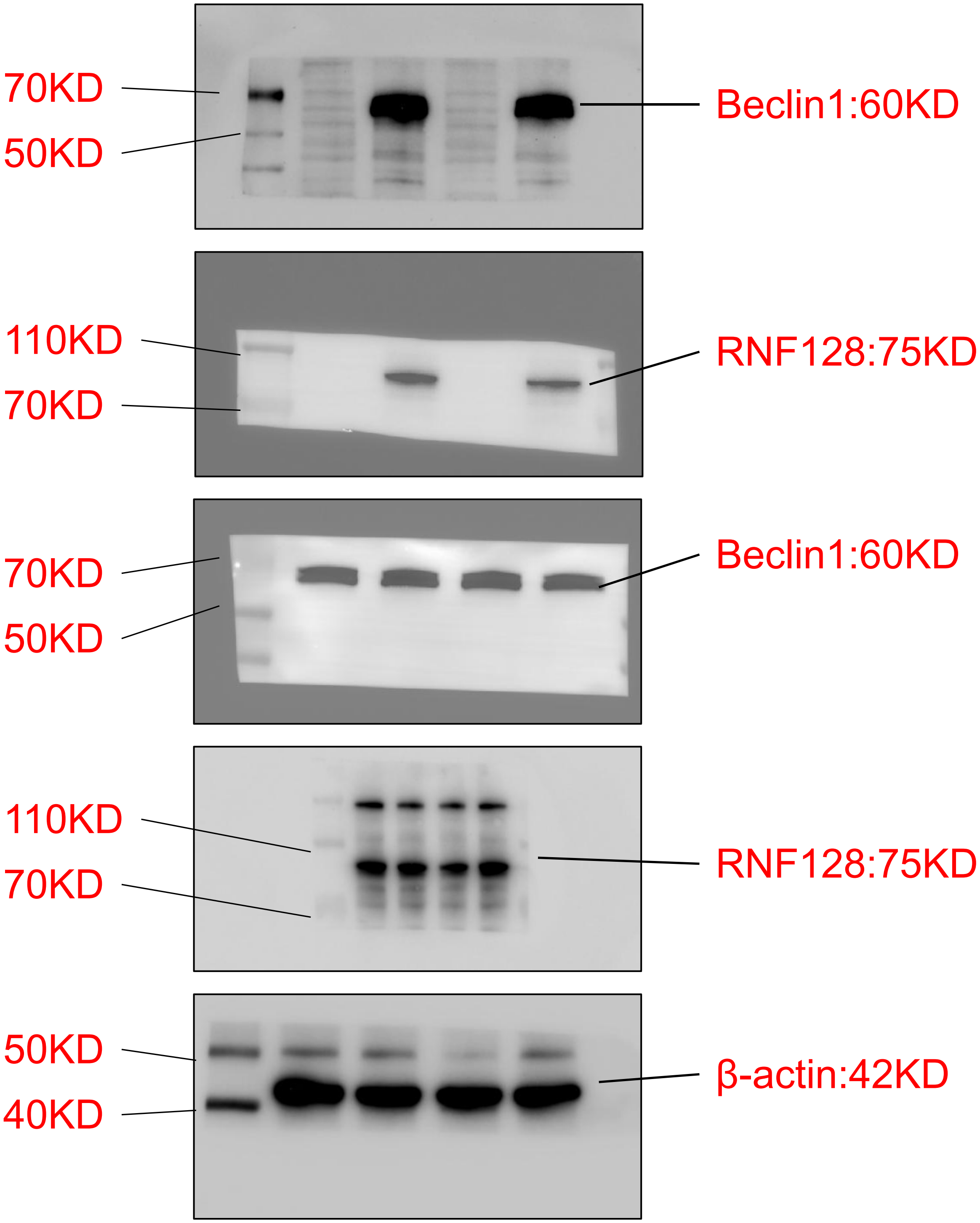

Figure4 c

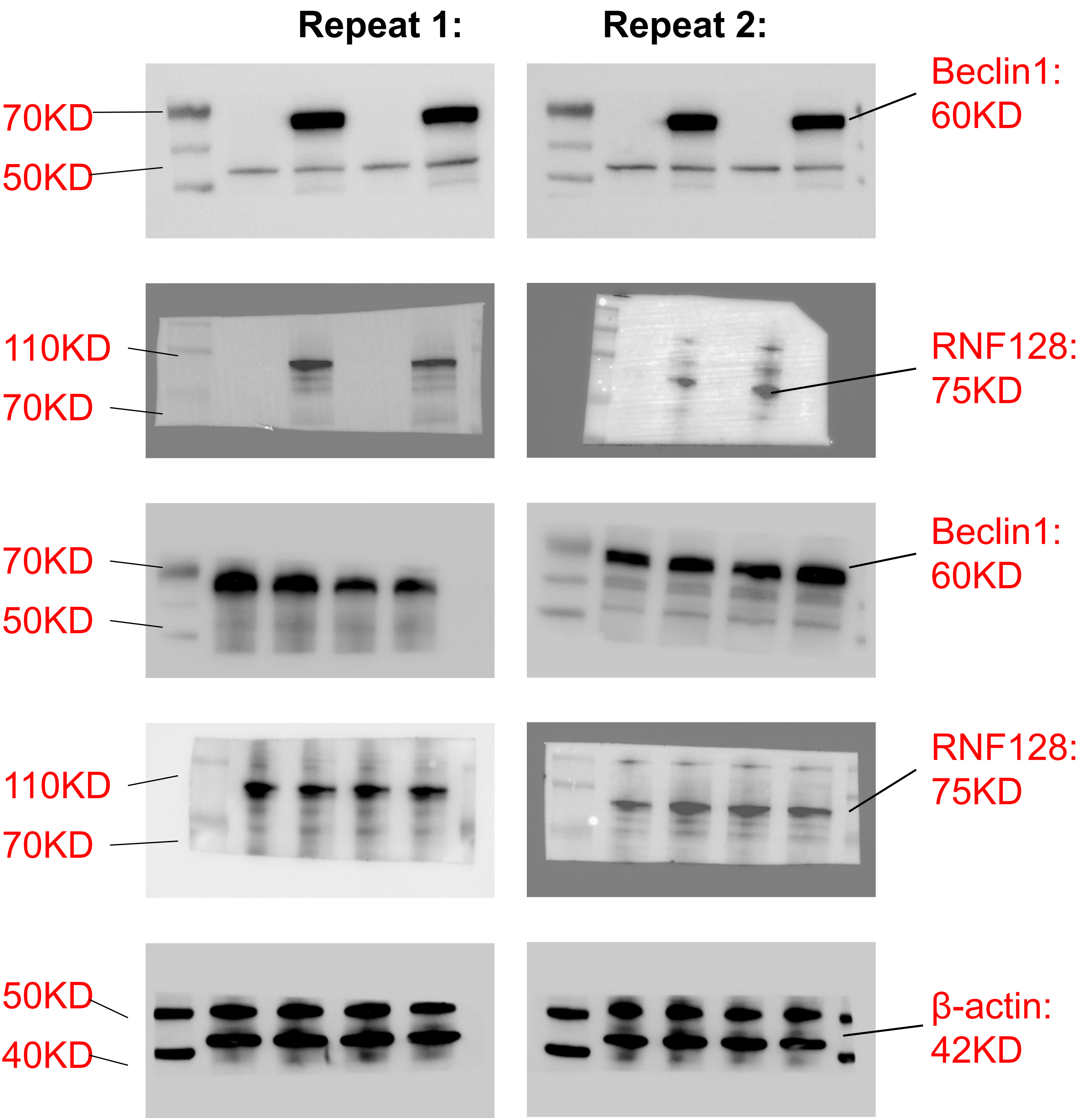
Repeat 1:
Repeat 2:
70KD
50KD
Beclin1:
60KD
110KD
70KD
RNF128:
75KD
70KD
50KD
Beclin1:
60KD
110KD
70KD
RNF128:
75KD
50KD
40KD
β-actin:
42KD

Figure4 d

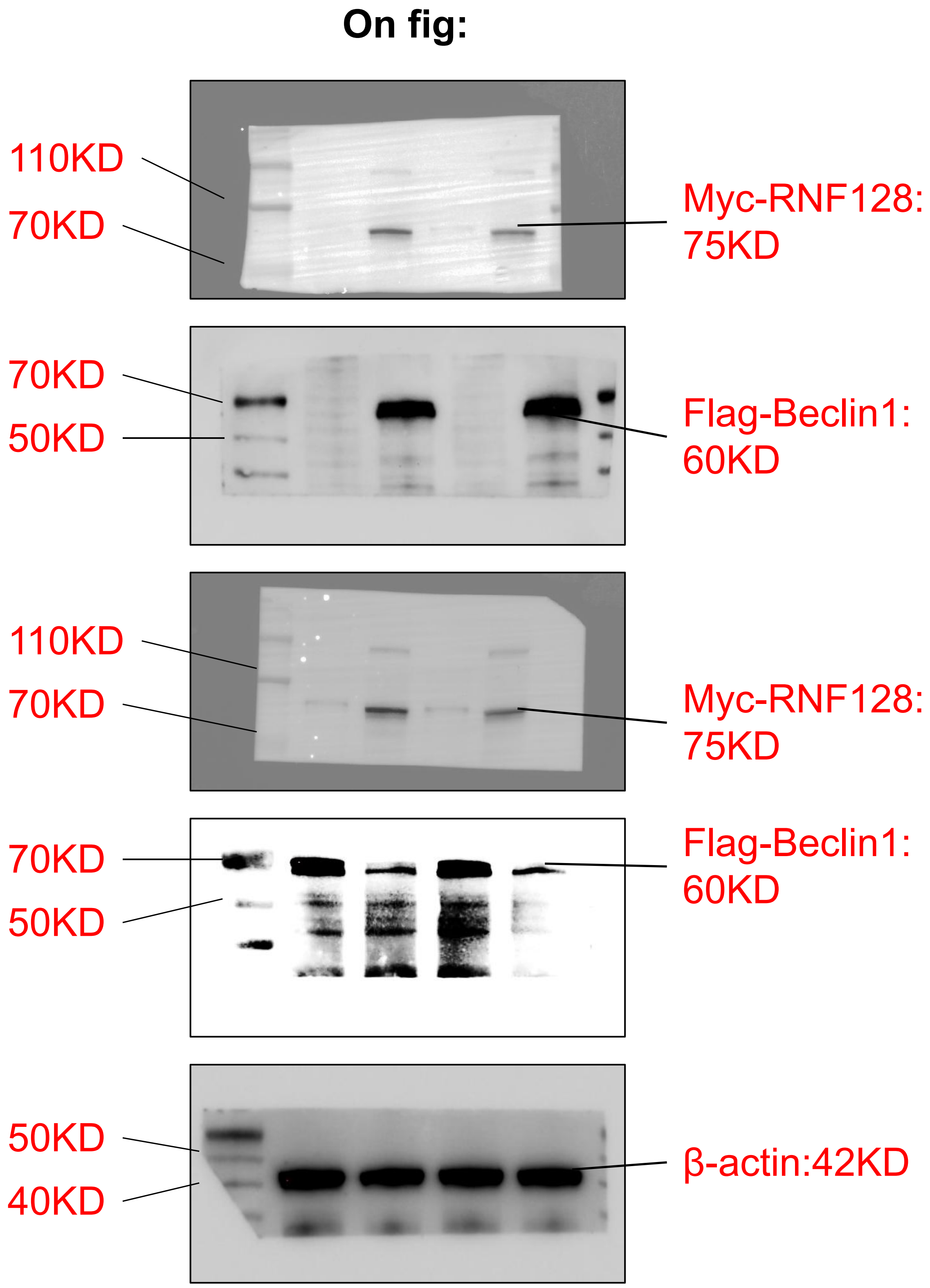
On fig:
110KD
70KD
Myc-RNF128:
75KD
70KD
50KD
Flag-Beclin1:
60KD
110KD
70KD
Myc-RNF128:
75KD
70KD
50KD
Flag-Beclin1:
60KD
50KD
40KD
β-actin:42KD

Figure4 d

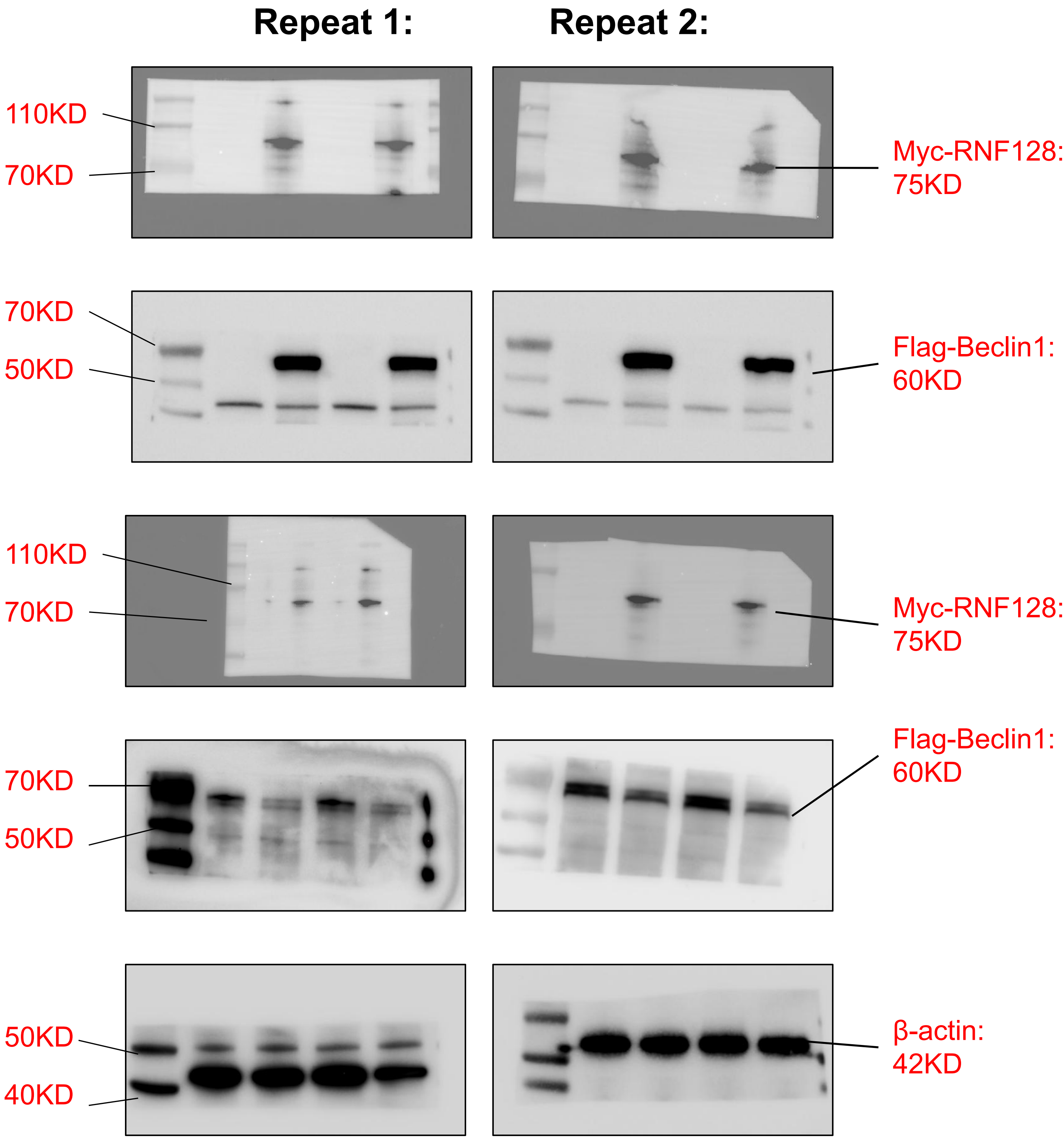

Repeat 1:
Repeat 2:
110KD
70KD
Myc-RNF128:
75KD
70KD
50KD
Flag-Beclin1:
60KD
110KD
70KD
Myc-RNF128:
75KD
70KD
50KD
Flag-Beclin1:
60KD
50KD
40KD
β-actin:
42KD

Figure4 e

On fig:

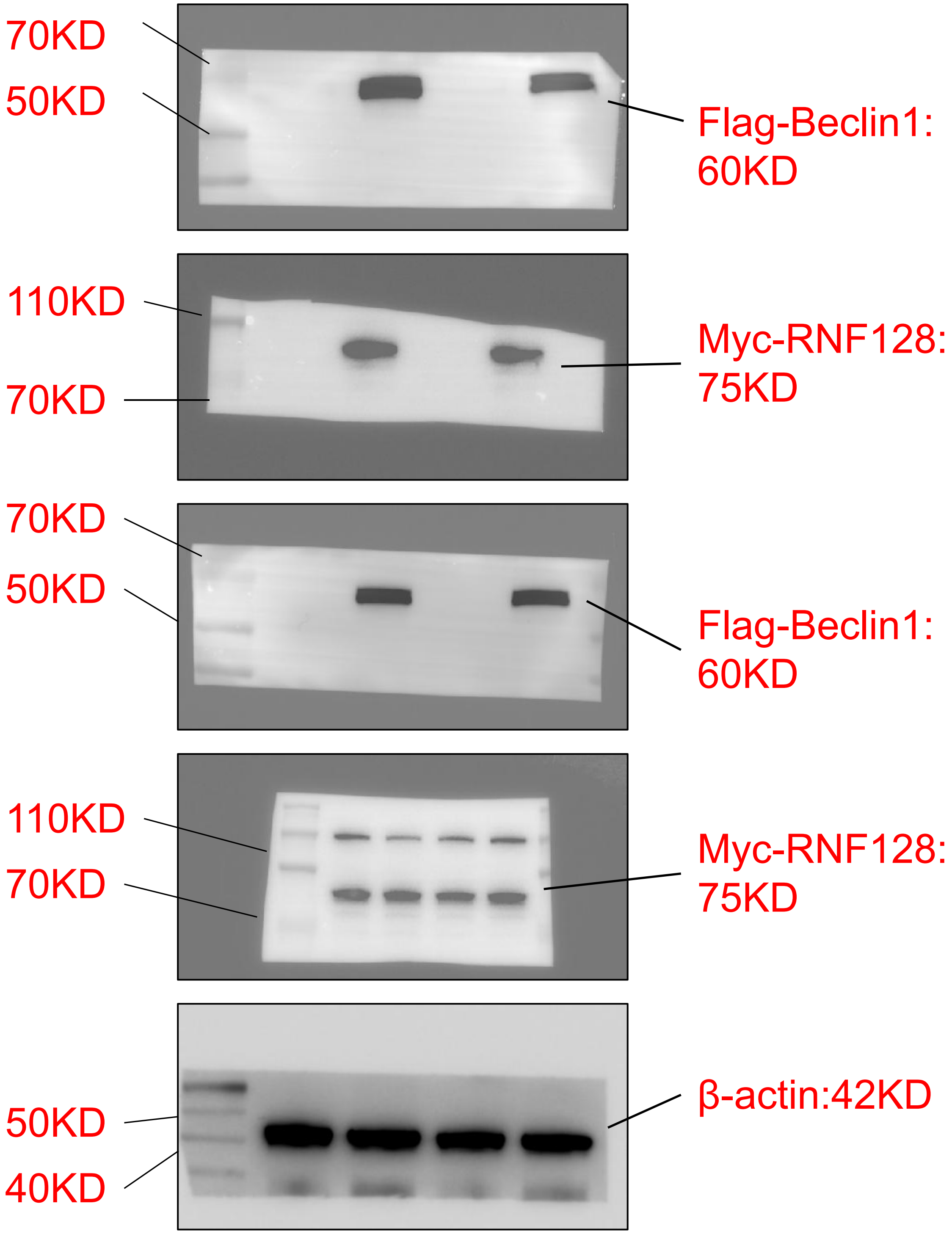

Figure4 e

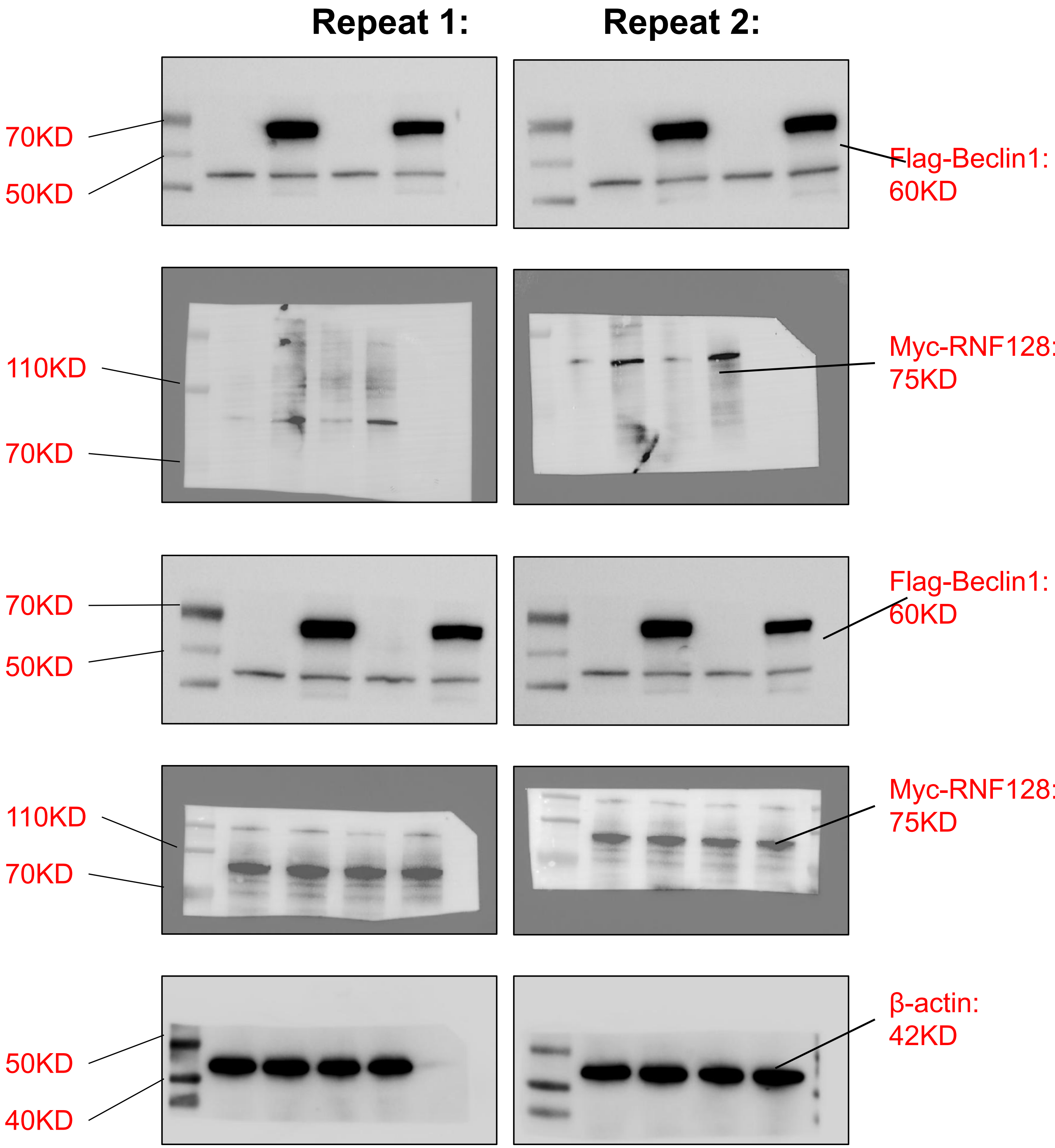
Repeat 1:
Repeat 2:
70KD
50KD
Flag-Beclin1:
60KD
110KD
70KD
Myc-RNF128:
75KD
70KD
50KD
Flag-Beclin1:
60KD
110KD
70KD
Myc-RNF128:
75KD
50KD
40KD
β-actin:
42KD

Figure4 h

On fig:

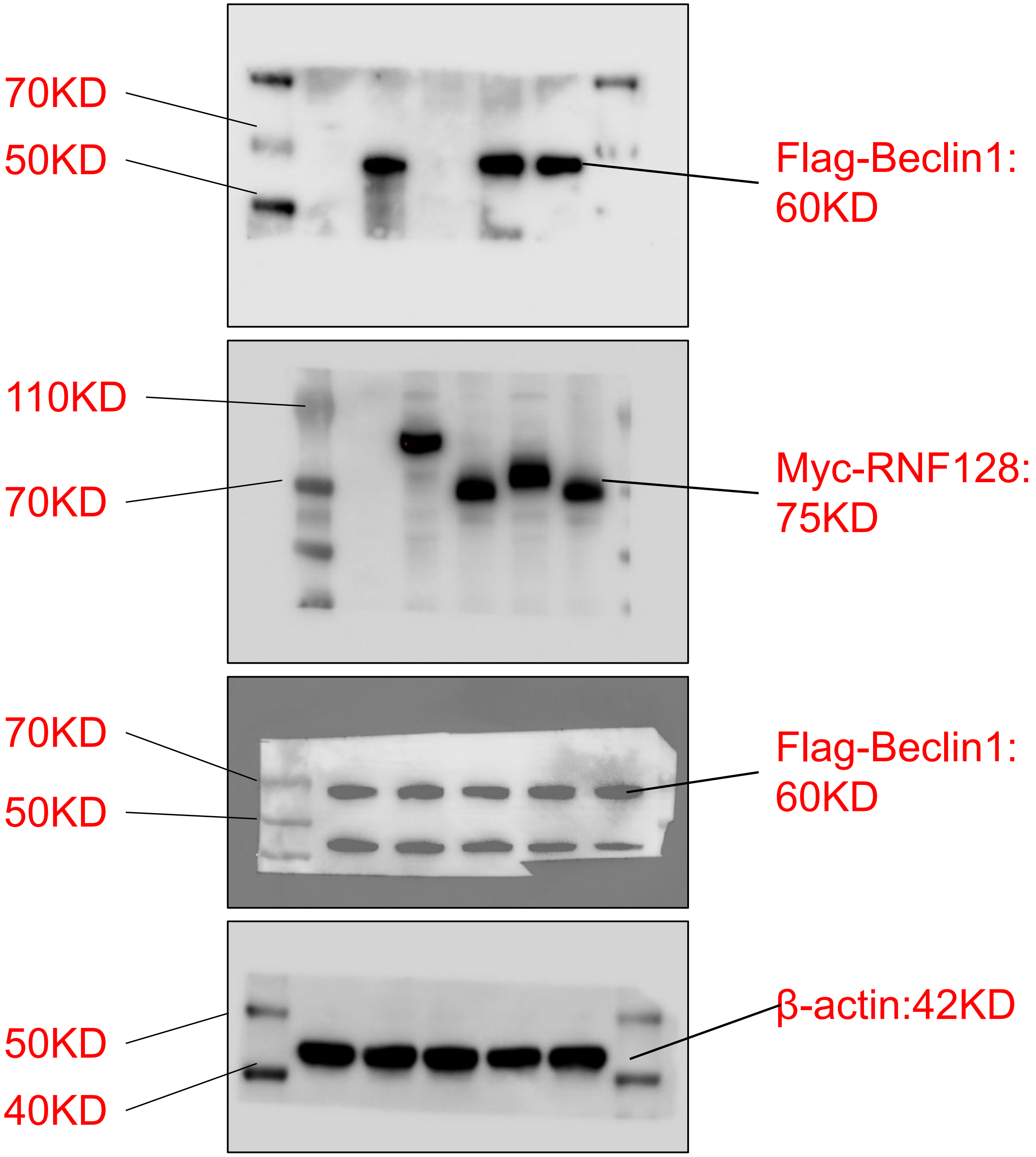

Figure4 h

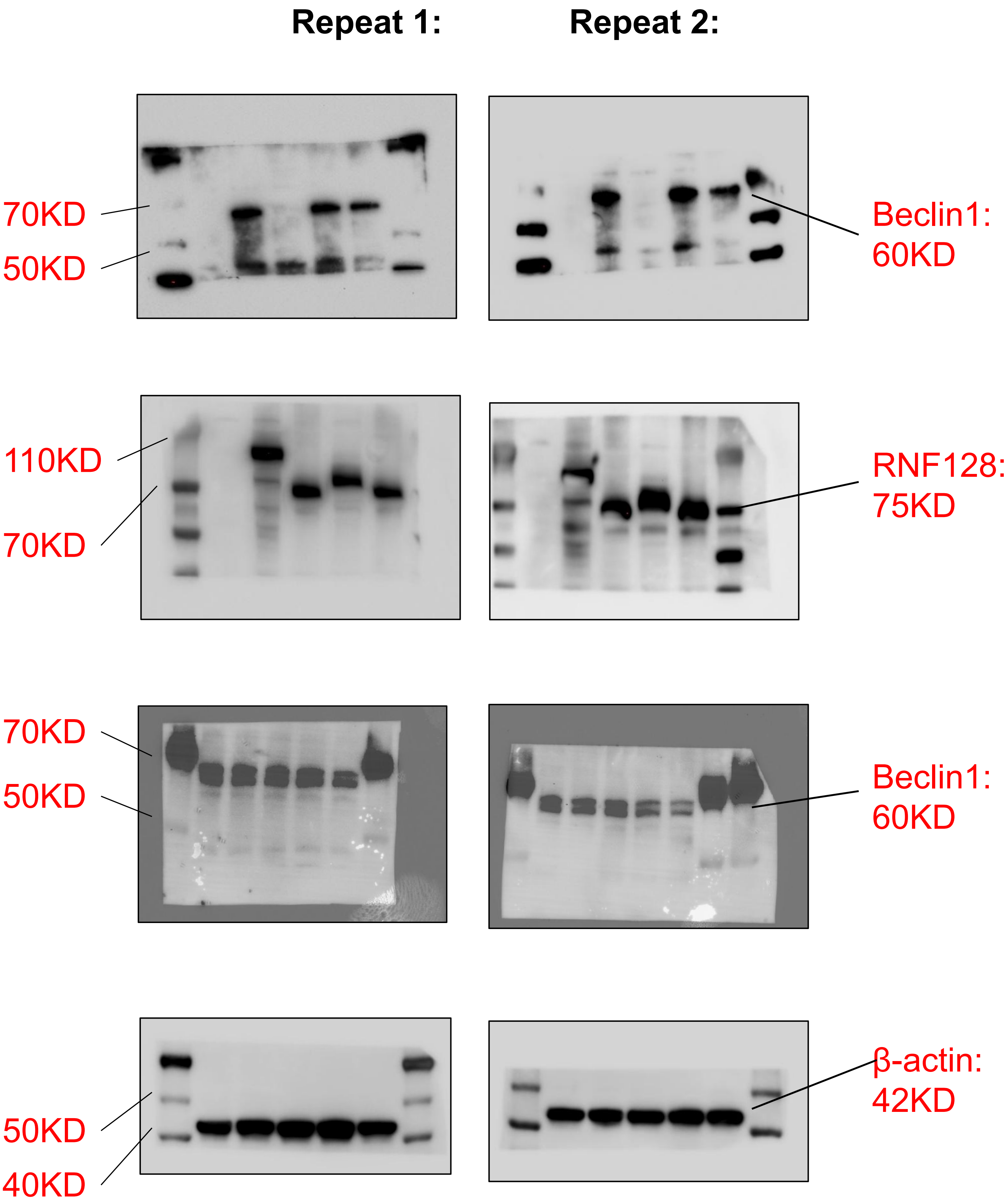
Repeat 1:
Repeat 2:
70KD
50KD
Beclin1:
60KD
110KD
70KD
RNF128:
75KD
70KD
50KD
Beclin1:
60KD
50KD
40KD
β-actin:
42KD

Figure4 h

On fig:

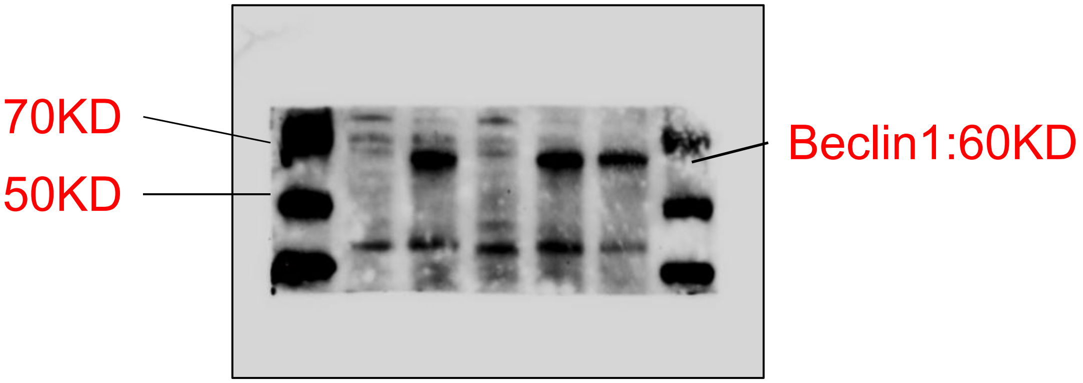

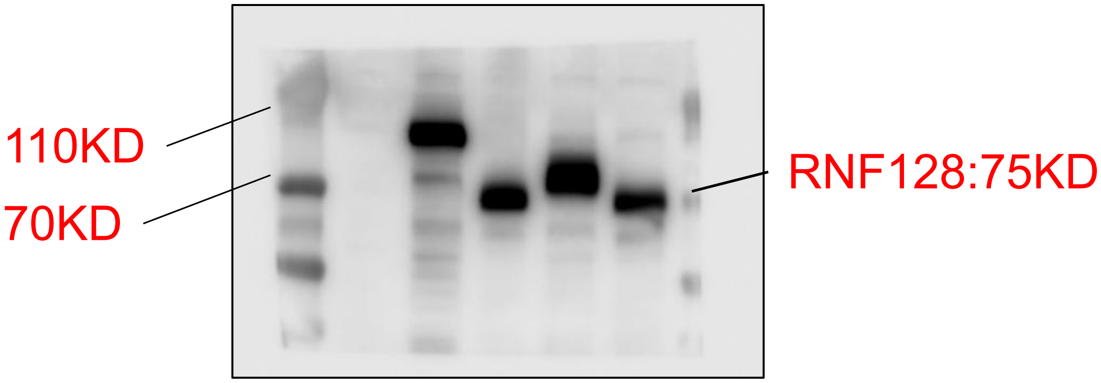

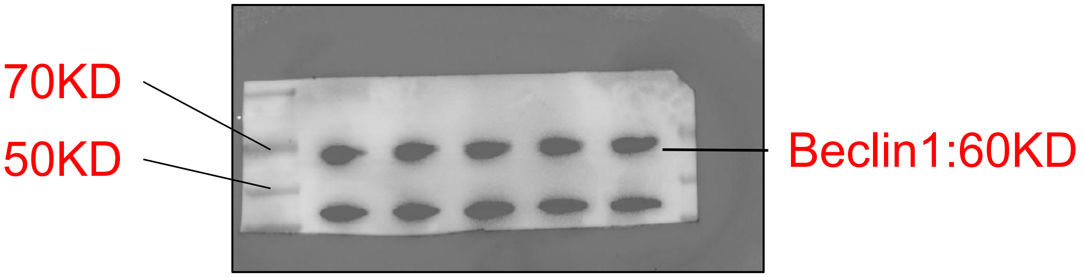

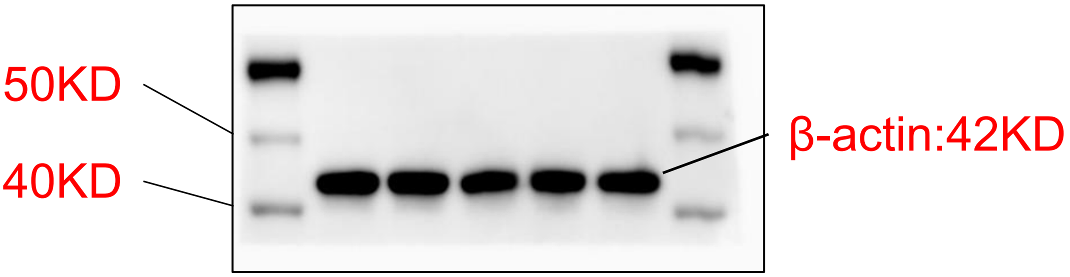

Figure4 h

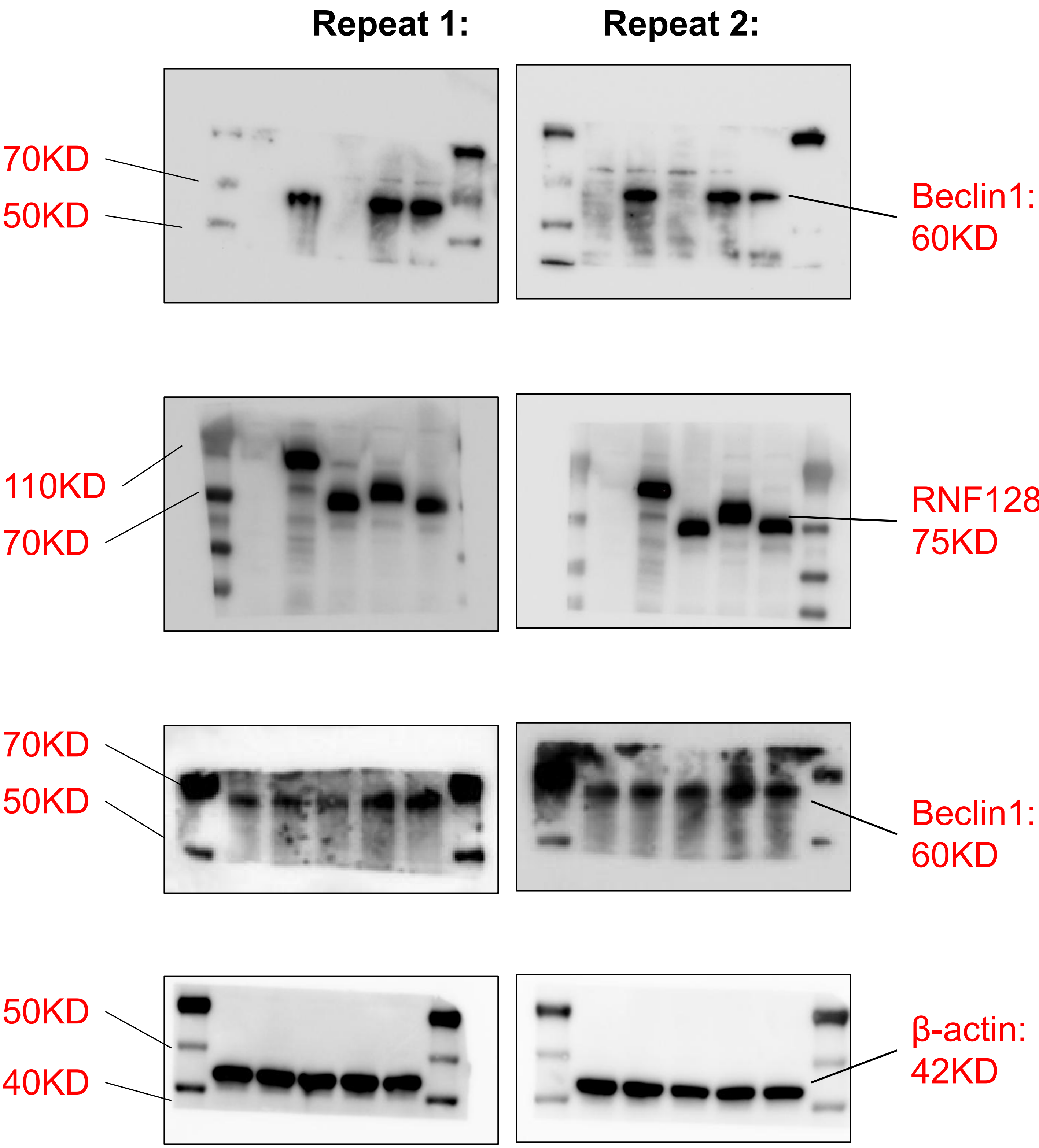

Repeat 1:
Repeat 2:
70KD
50KD
Beclin1:
60KD
110KD
70KD
RNF128
75KD
70KD
50KD
Beclin1:
60KD
50KD
40KD
β-actin:
42KD

Figure5 c

On fig:

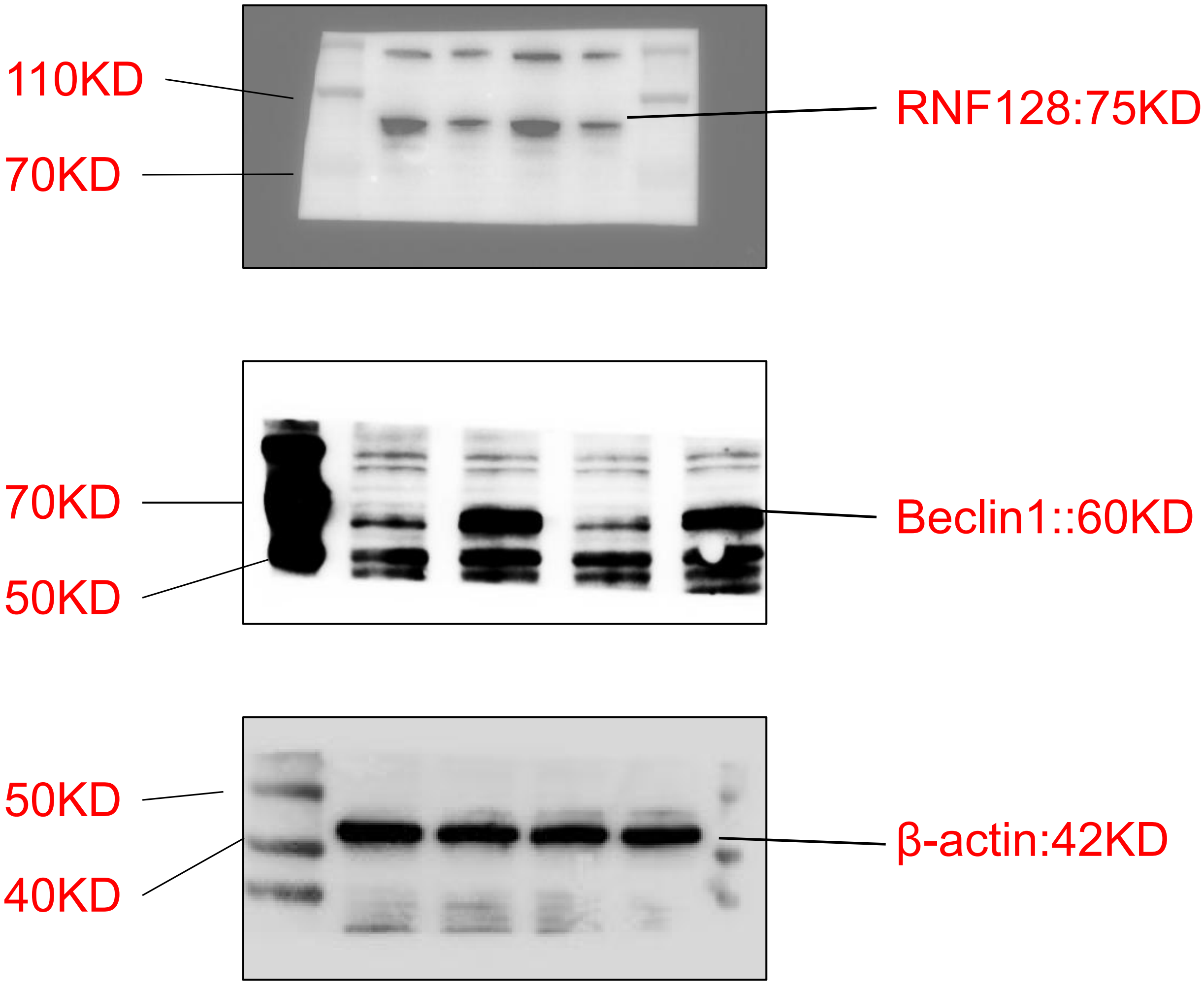

Figure5 c

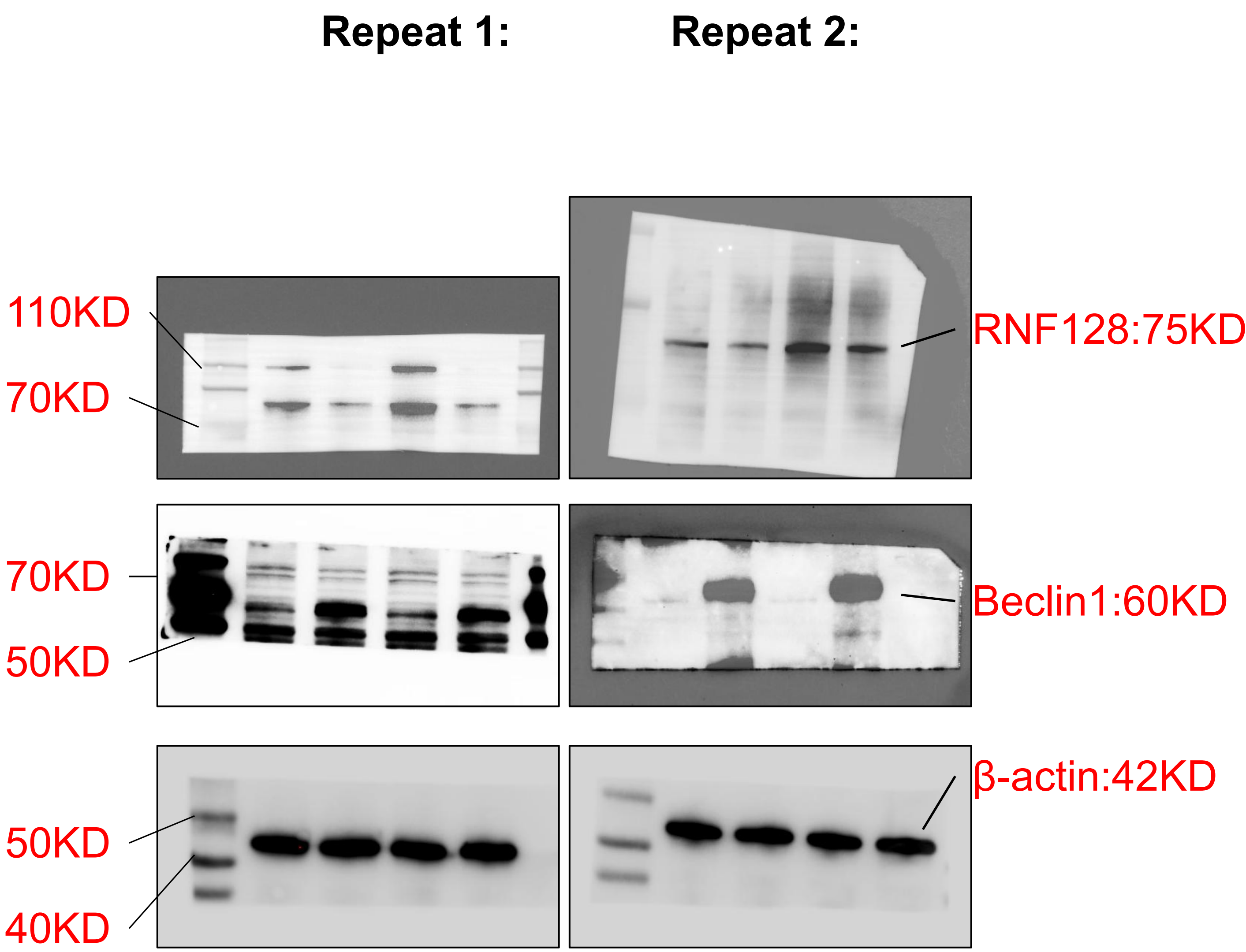

Repeat 1:
Repeat 2:
110KD
70KD
RNF128:75KD
70KD
50KD
Beclin1:60KD
50KD
40KD
β-actin:42KD

Figure5 d

On fig:

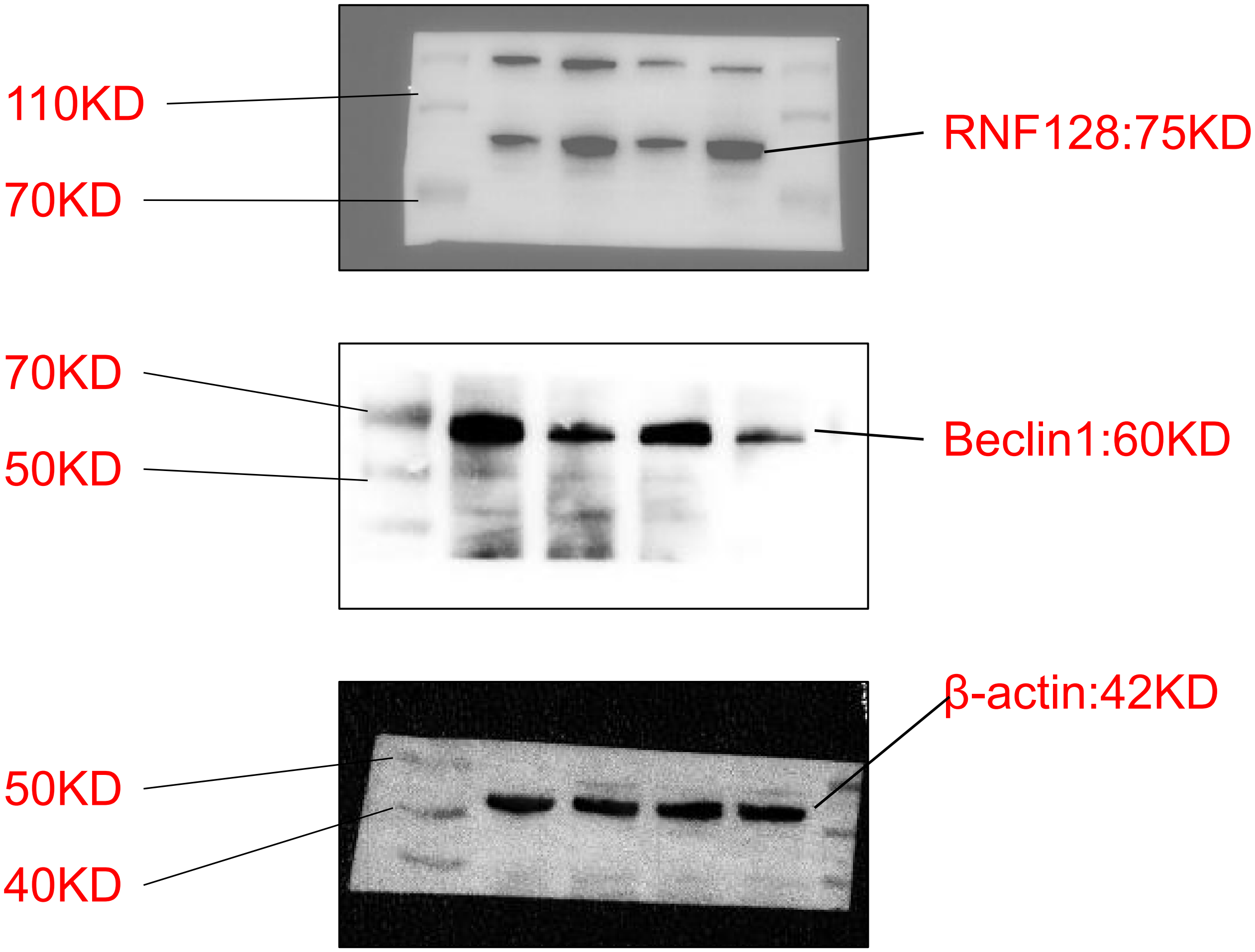

Figure5 d

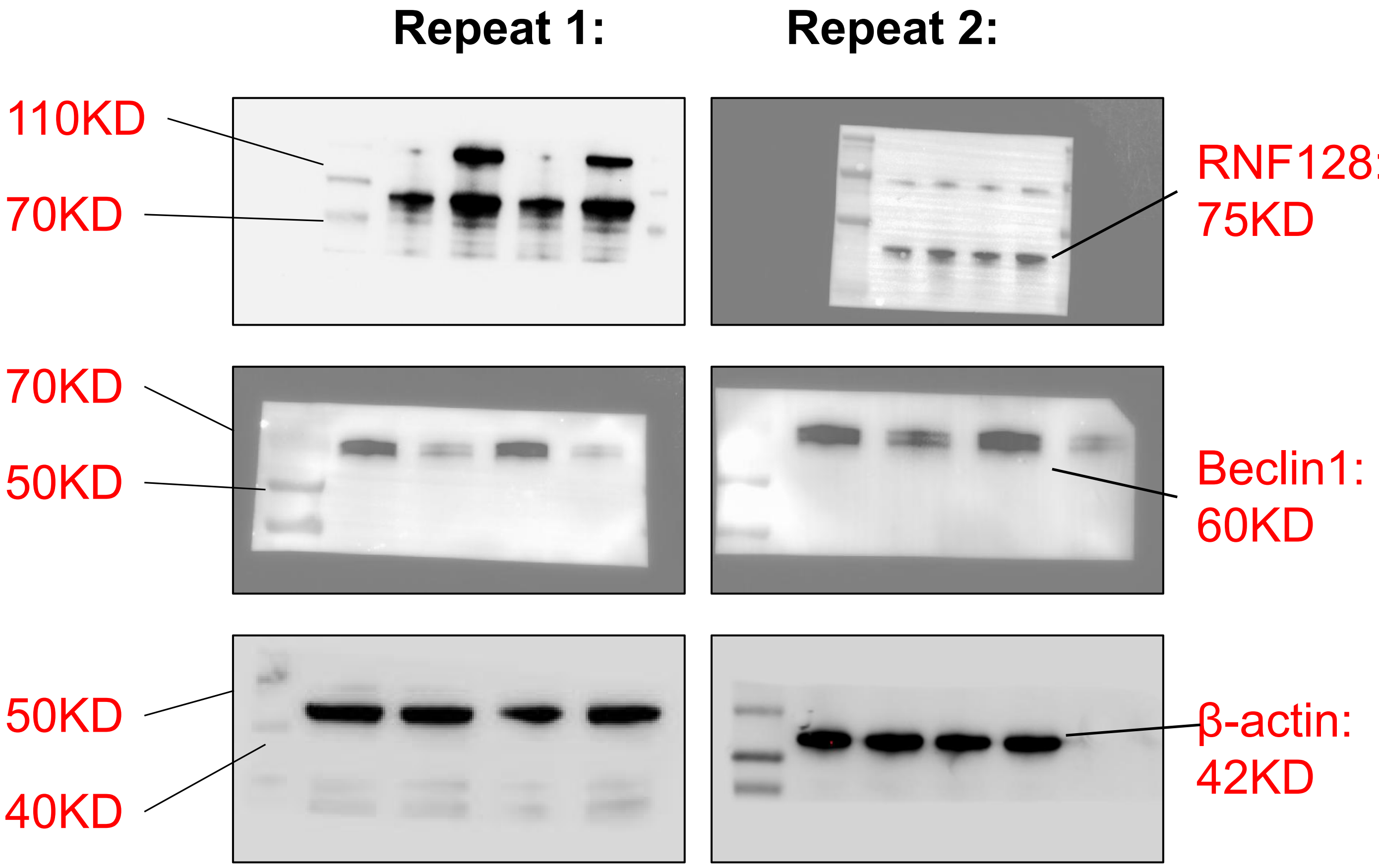
Repeat 1:
Repeat 2:
110KD
70KD
RNF128:
75KD
70KD
50KD
Beclin1:
60KD
50KD
40KD
β-actin:
42KD

Figure5 e

On fig:

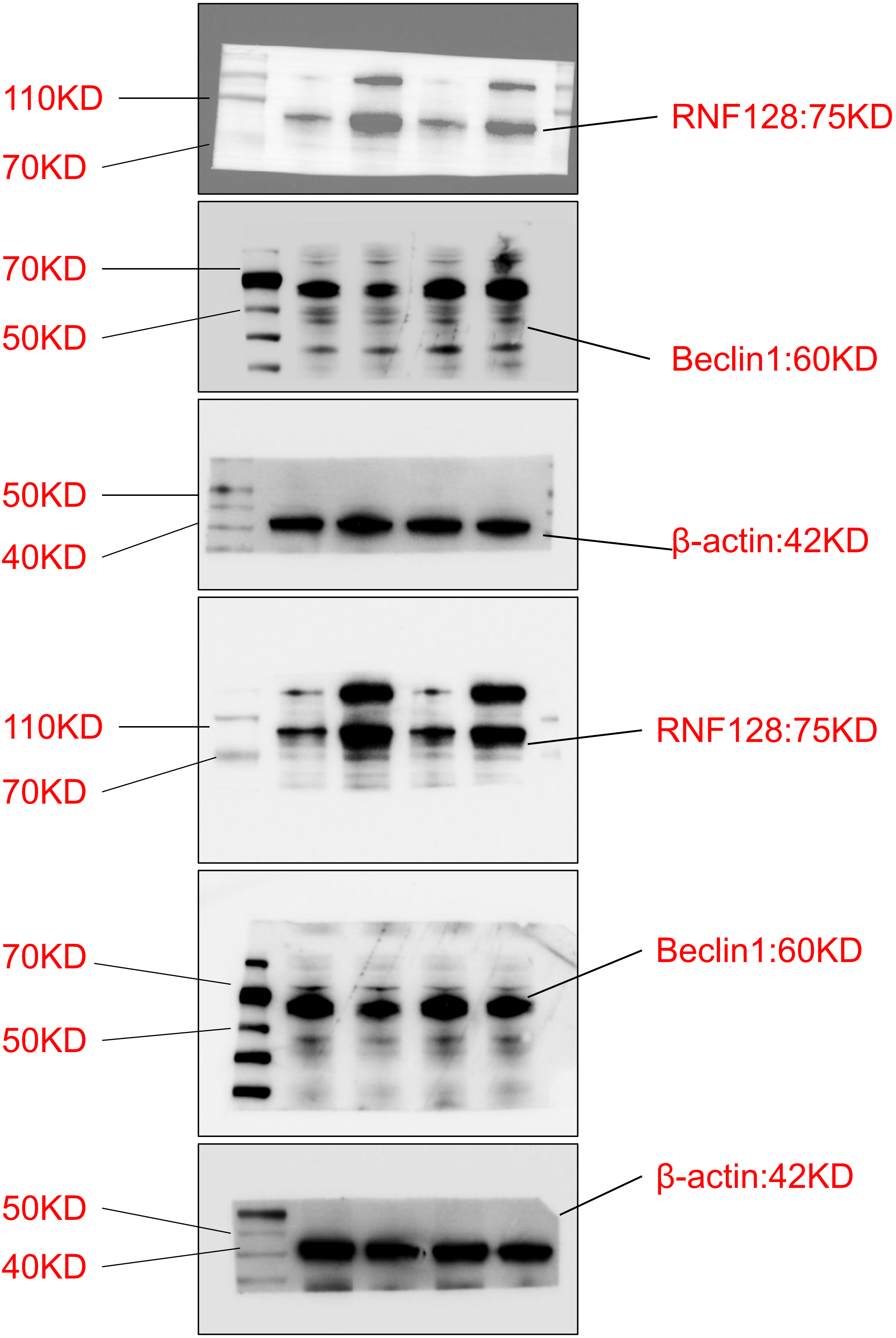

Figure5 e

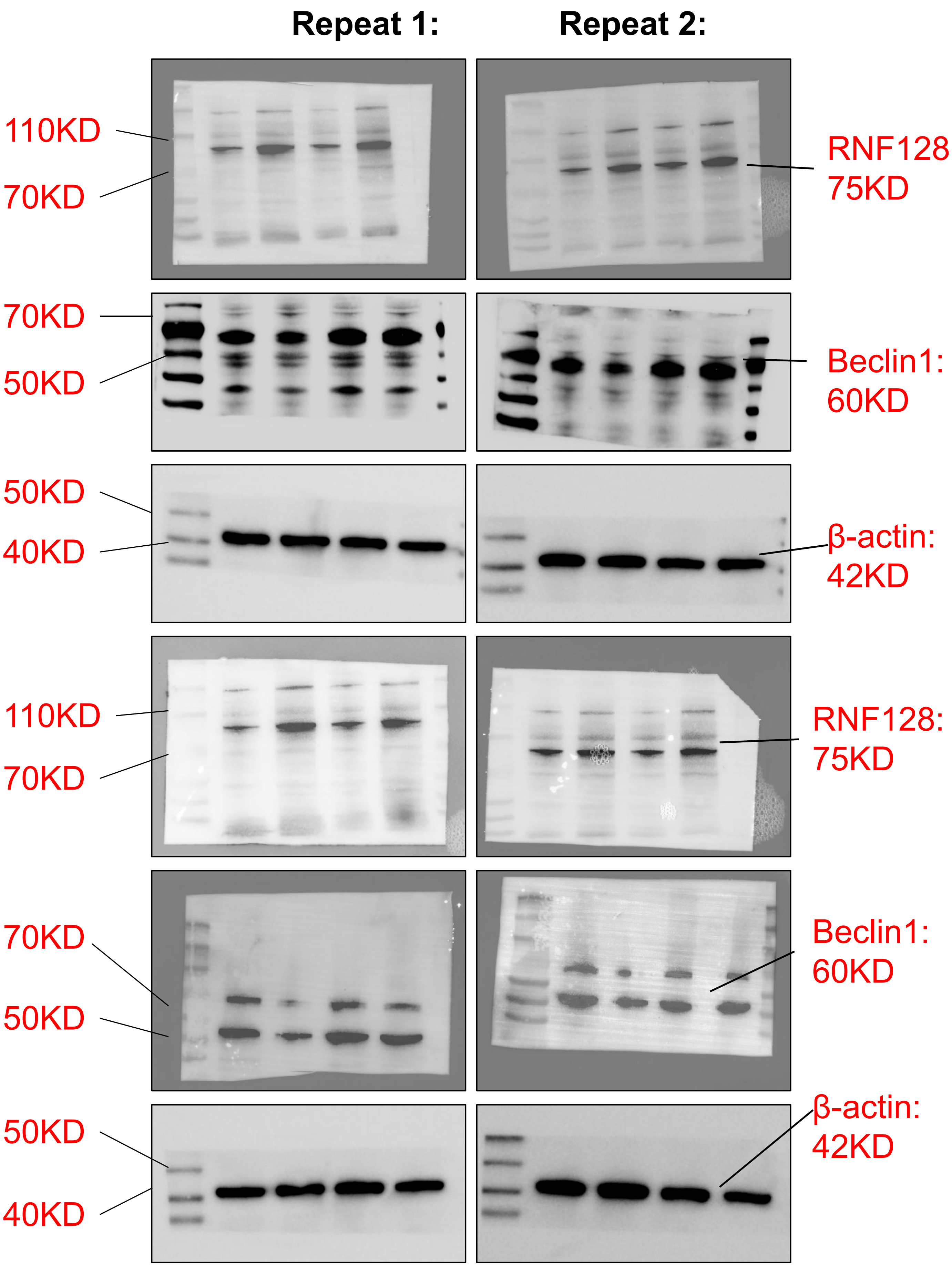

Repeat 1:
Repeat 2:
110KD
70KD
RNF128
75KD
70KD
50KD
Beclin1:
60KD
50KD
40KD
β-actin:
42KD
110KD
70KD
RNF128:
75KD
70KD
50KD
Beclin1:
60KD
50KD
40KD
β-actin:
42KD

Figure5 f

on fig:

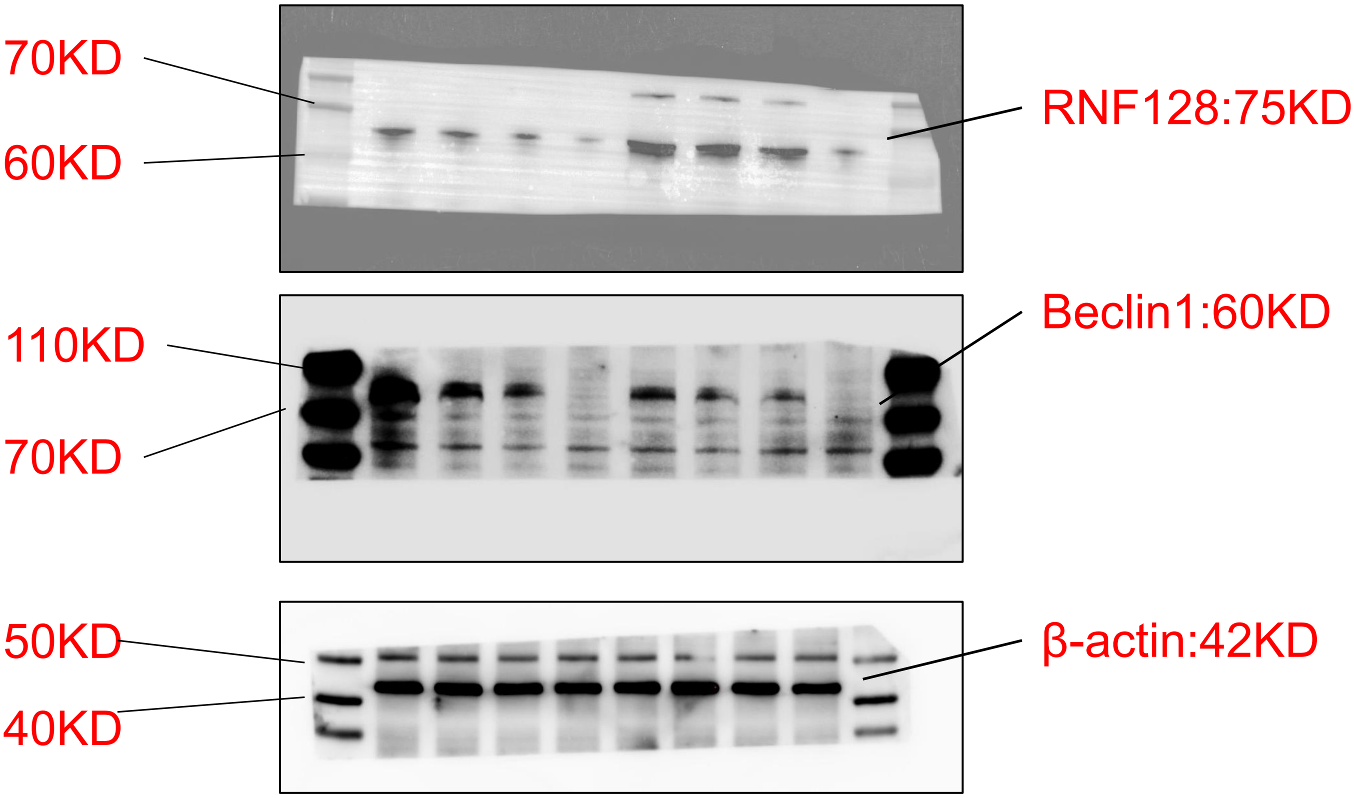

Figure5 f

Repeat 1:

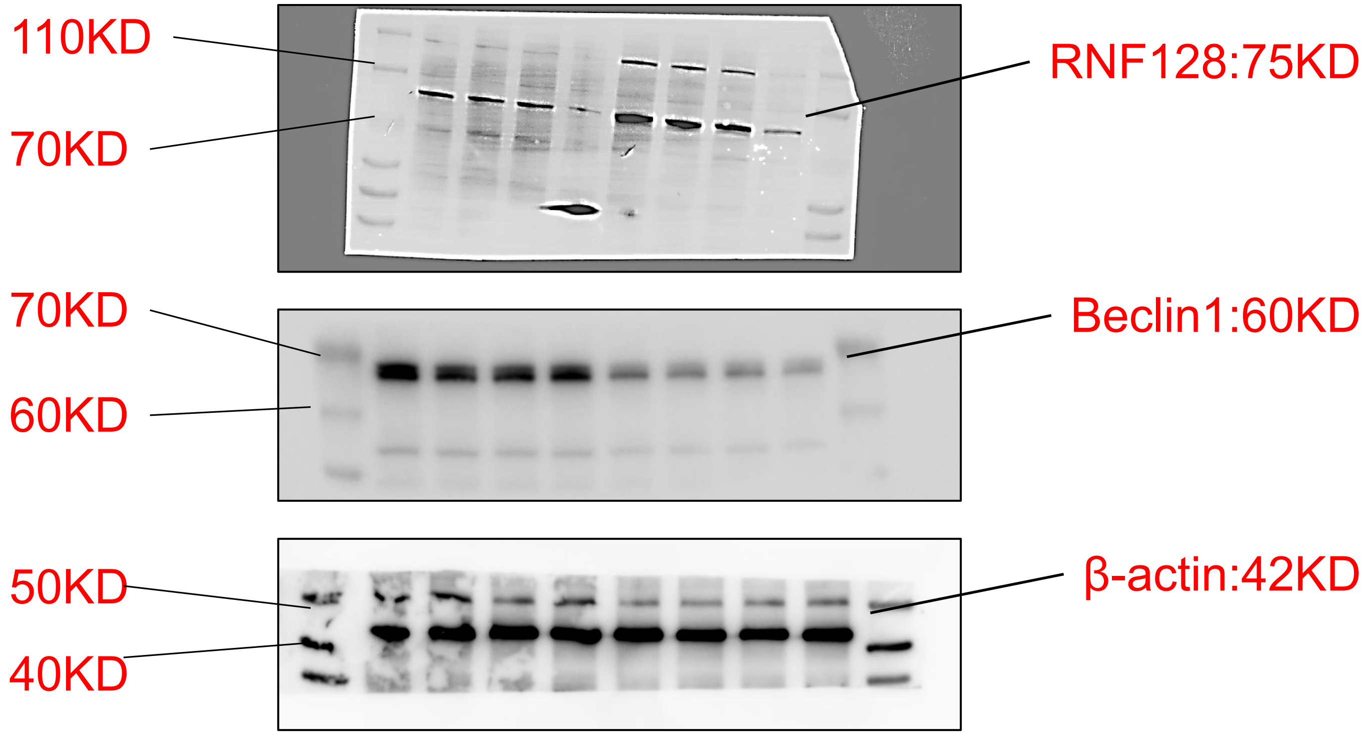

Repeat 2:

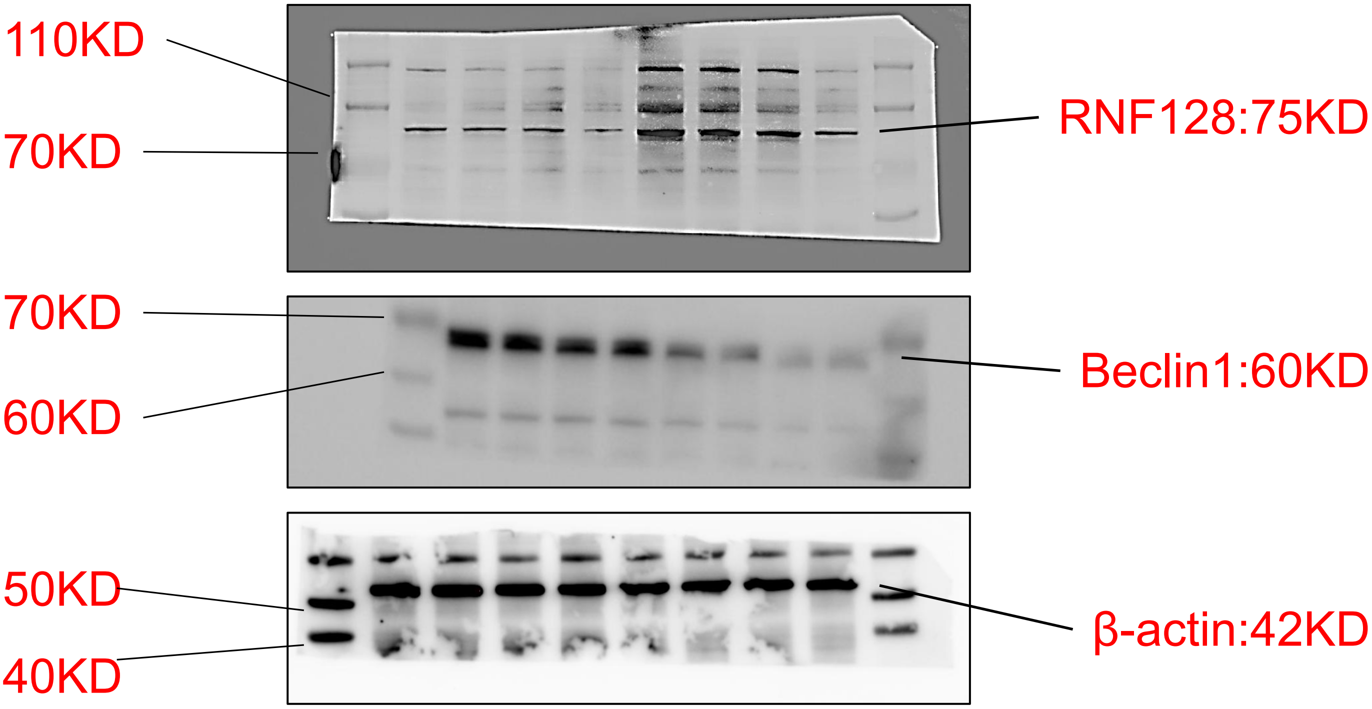

Figure5 g

AGS on fig:

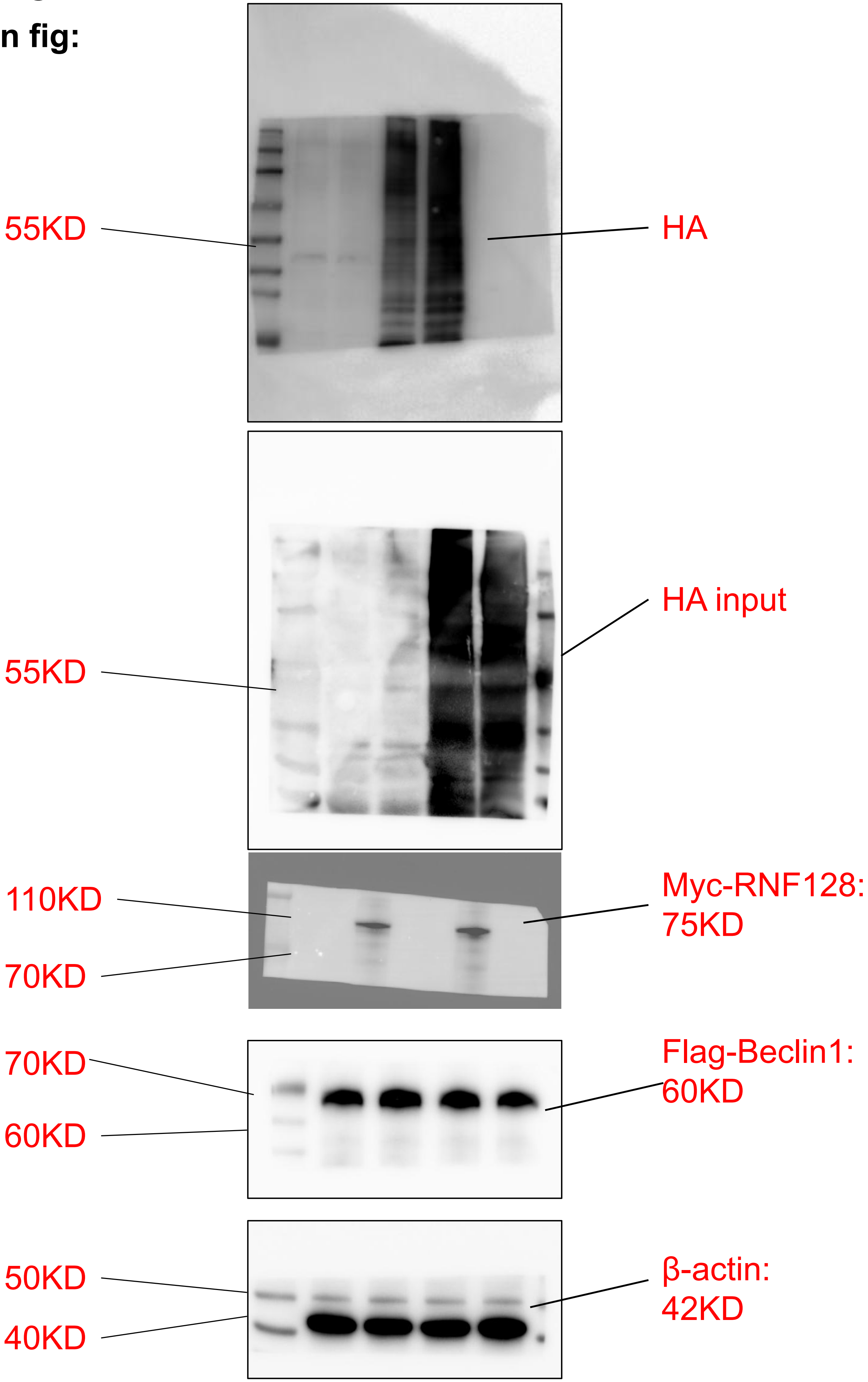

Figure5 g

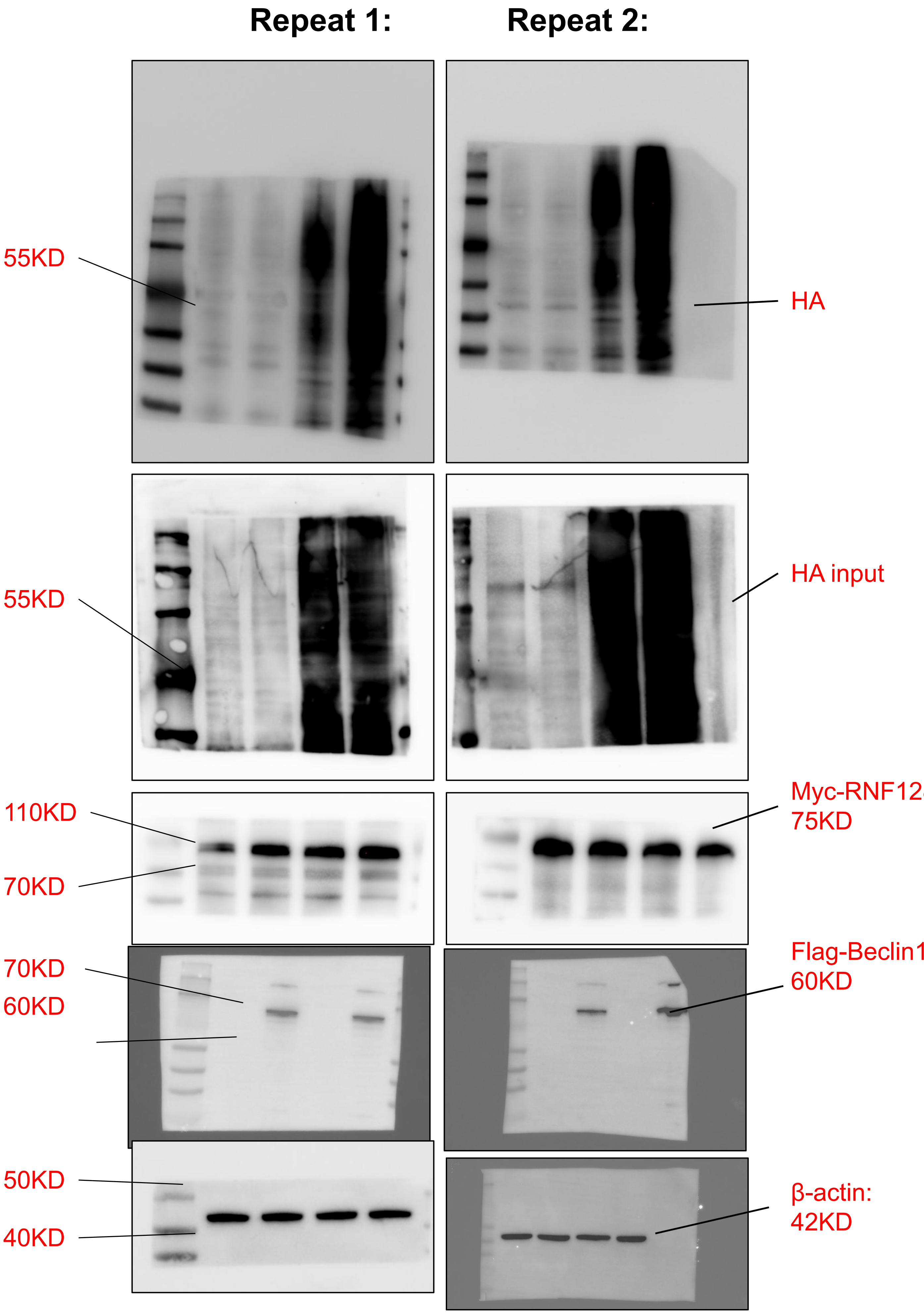

Repeat 1:
Repeat 2:
55KD
HA
55KD
HA input
110KD
70KD
Myc-RNF12
75KD
70KD
60KD
Flag-Beclin1
60KD
50KD
40KD
β-actin:
42KD

Figure5 g

HGC-27 on fig:

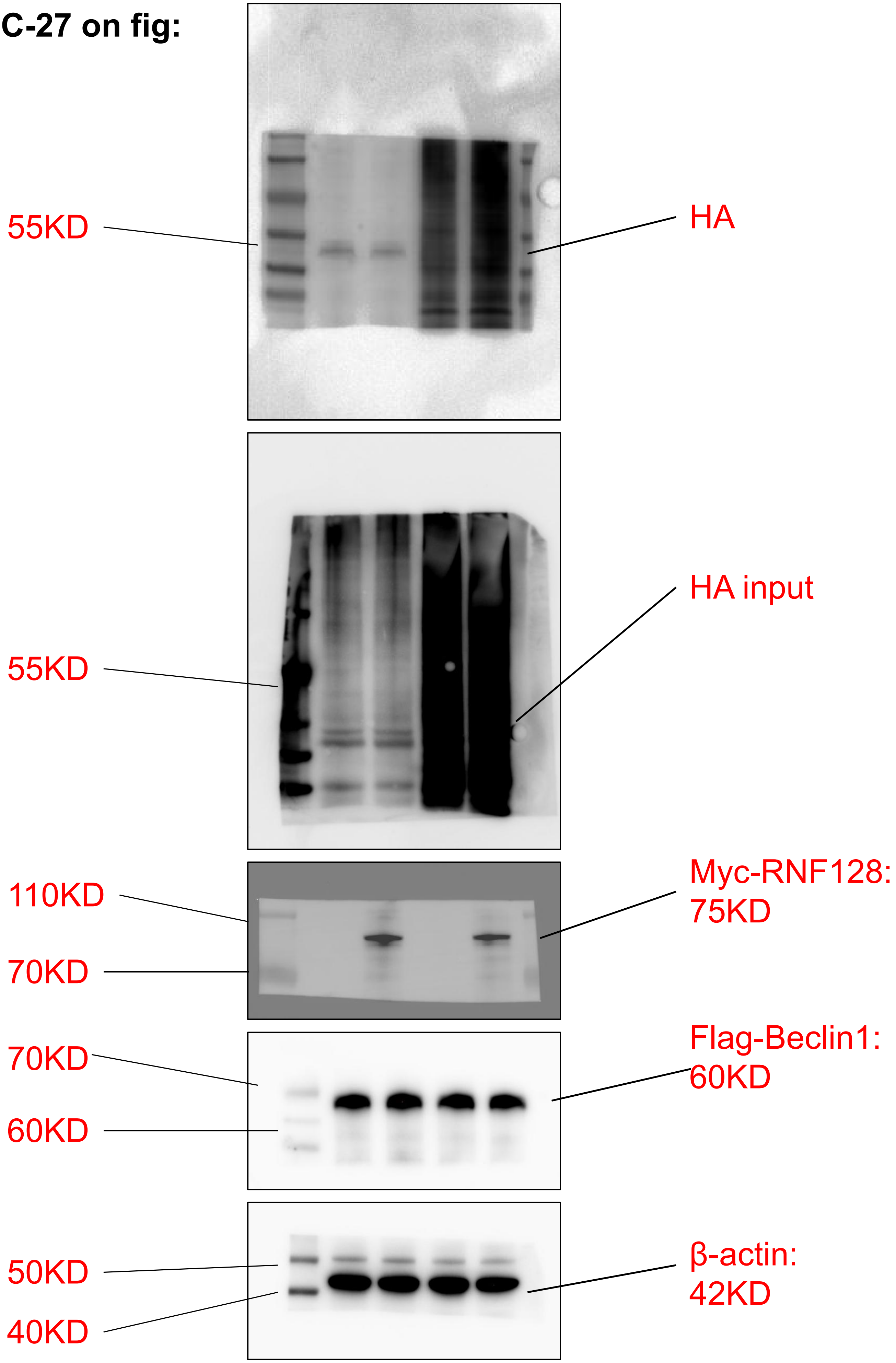

Figure5 g

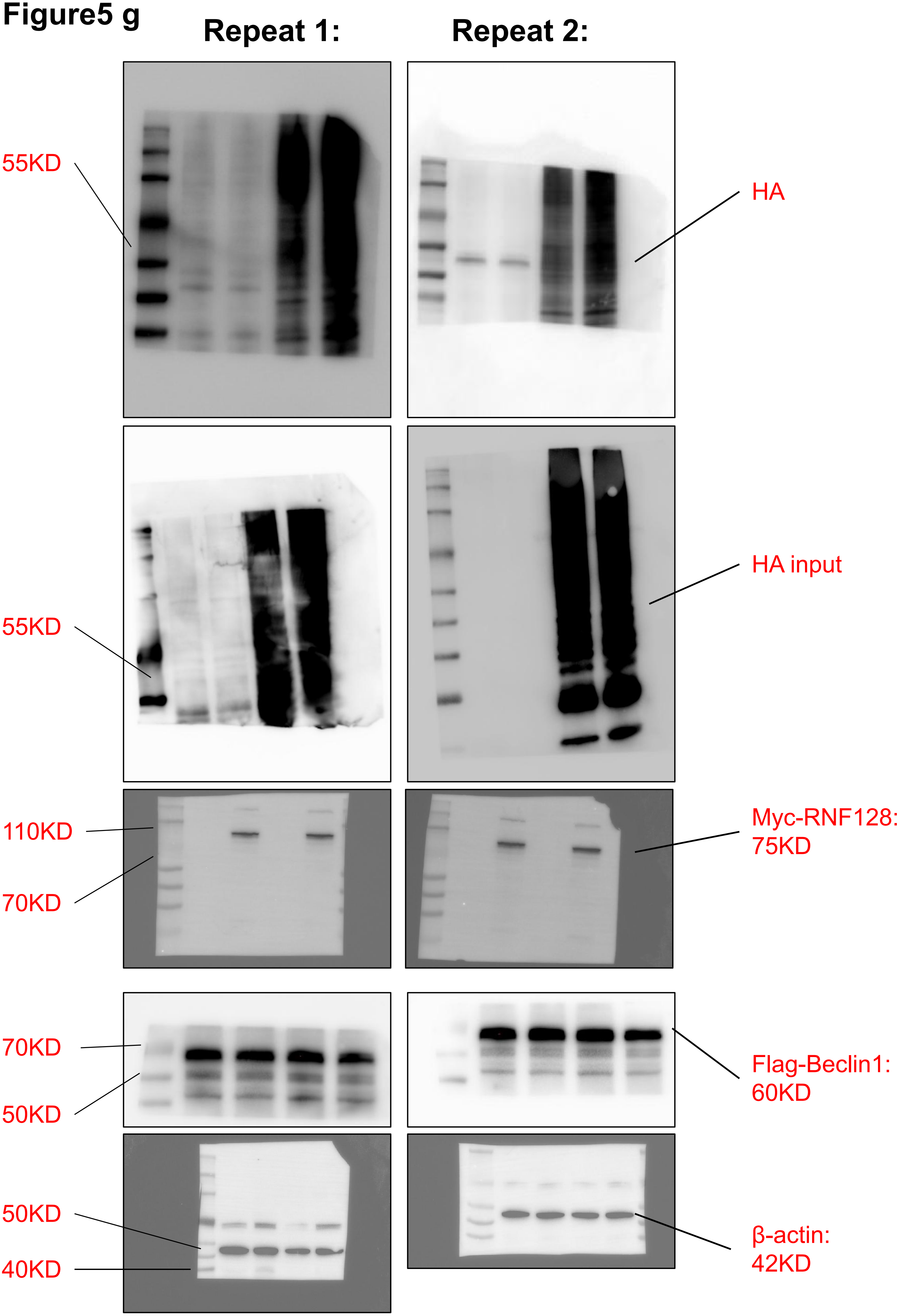
Repeat 1:
Repeat 2:
55KD
HA
55KD
HA input
110KD
70KD
Myc-RNF128:
75KD
70KD
50KD
Flag-Beclin1:
60KD
50KD
40KD
β-actin:
42KD

Figure6 a

On fig:

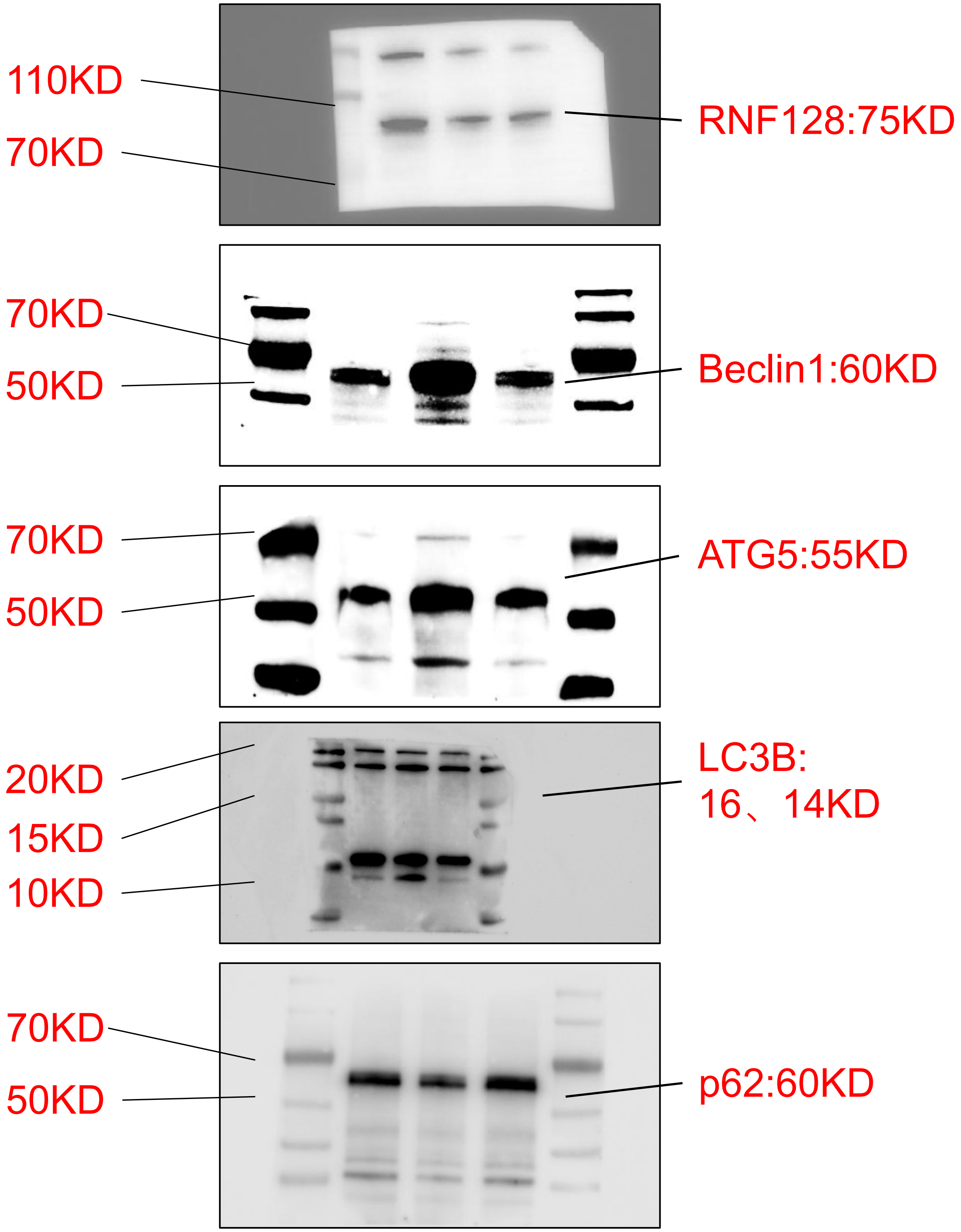

Figure6 a

On fig:

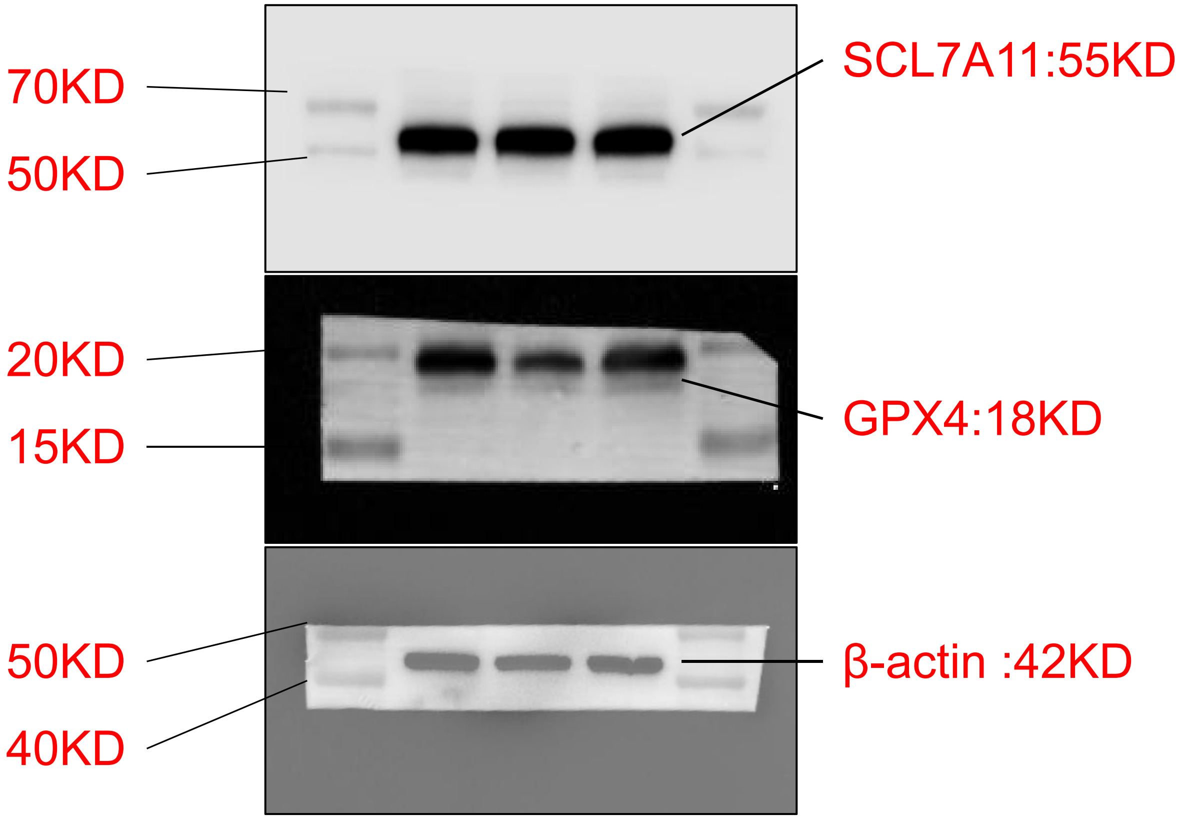

Figure6 a

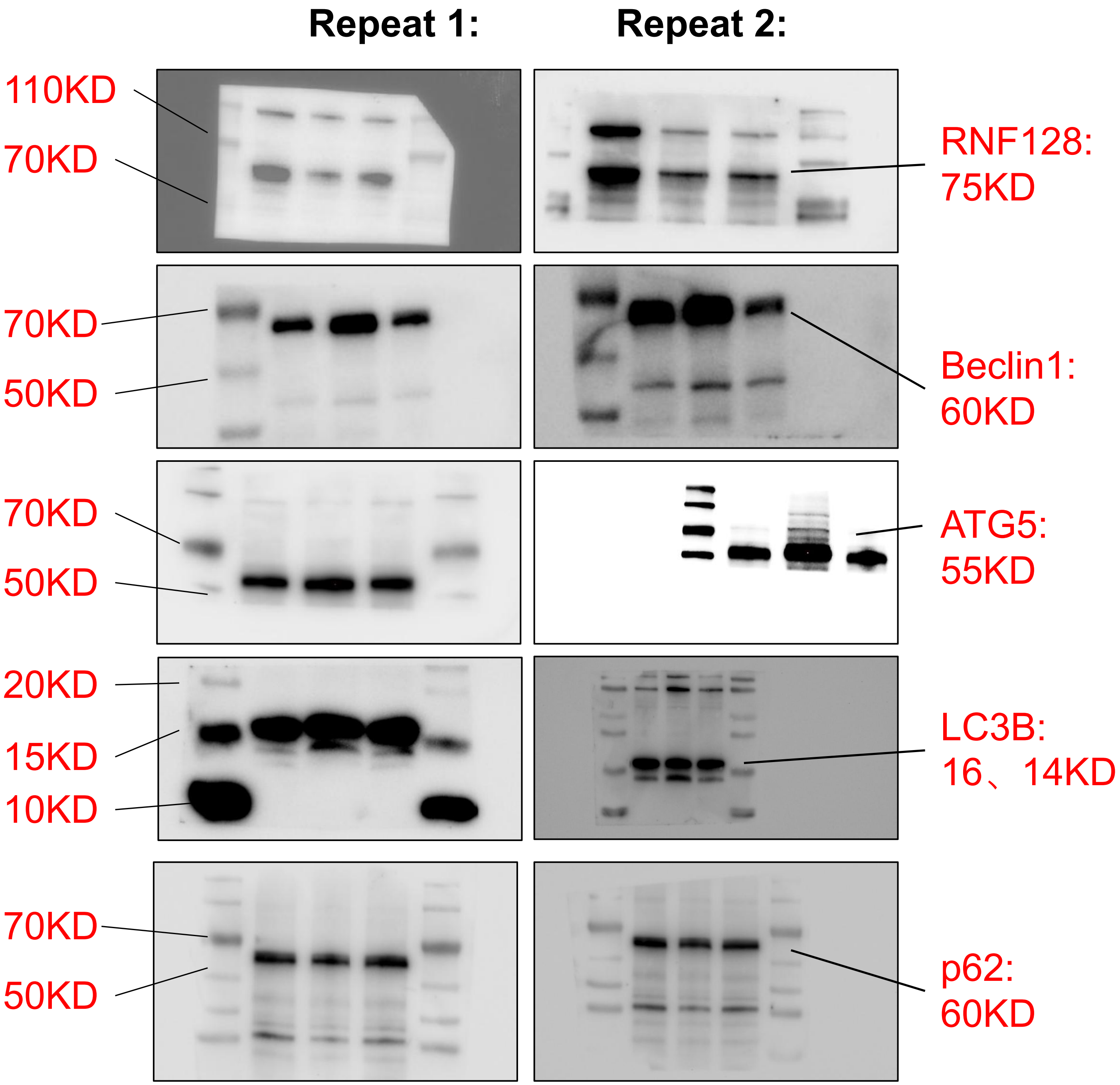

Repeat 1:
Repeat 2:
110KD
70KD
RNF128:
75KD
70KD
50KD
Beclin1:
60KD
70KD
50KD
ATG5:
55KD
20KD
15KD
10KD
LC3B:
16、14KD
70KD
50KD
p62:
60KD

Figure6 a

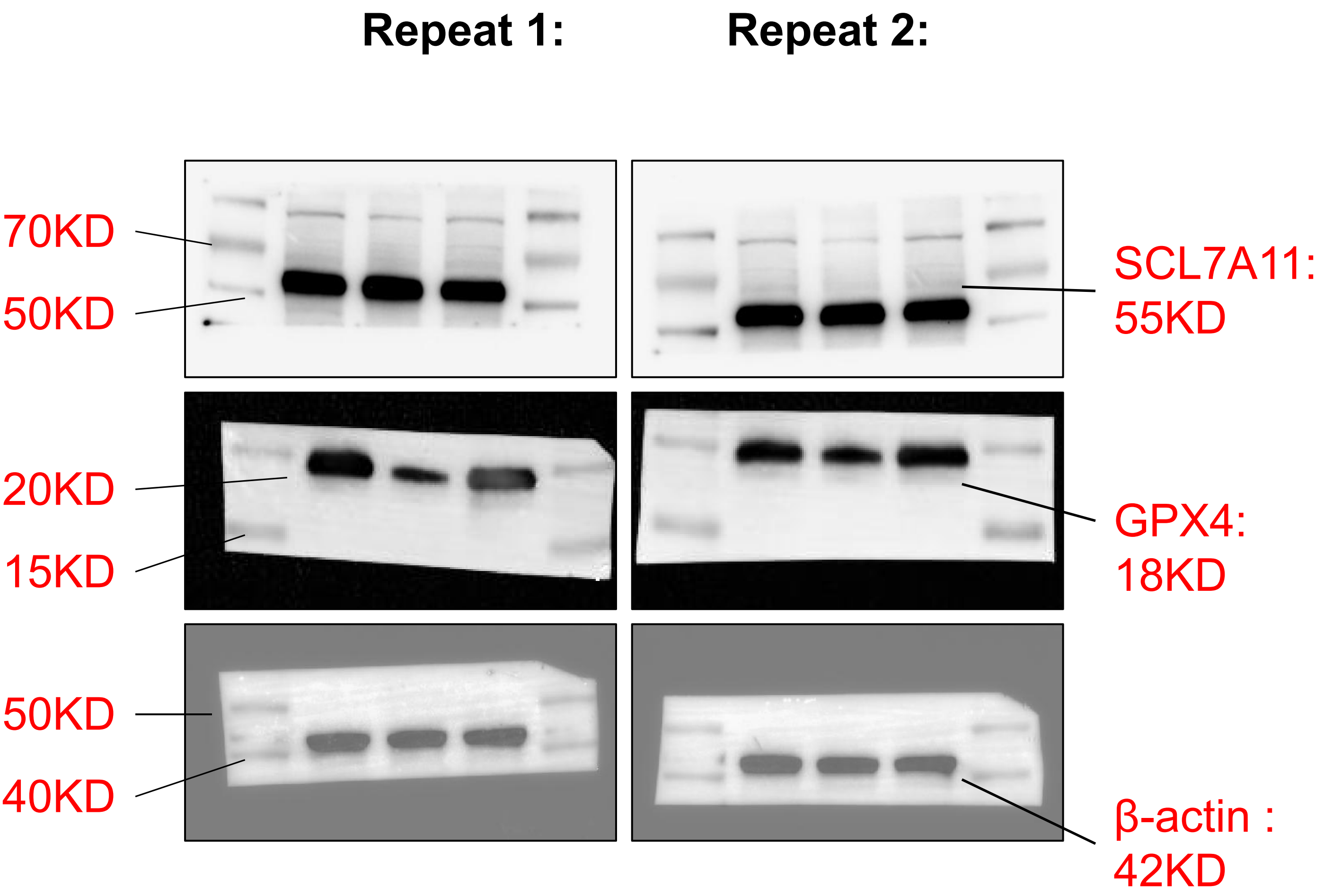

Repeat 1:
Repeat 2:
70KD
50KD
SCL7A11:
55KD
20KD
15KD
GPX4:
18KD
50KD
40KD
β-actin :
42KD

Figure6 a

On fig:

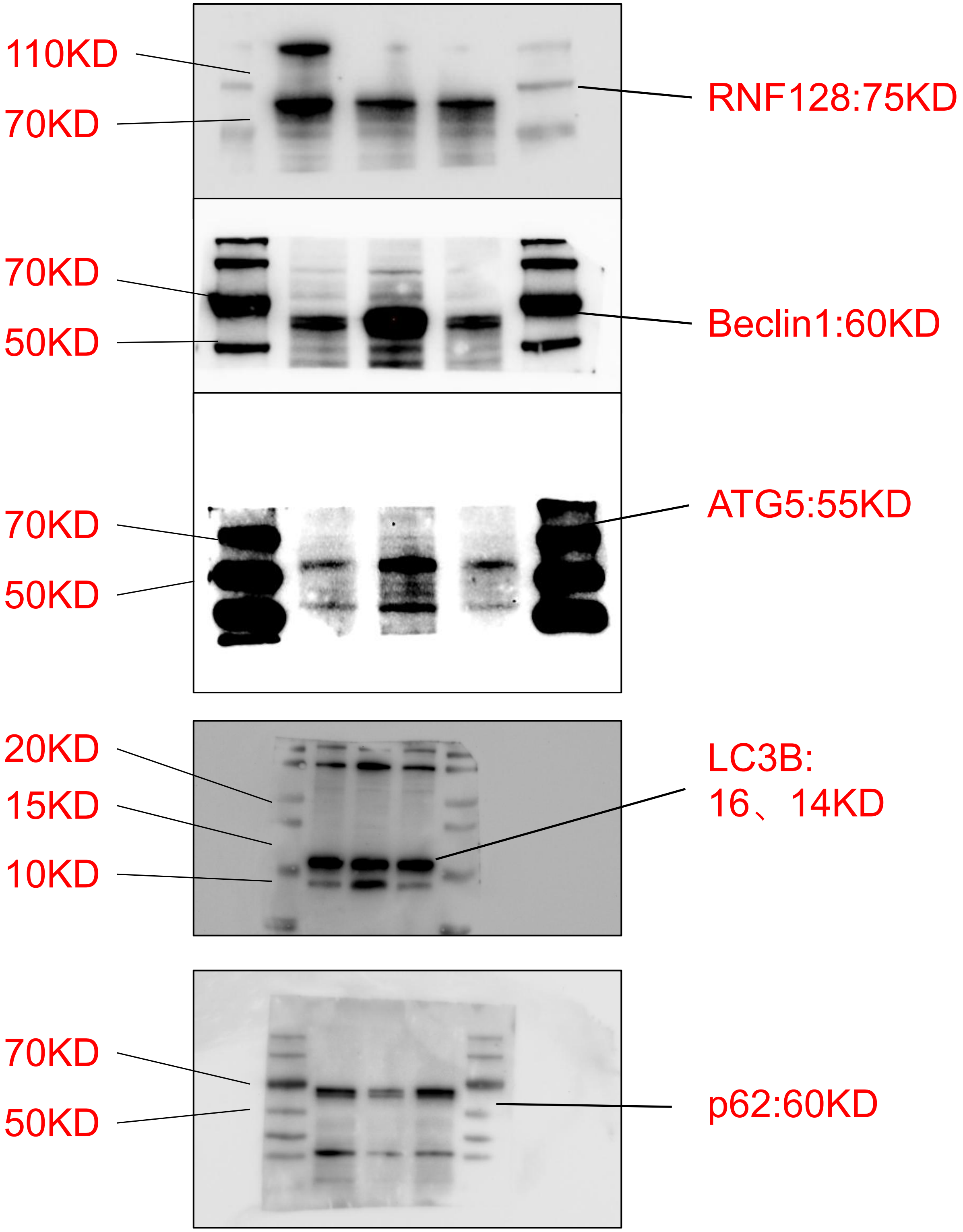

On fig:

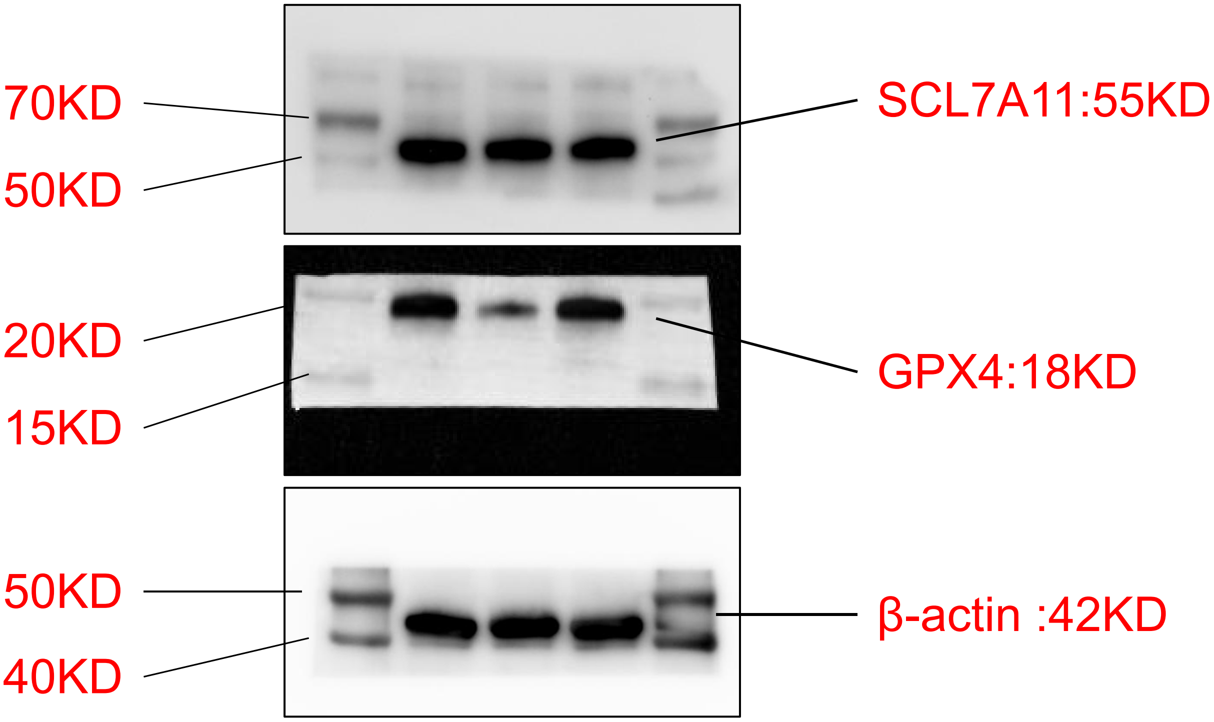
70KD
50KD
SCL7A11:55KD
20KD
15KD
GPX4:18KD
50KD
40KD
β-actin :42KD

Figure6 a

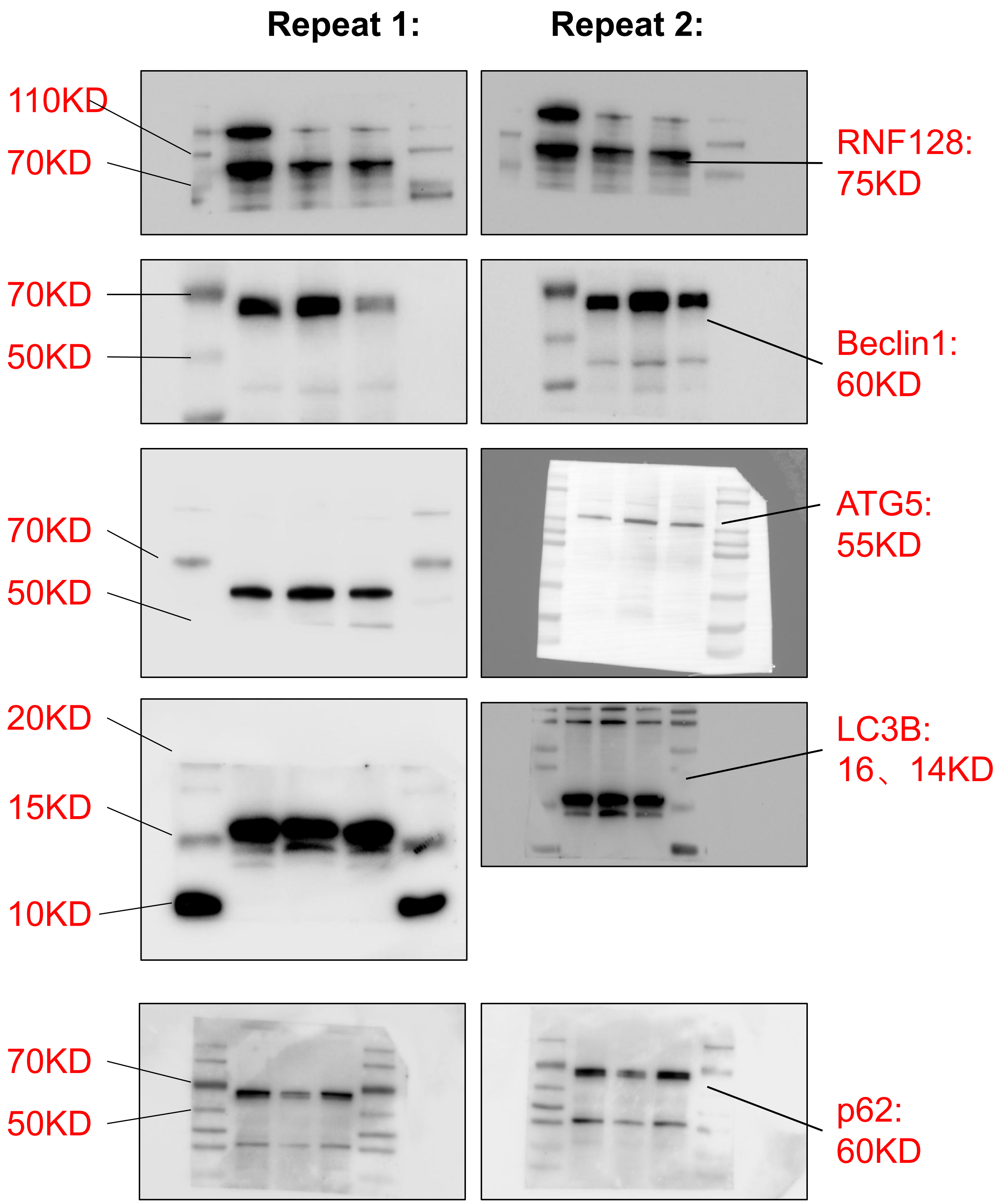

Repeat 1:
Repeat 2:
110KD
70KD
RNF128:
75KD
70KD
50KD
Beclin1:
60KD
70KD
50KD
ATG5:
55KD
20KD
15KD
10KD
LC3B:
16、14KD
70KD
50KD
p62:
60KD

Figure6 a

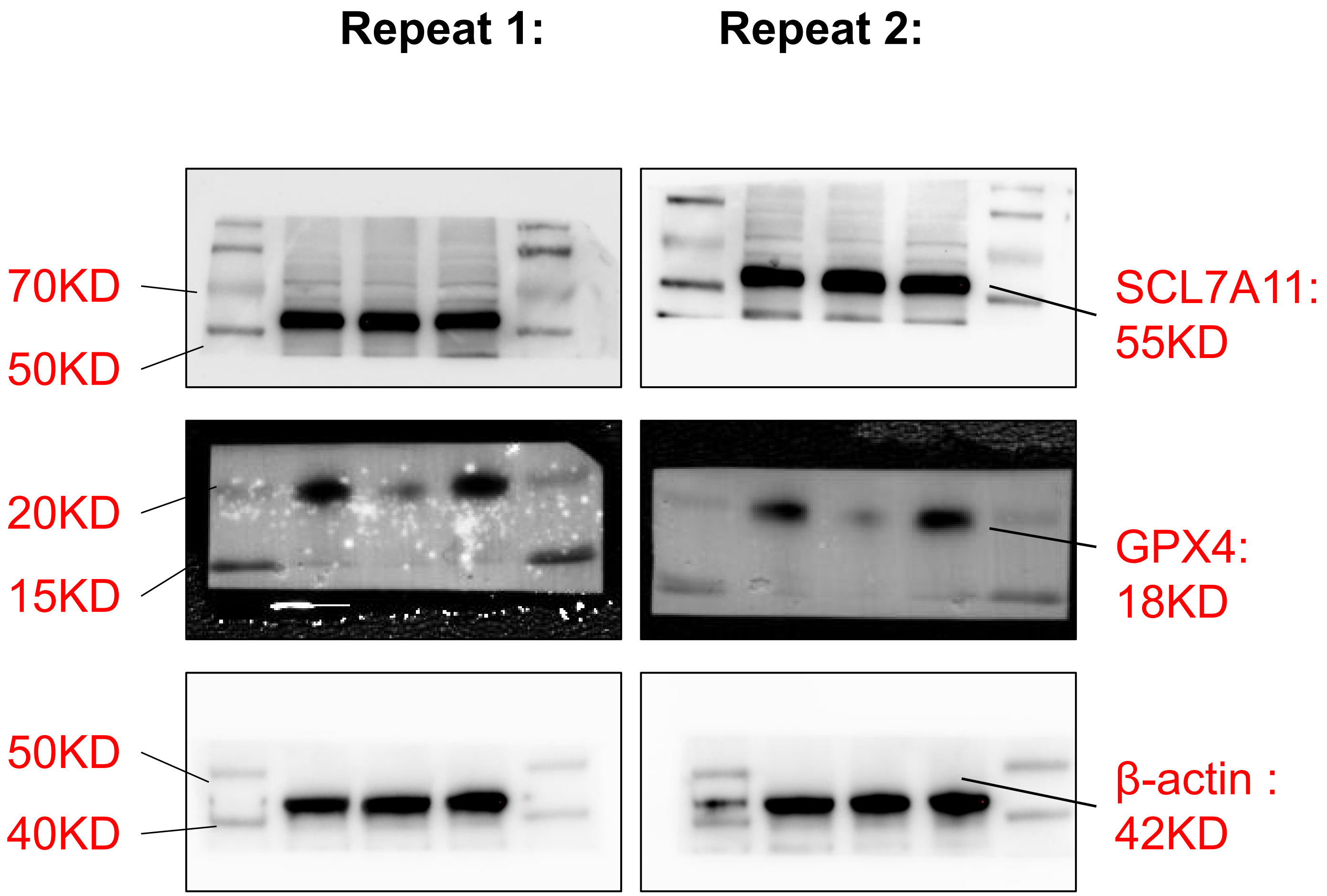

Repeat 1:
Repeat 2:
70KD
50KD
SCL7A11:
55KD
20KD
15KD
GPX4:
18KD
50KD
40KD
β-actin :
42KD

Figure S1 a

On fig:

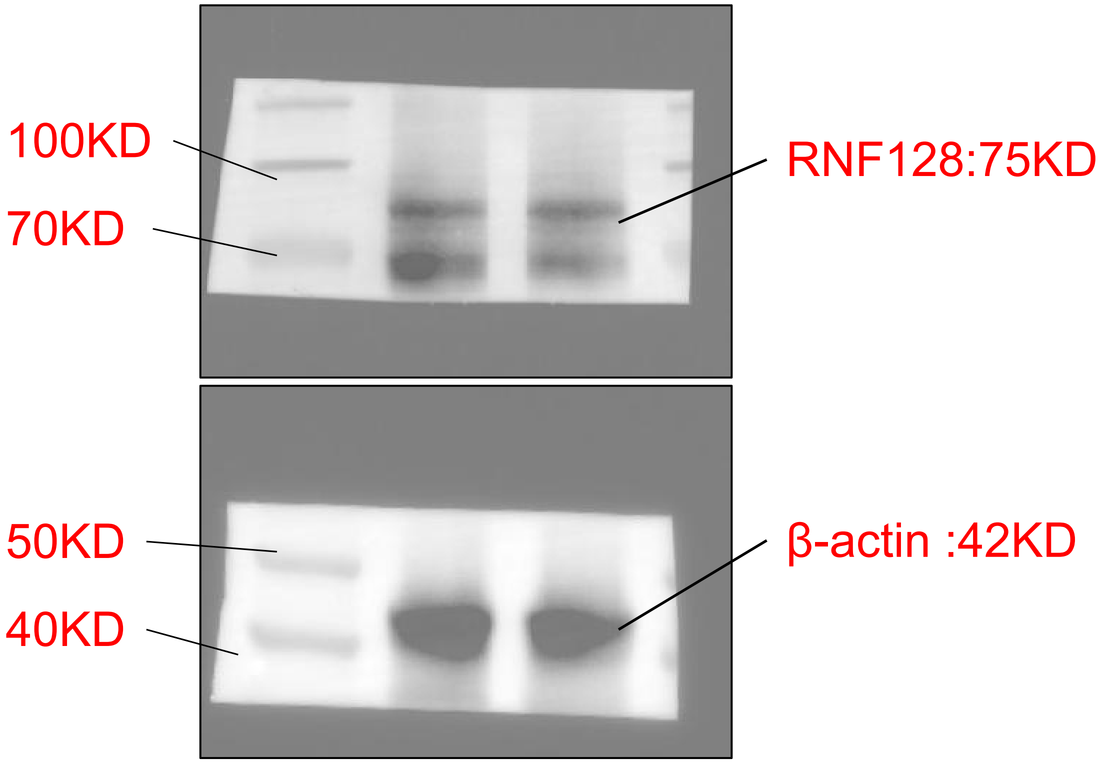

Repeat 1:

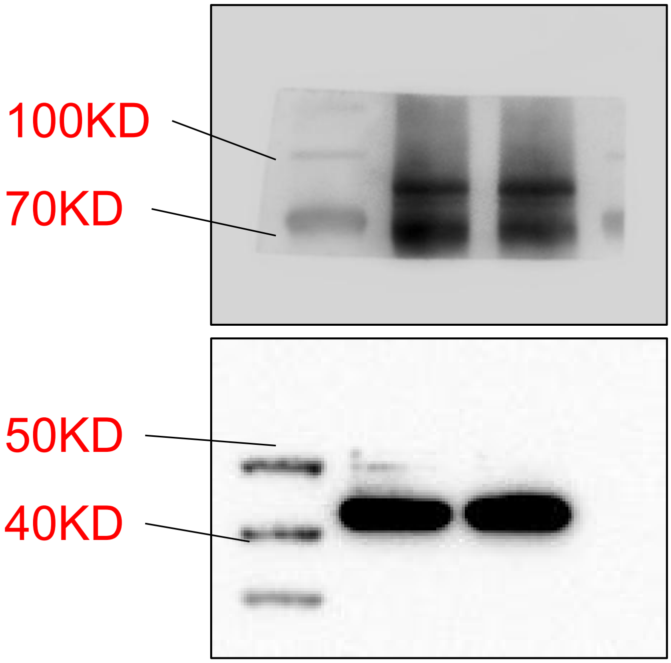

Repeat 2:

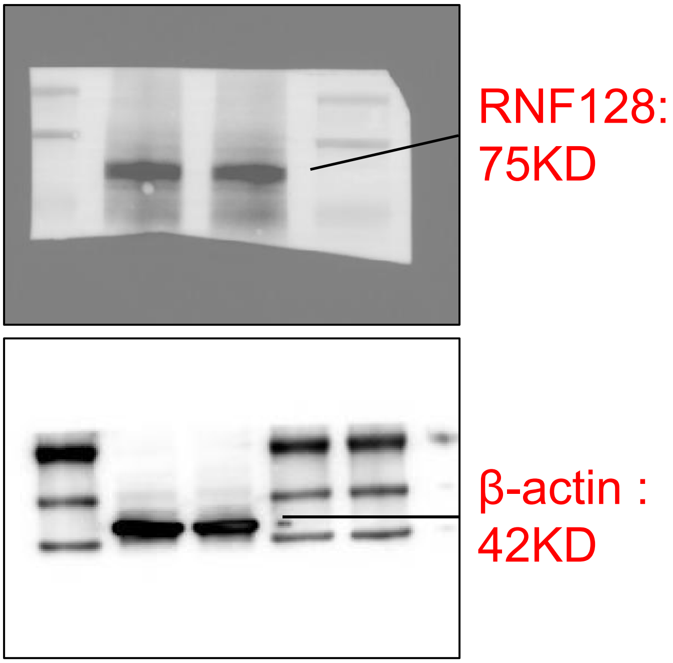

Figure S1 b

On fig:

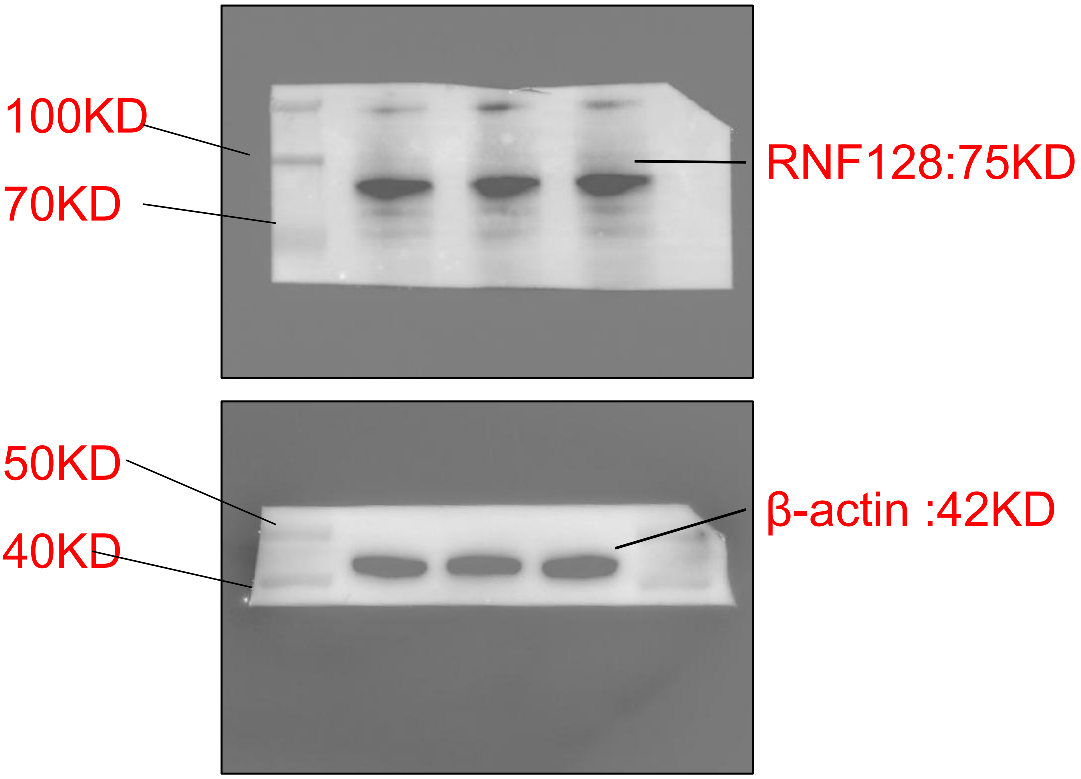

Repeat 1: **Repeat 2:**

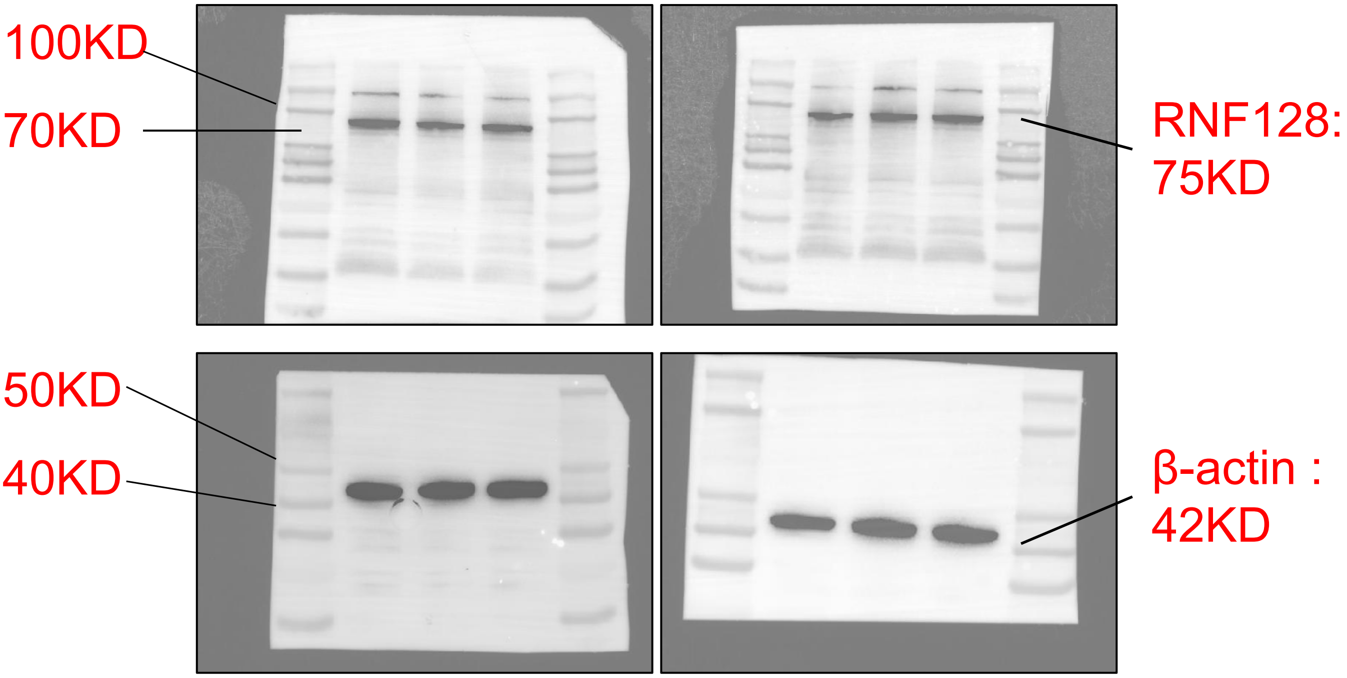

Figure S2 c

AGS On fig:

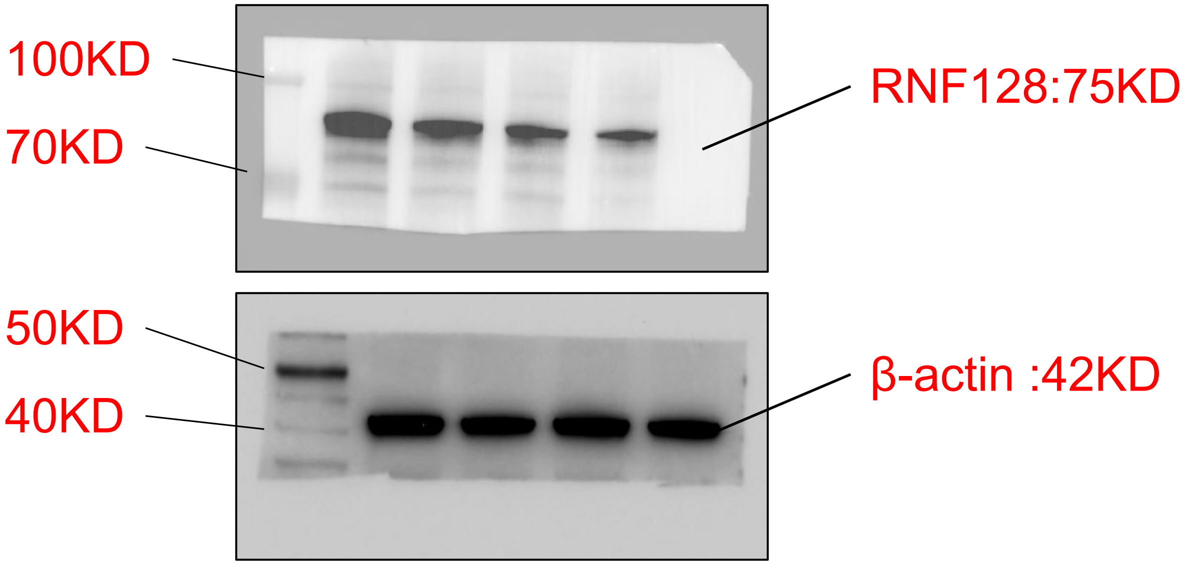

Repeat 1: **Repeat 2:**

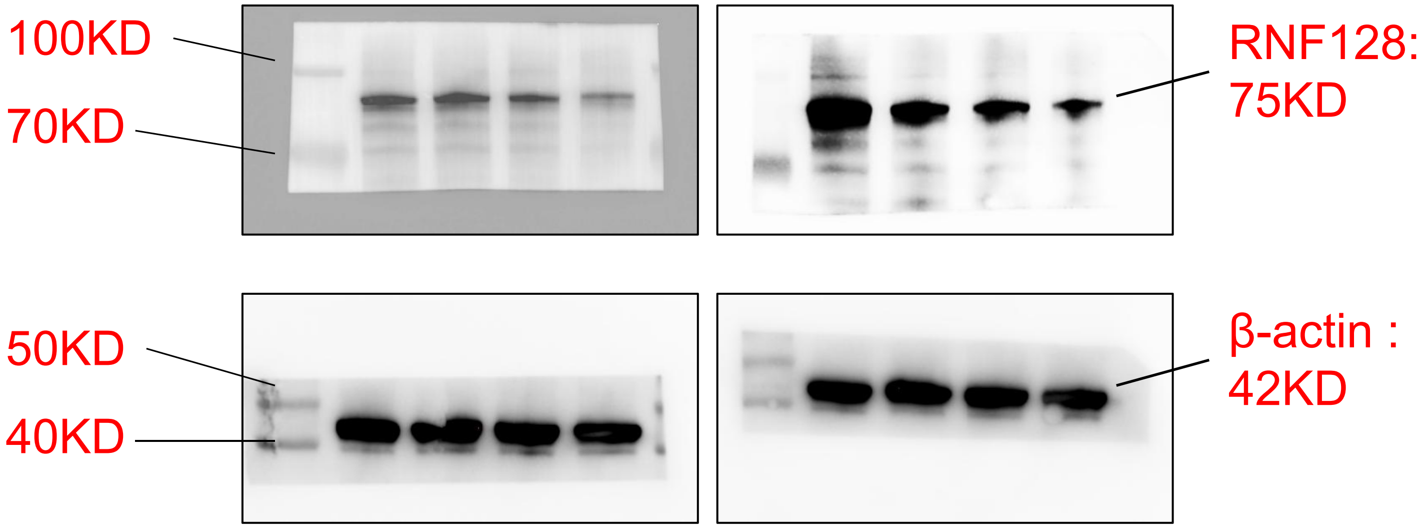

Figure S2 c

HGC-27 On fig:

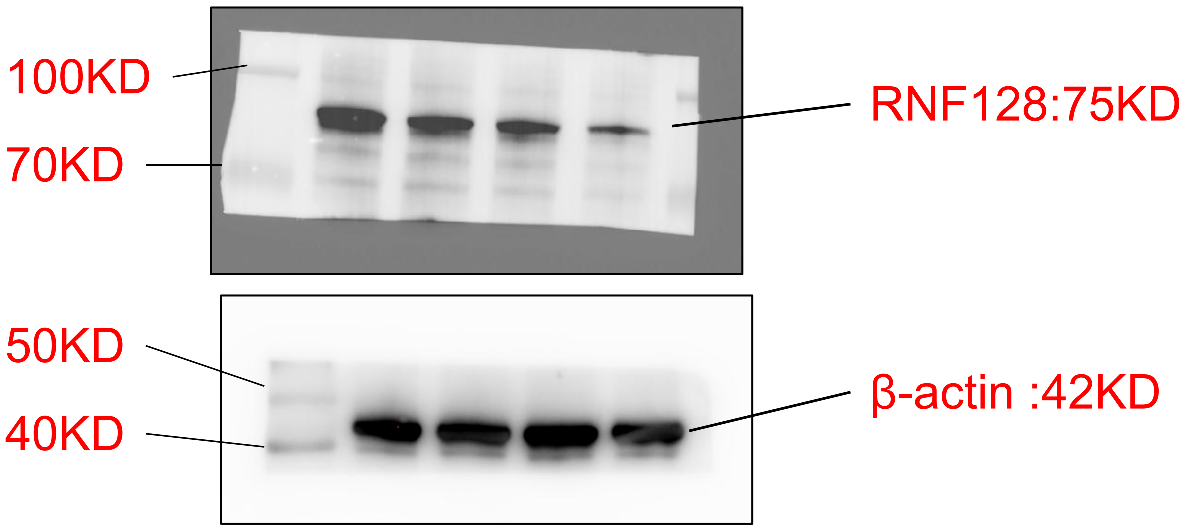

Repeat 1: **Repeat 2:**

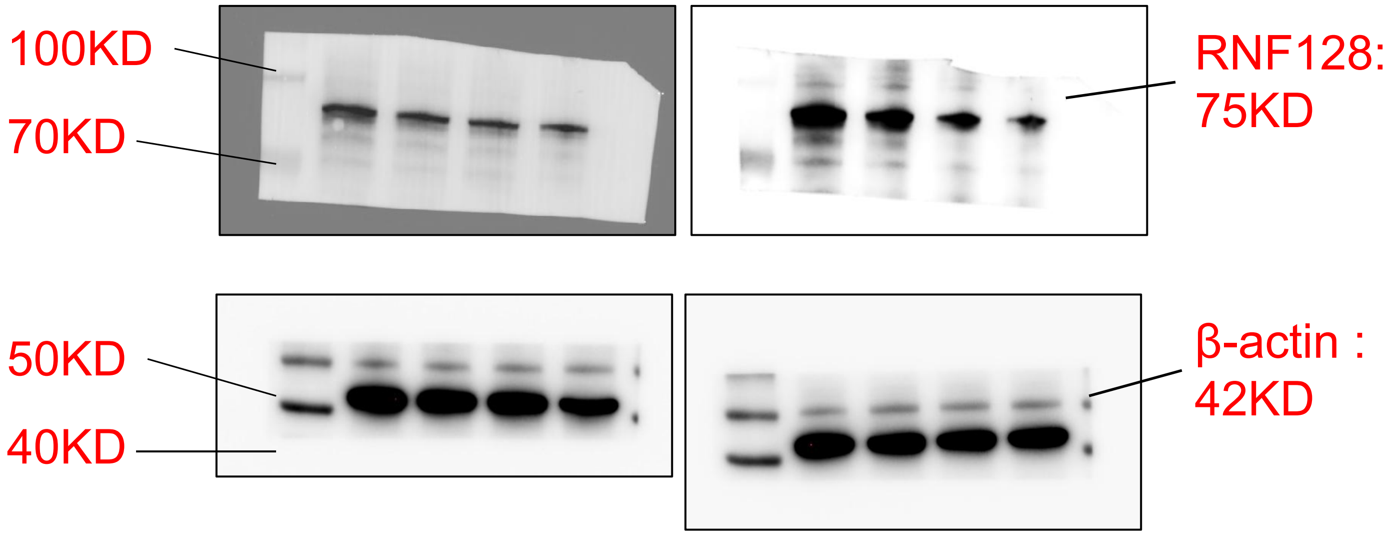

Figure S2 e

On fig:

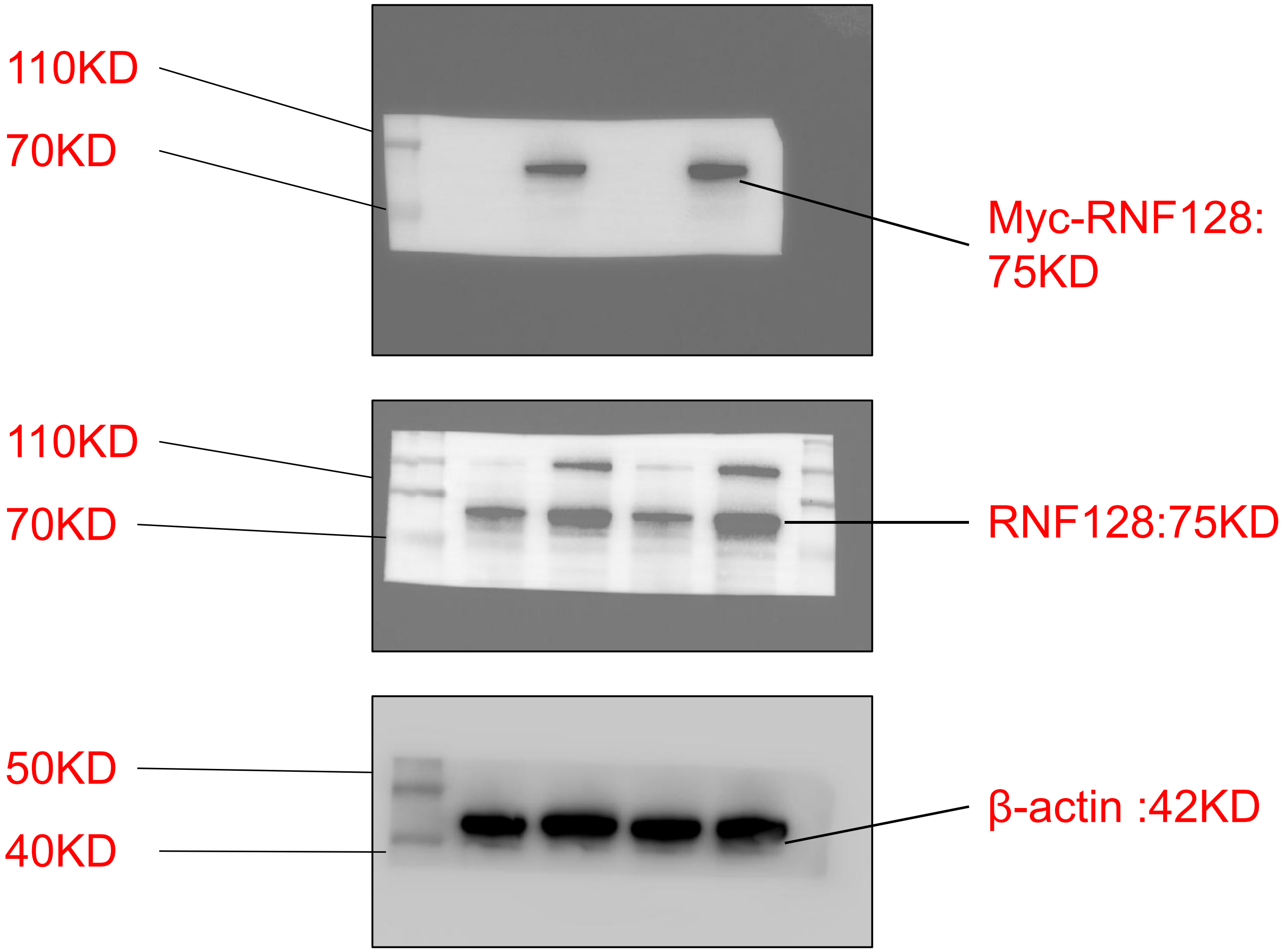

Figure S2 e

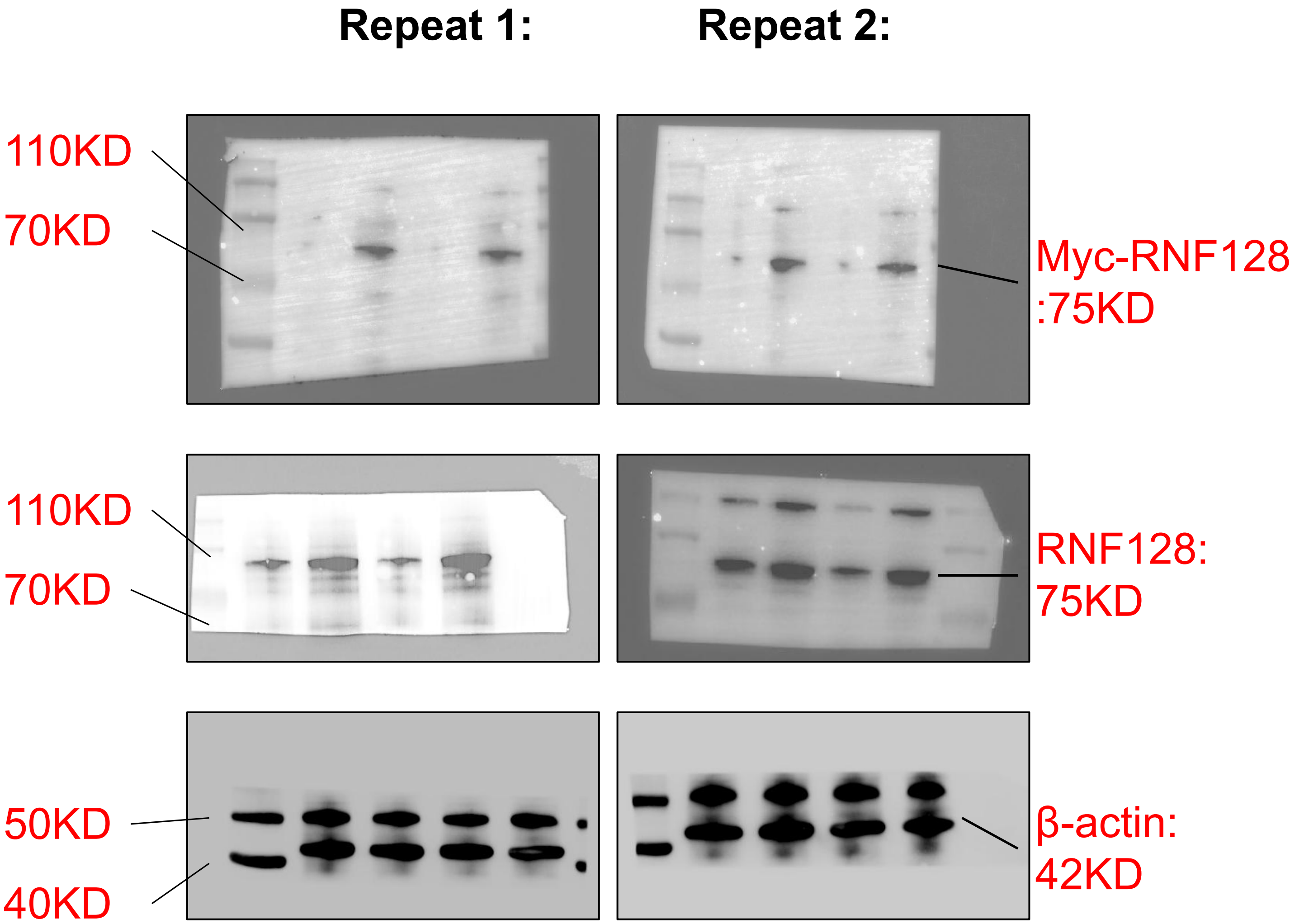

Repeat 1:
Repeat 2:
110KD
70KD
Myc-RNF128
:75KD
110KD
70KD
RNF128:
75KD
50KD
40KD
β-actin:
42KD

Figure S4 a

On fig:

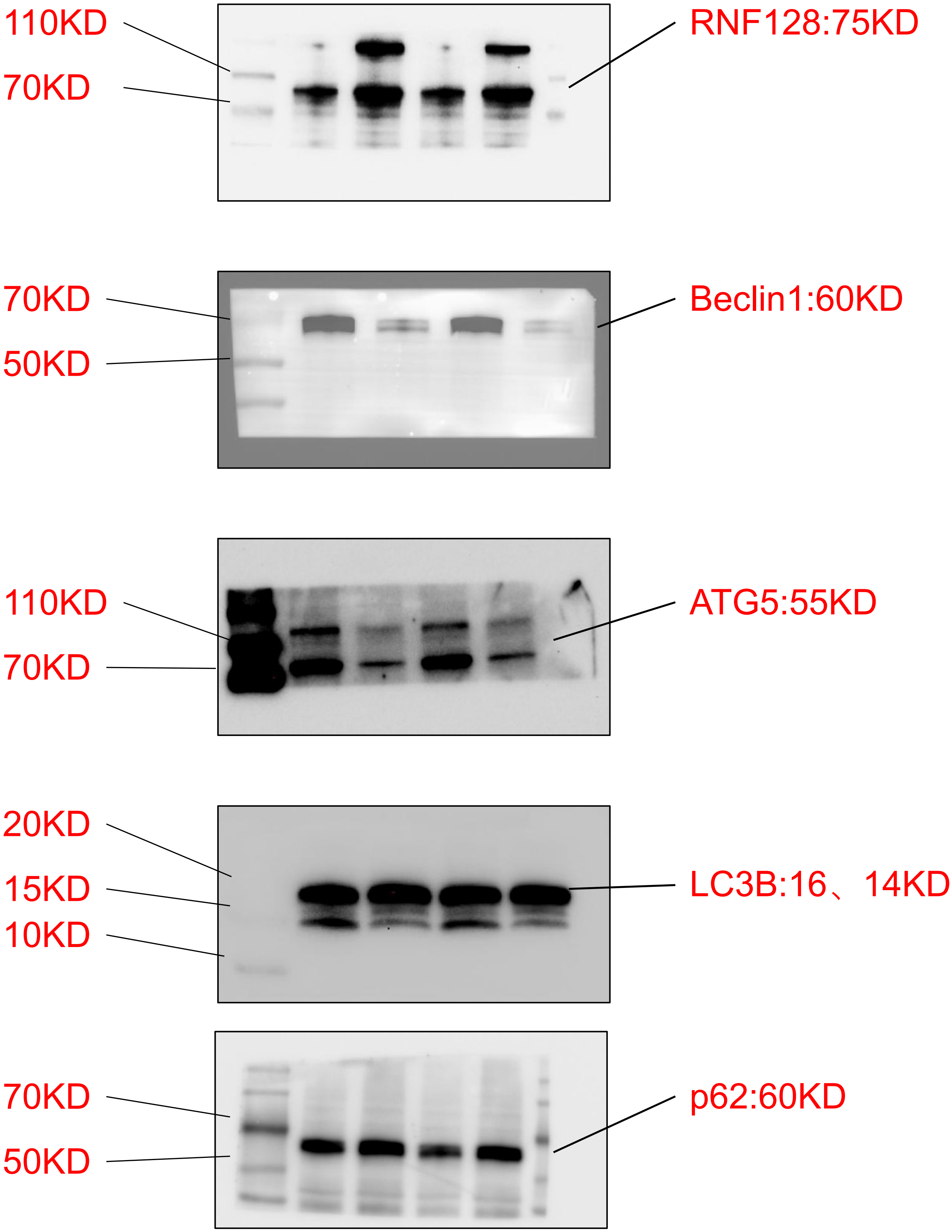

Figure S4 a

On fig:

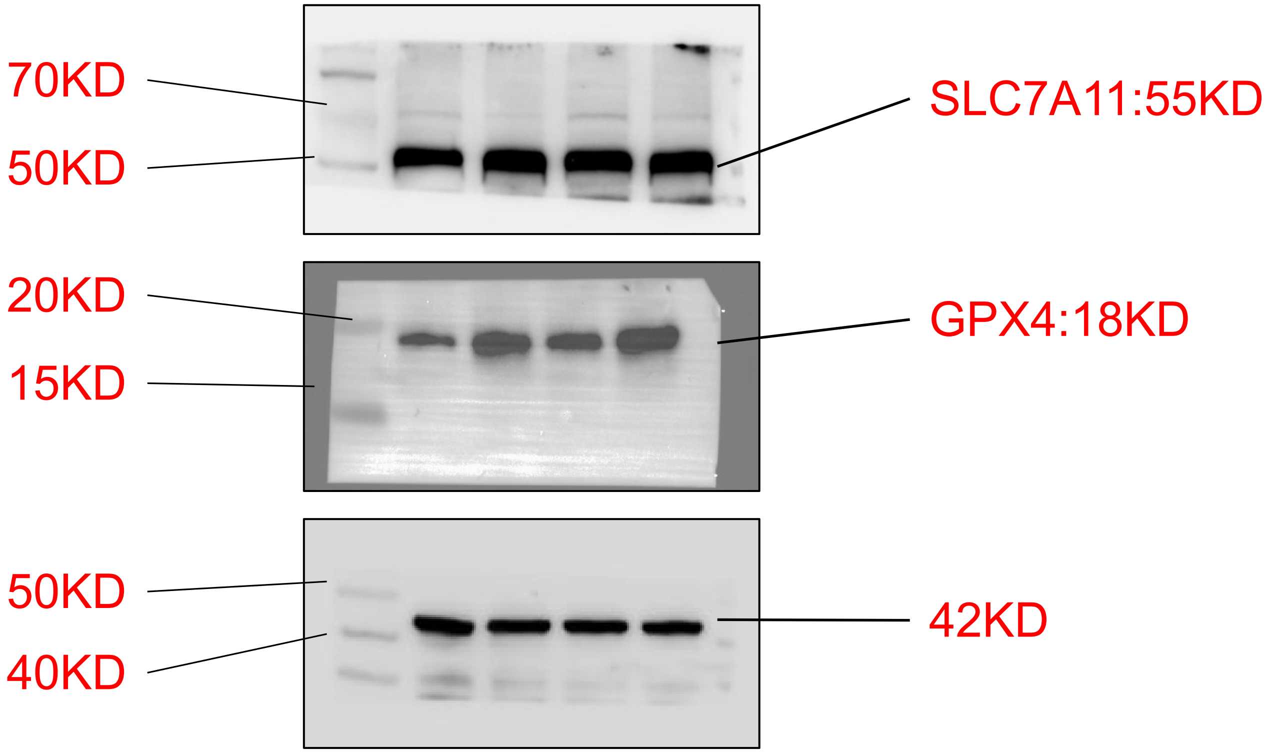

Figure S4 a

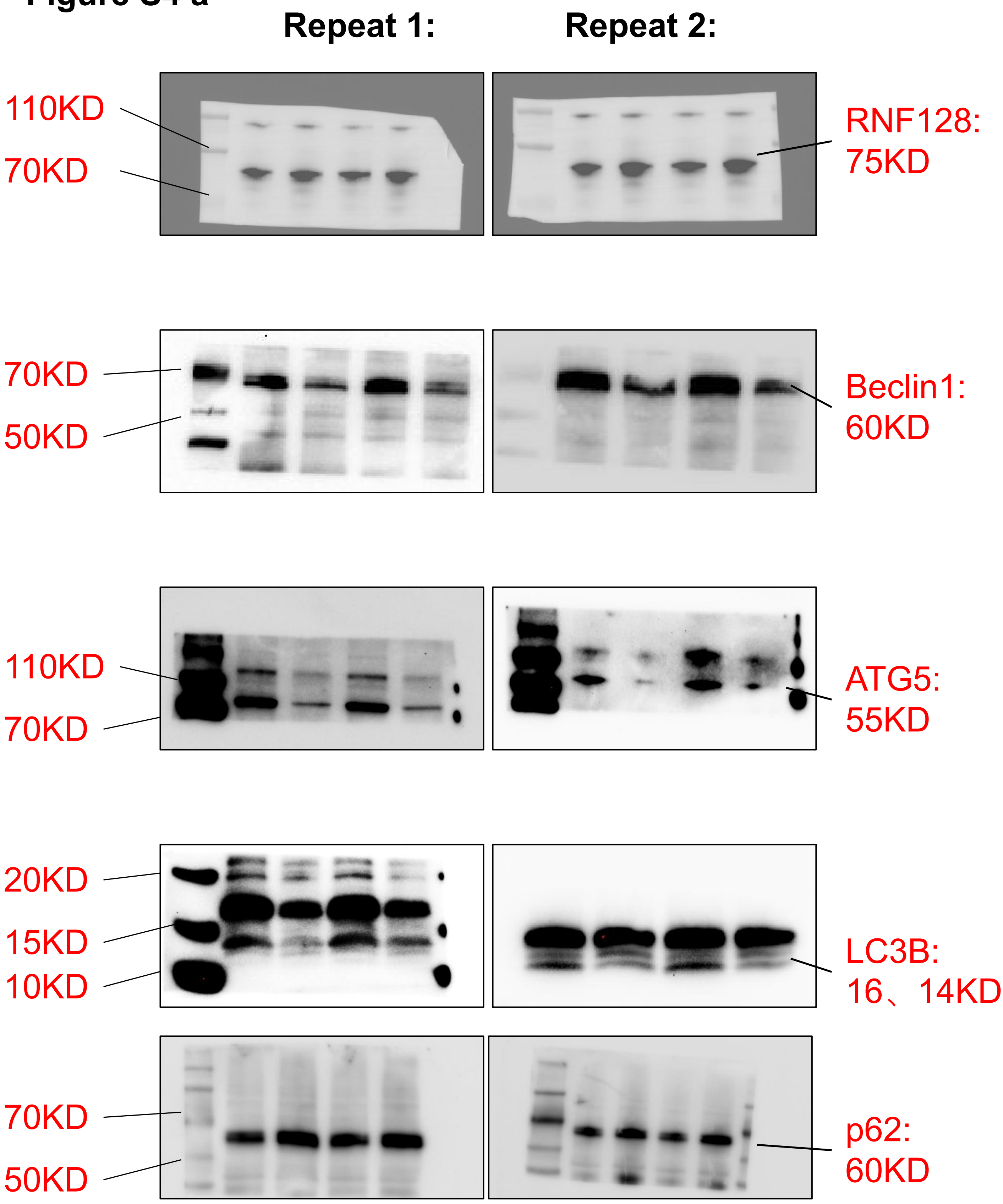

Repeat 1:
Repeat 2:
110KD
70KD
RNF128:
75KD
70KD
50KD
Beclin1:
60KD
110KD
70KD
ATG5:
55KD
20KD
15KD
10KD
LC3B:
16、14KD
70KD
50KD
p62:
60KD

Figure S4 a

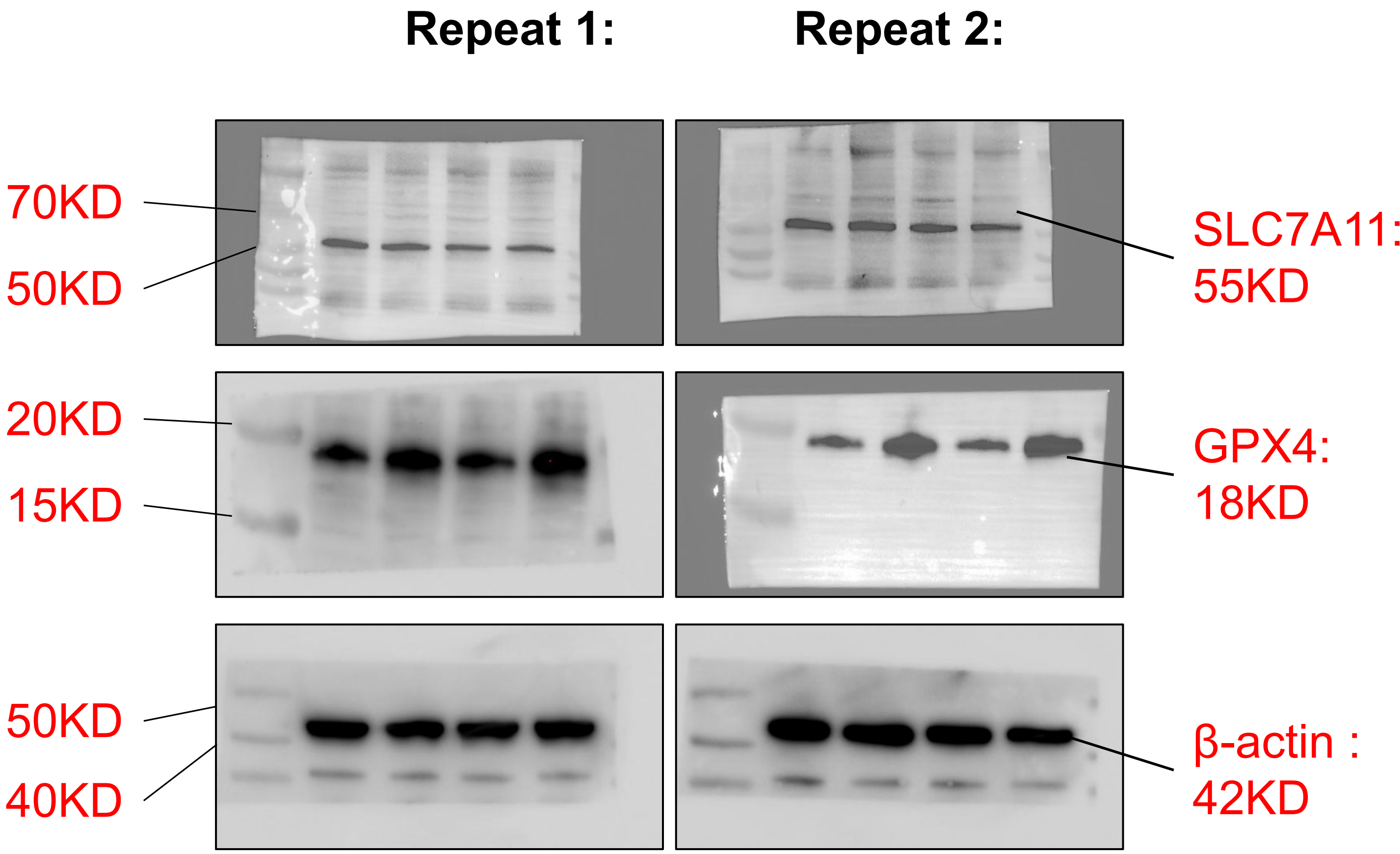

Repeat 1:
Repeat 2:
70KD
50KD
SLC7A11:
55KD
20KD
15KD
GPX4:
18KD
50KD
40KD
β-actin :
42KD

Figure S6 c

On fig:

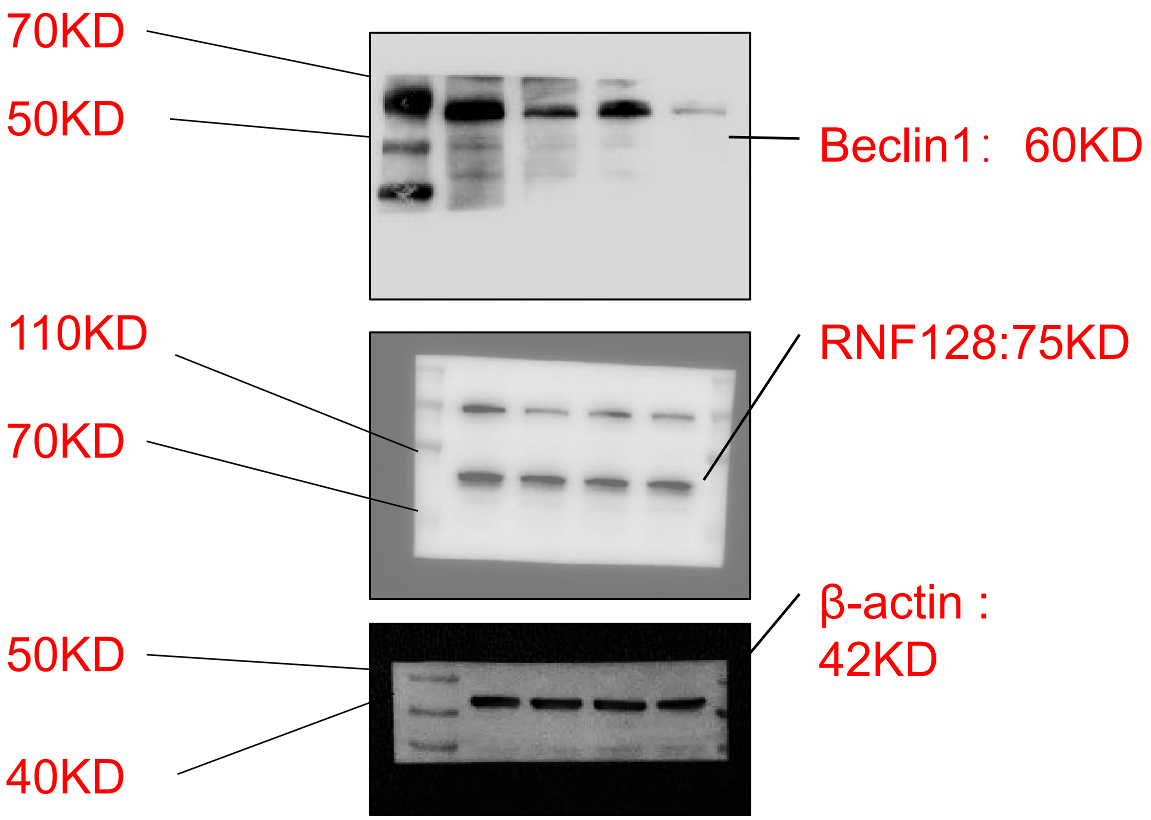

Repeat 1: **Repeat 2:**

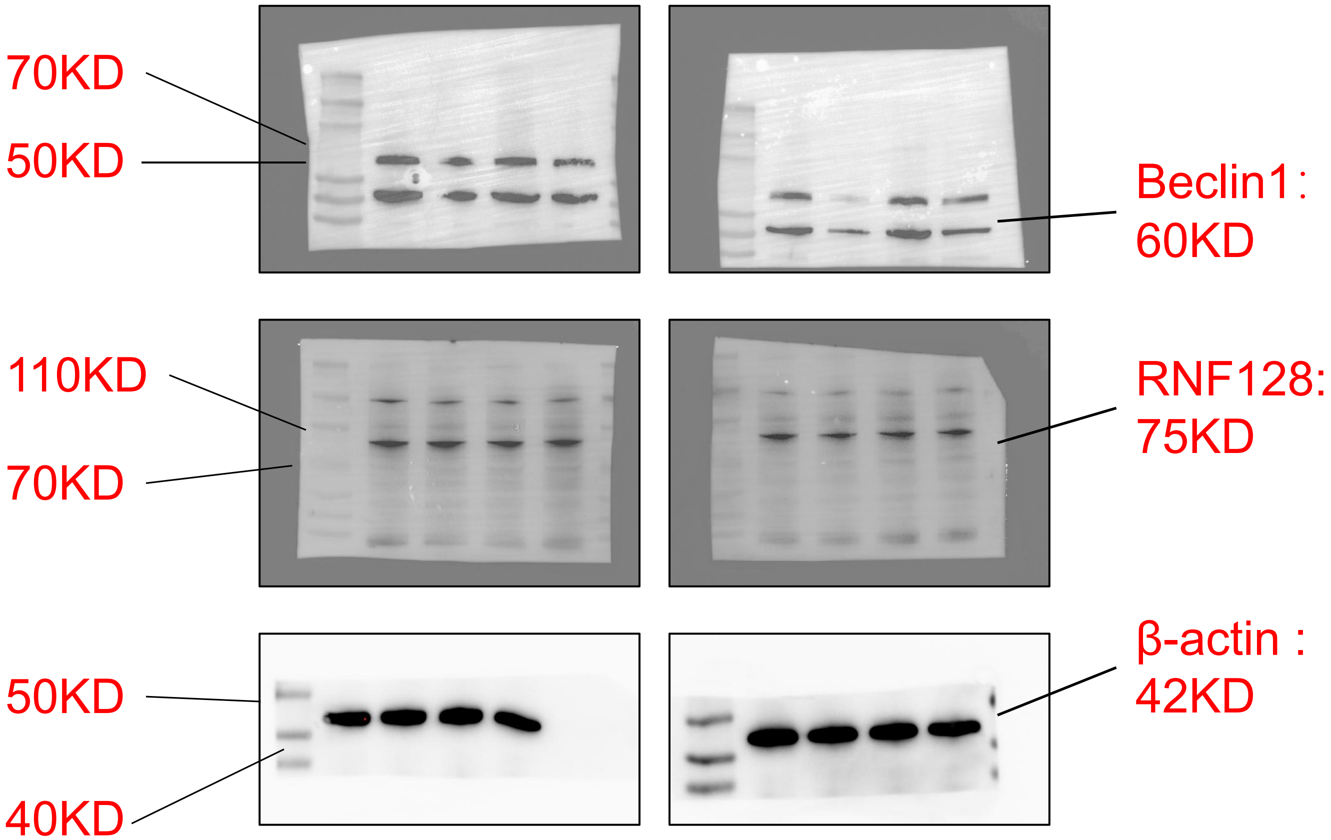

Figure S6 d

On fig:

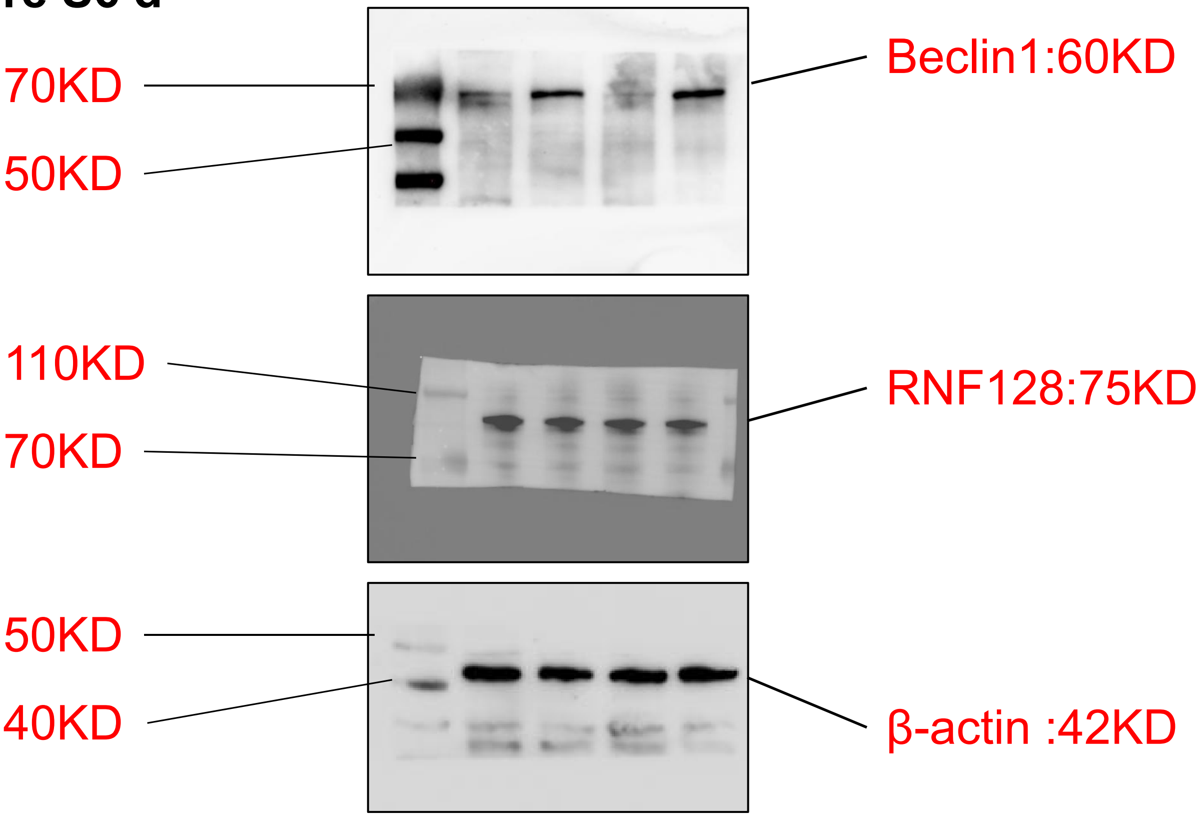

Repeat 1: **Repeat 2:**

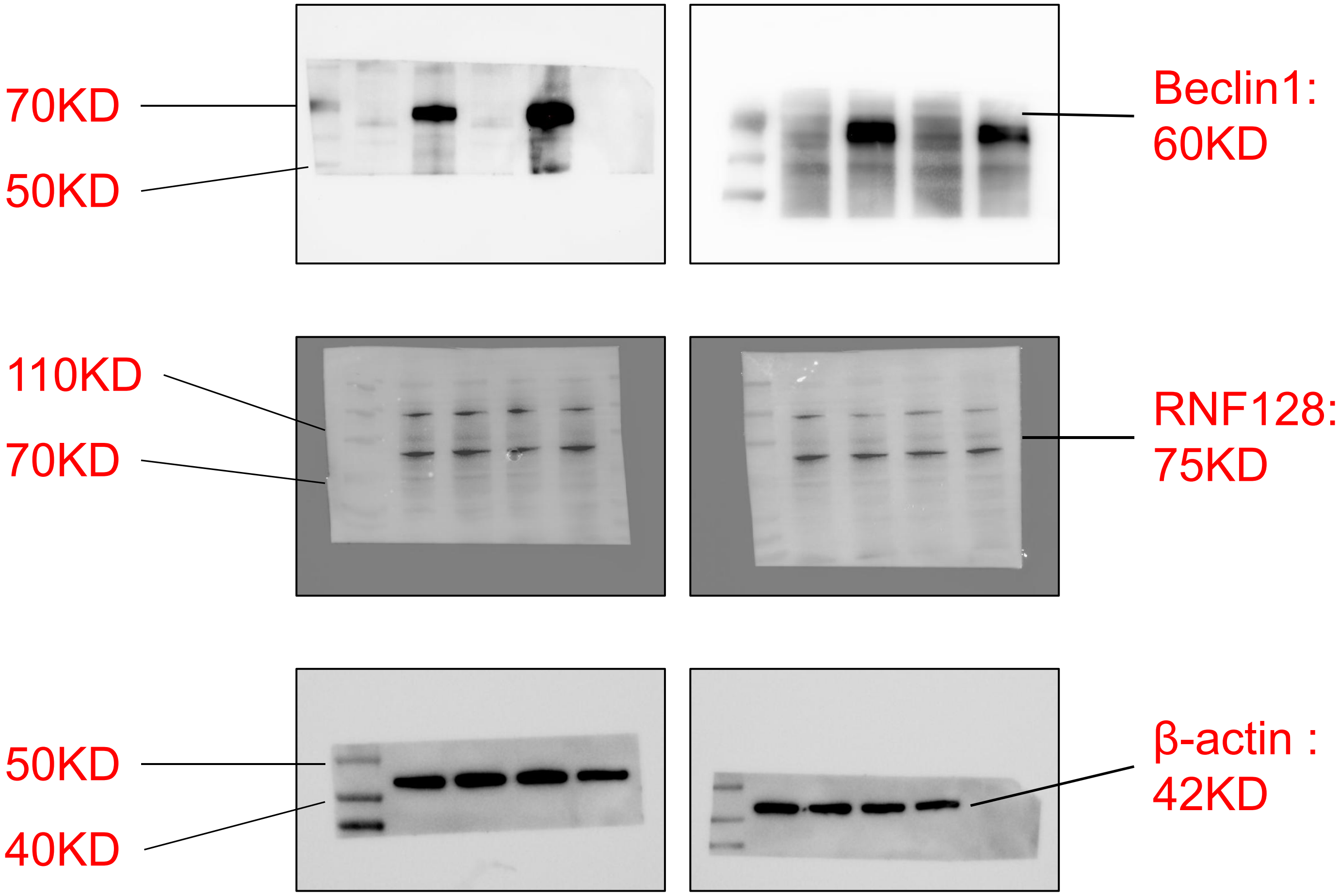

Figure S6 e

AGS:On fig:

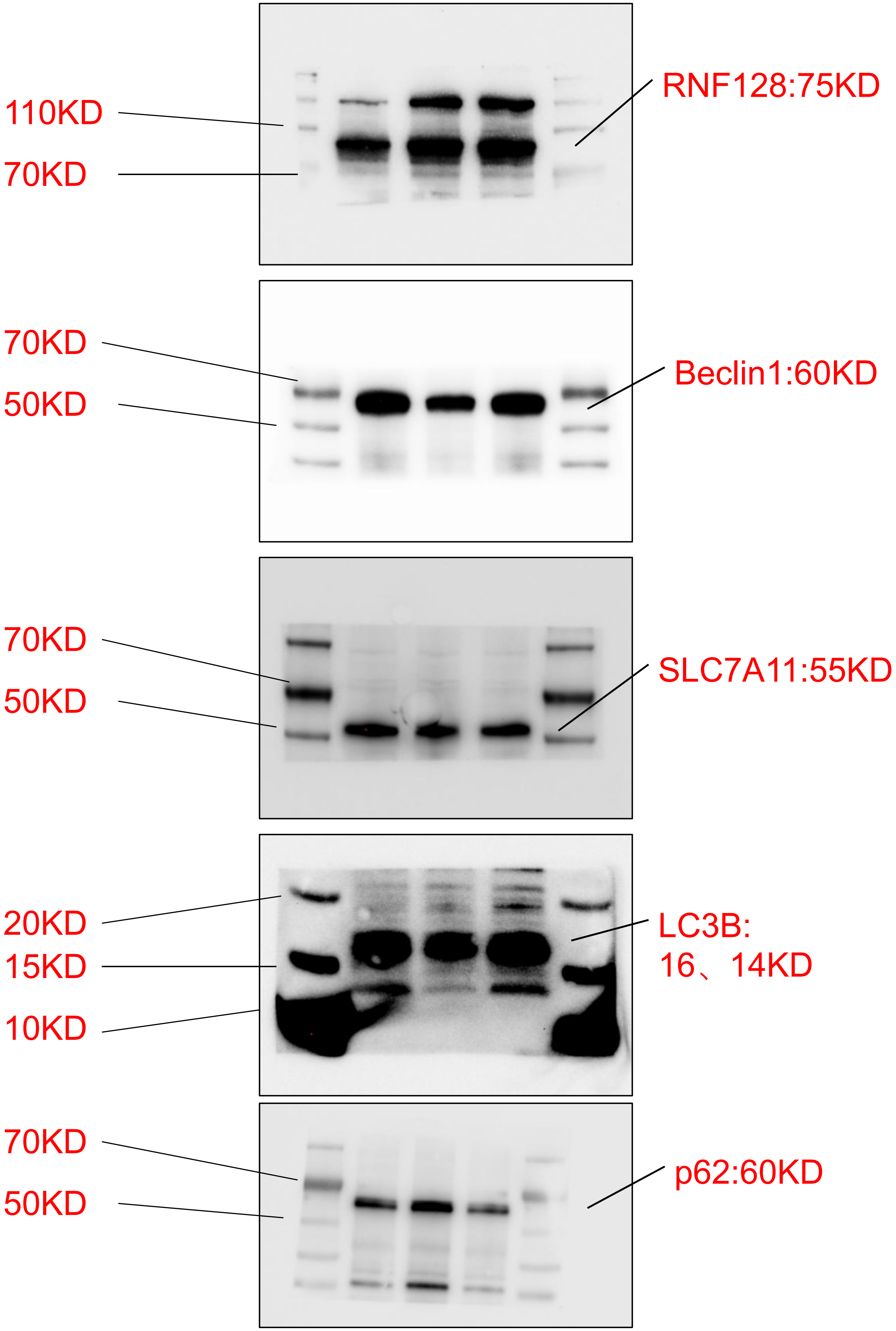

Figure S6 e

AGS:On fig:

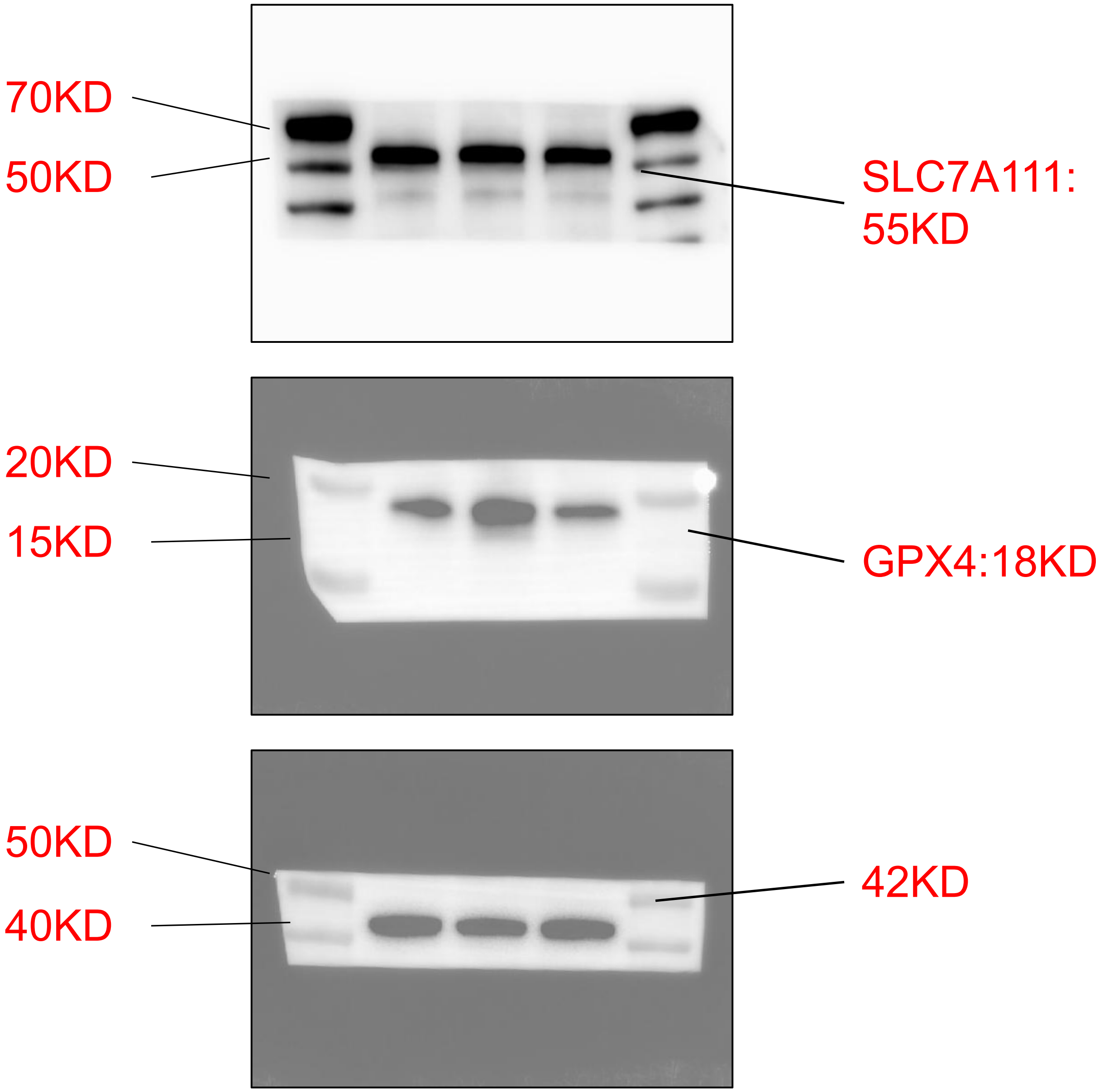

Figure S6 e

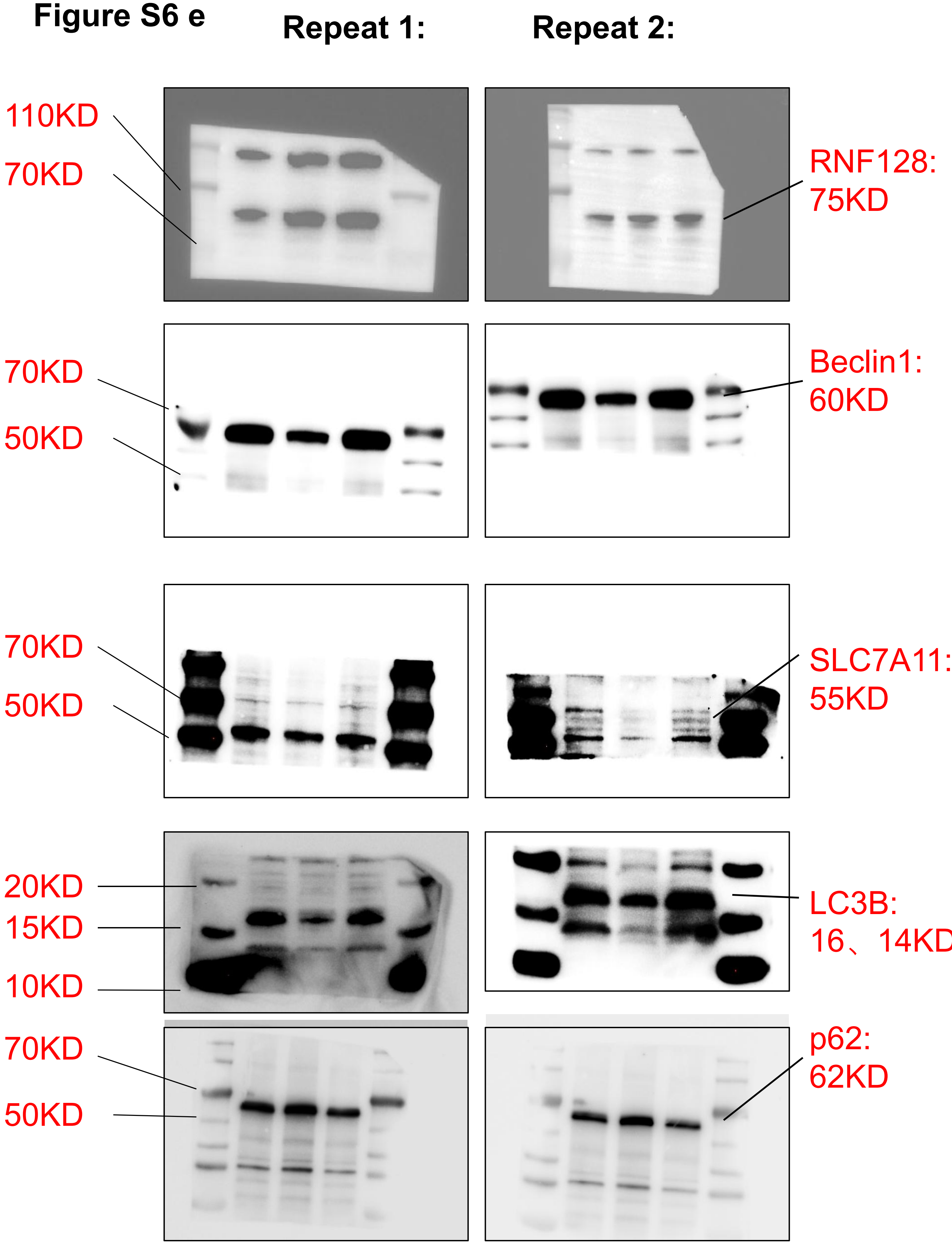
Repeat 1:
Repeat 2:
110KD
70KD
RNF128:
75KD
70KD
50KD
Beclin1:
60KD
70KD
50KD
SLC7A11:
55KD
20KD
15KD
10KD
LC3B:
16、14KD
70KD
50KD
p62:
62KD

Figure S6 e

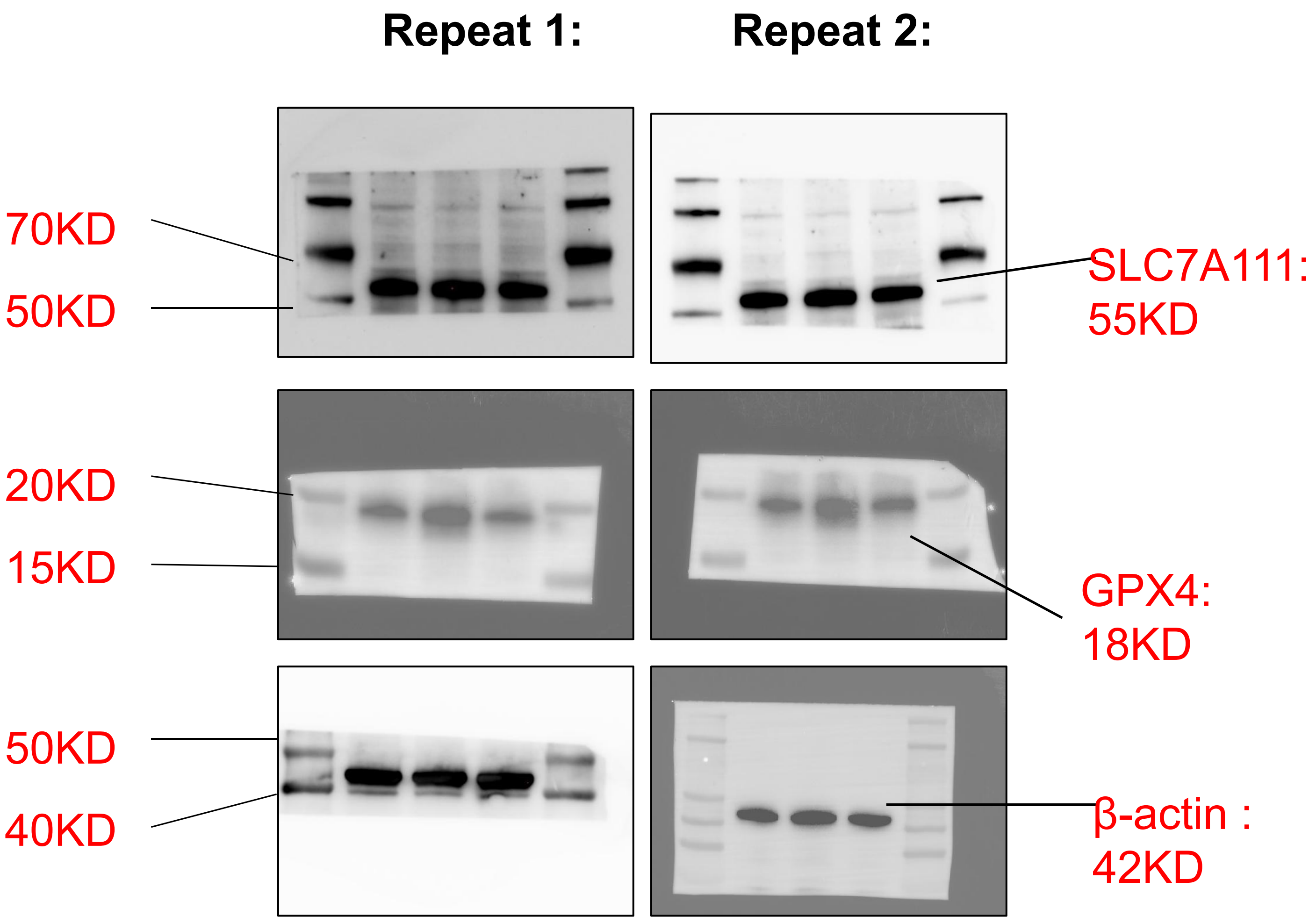
Repeat 1:
Repeat 2:
70KD
50KD
SLC7A111:
55KD
20KD
15KD
GPX4:
18KD
50KD
40KD
β-actin :
42KD

Figure S6 e

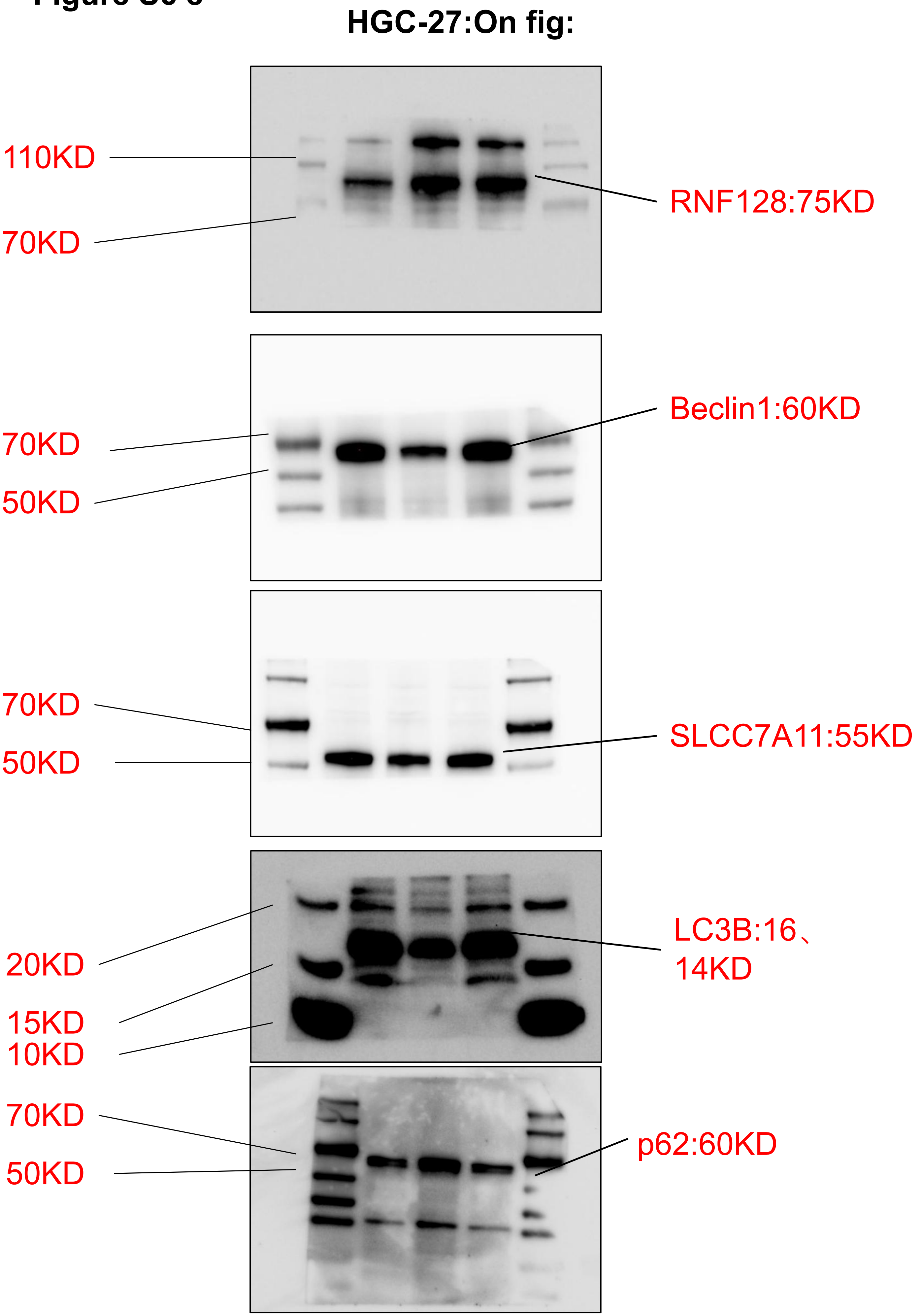
HGC-27:On fig:
110KD
70KD
RNF128:75KD
70KD
50KD
Beclin1:60KD
70KD
50KD
SLCC7A11:55KD
20KD
15KD
10KD
LC3B:16、
14KD
70KD
50KD
p62:60KD

Figure S6 e

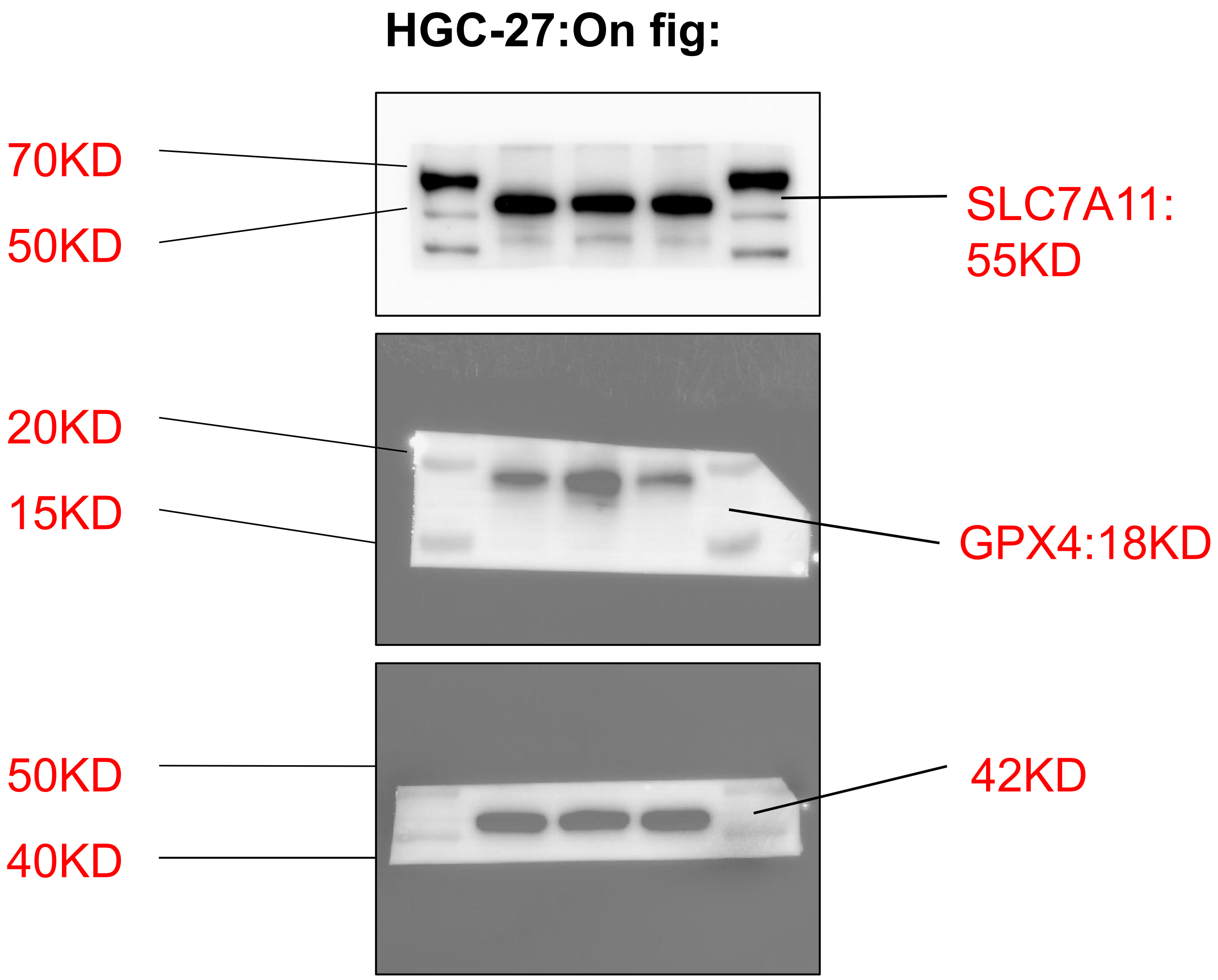
HGC-27:On fig:
70KD
50KD
SLC7A11:
55KD
20KD
15KD
GPX4:18KD
50KD
40KD
42KD

Figure S6 e

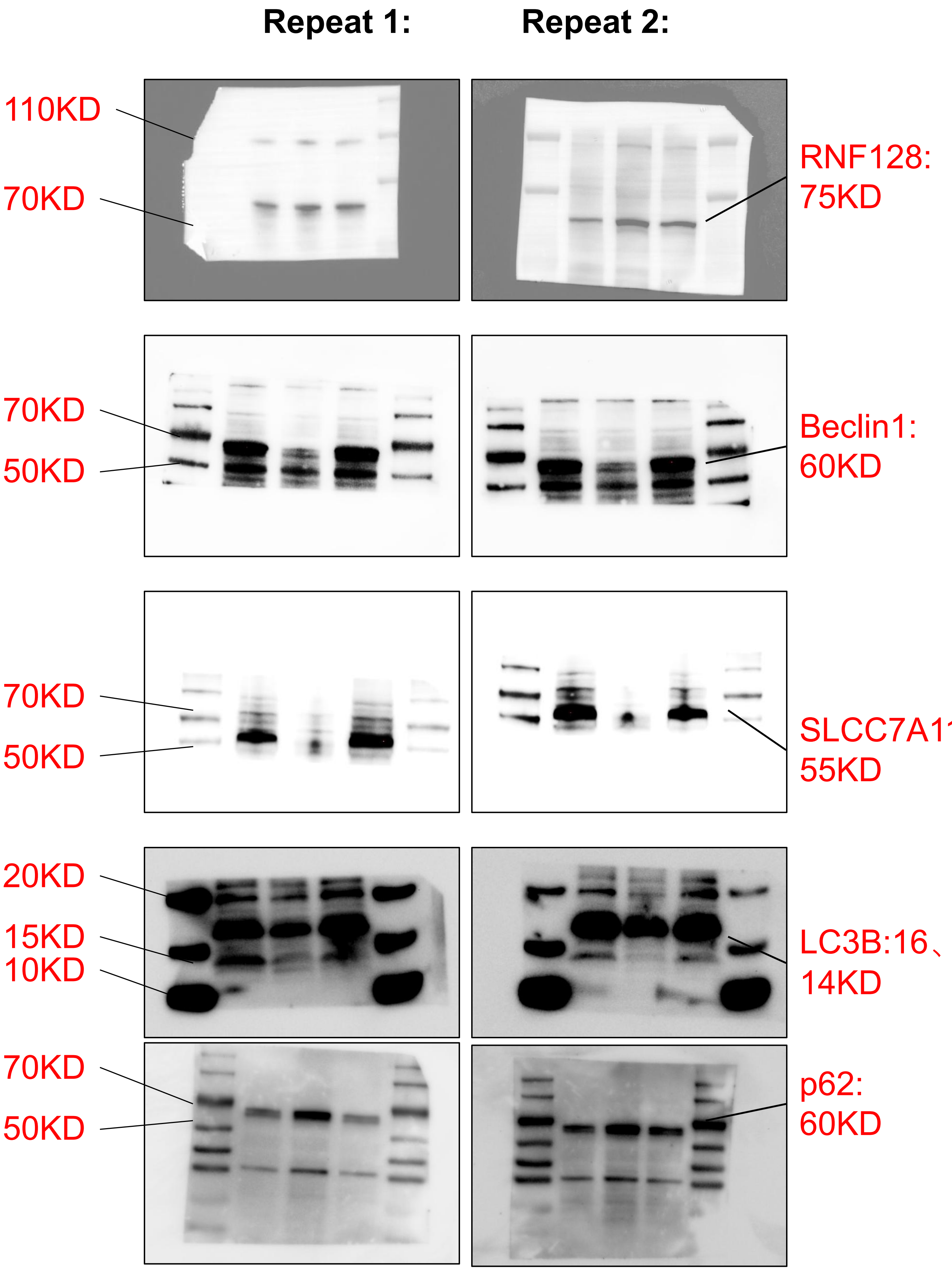
Repeat 1:
Repeat 2:
110KD
70KD
RNF128:
75KD
70KD
50KD
Beclin1:
60KD
70KD
50KD
SLCC7A11
55KD
20KD
15KD
10KD
LC3B:16、
14KD
70KD
50KD
p62:
60KD

Figure S6 e

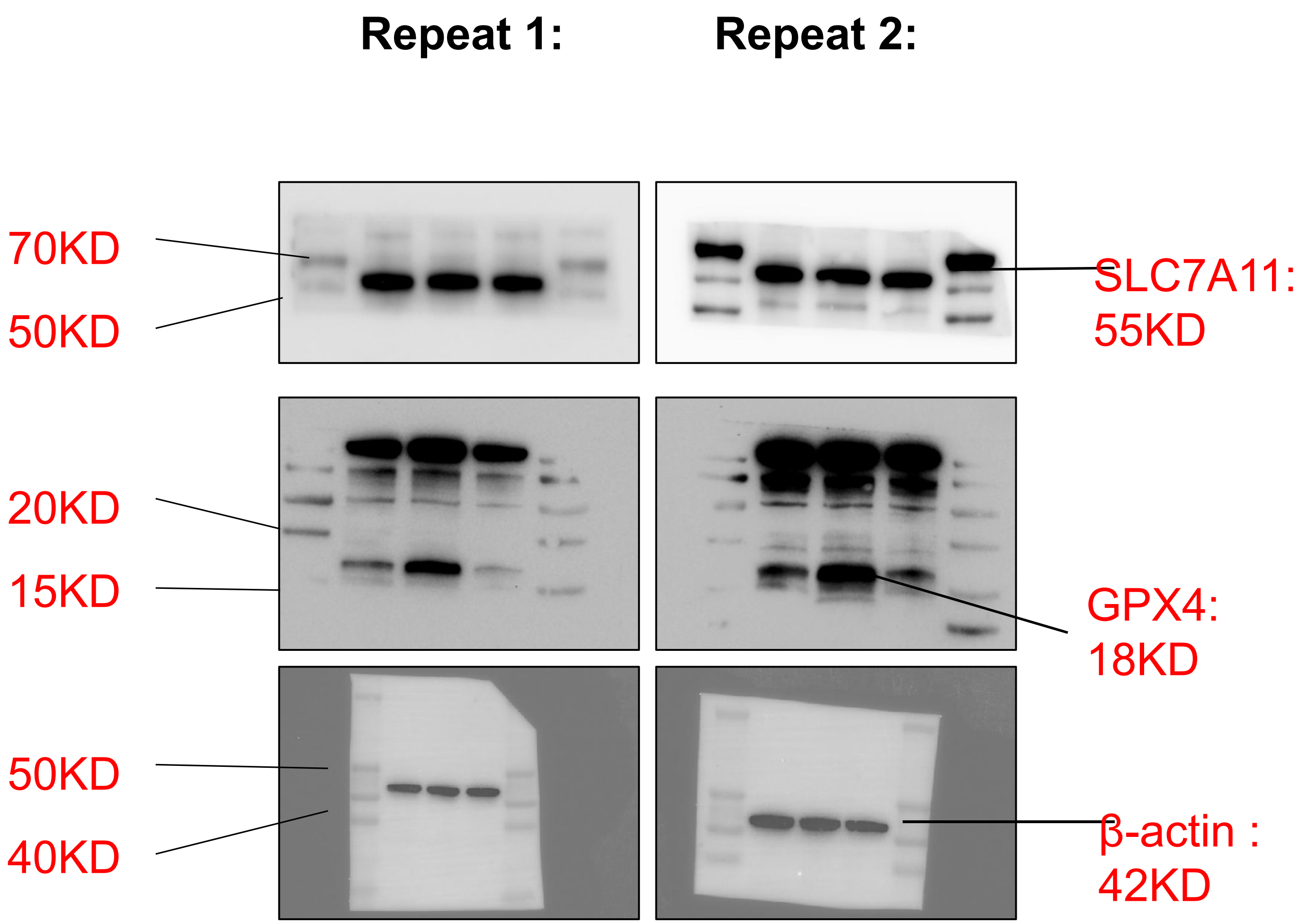
Repeat 1:
Repeat 2:
70KD
50KD
SLC7A11:
55KD
20KD
15KD
GPX4:
18KD
50KD
40KD
β-actin :
42KD
